# Systema: a framework for evaluating genetic perturbation response prediction beyond systematic variation

**DOI:** 10.1038/s41587-025-02777-8

**Published:** 2025-08-25

**Authors:** Ramon Viñas Torné, Maciej Wiatrak, Zoe Piran, Shuyang Fan, Liangze Jiang, Sarah A. Teichmann, Mor Nitzan, Maria Brbić

**Affiliations:** 1 School of Computer and Communication Sciences, EPFL, Lausanne, Switzerland; 2 Cambridge Stem Cell Institute, Jeffrey Cheah Biomedical Centre, University of Cambridge, Cambridge, UK; 3 Department of Medicine, University of Cambridge, Cambridge, UK; 4 School of Computer Science and Engineering, The Hebrew University of Jerusalem, Jerusalem, Israel; 5 CIFAR Macmillan Multi-scale Human Programme, CIFAR, Toronto, Canada; 6 Racah Institute of Physics, The Hebrew University of Jerusalem, Jerusalem, Israel; 7 Faculty of Medicine, The Hebrew University of Jerusalem, Jerusalem, Israel; 8 School of Life Sciences, EPFL, Lausanne, Switzerland; 9 Swiss Institute of Bioinformatics, Lausanne, Switzerland

**Keywords:** Machine learning, Computational models, Computer science, Gene expression, Genomic engineering

## Abstract

Predicting transcriptional responses to genetic perturbations is challenging in functional genomics. While recent methods aim to infer effects of untested perturbations, their true predictive power remains unclear. Here, we show that current methods struggle to generalize beyond systematic variation, the consistent transcriptional differences between perturbed and control cells arising from selection biases or confounders. We quantify this variation in ten datasets, spanning three technologies and five cell lines, and show that common metrics are susceptible to these biases, leading to overestimated performance. To address this, we introduce Systema, an evaluation framework that emphasizes perturbation-specific effects and identifies predictions that correctly reconstruct the perturbation landscape. Using this framework, we uncover insights into the predictive capabilities of existing methods and show that predicting responses to unseen perturbations is substantially harder than standard metrics suggest. Our work highlights the importance of heterogeneous gene panels and disentangles predictive performance from systematic effects, enabling biologically meaningful developments in perturbation response modeling.

## Abstract (web summary)

An evaluation framework isolates perturbation-specific effects in perturbation datasets.

## Main

Understanding how genetic perturbations affect single cells is crucial for advancing functional genomics. It has wide-ranging implications, including revealing gene functions, mapping regulatory networks, cell engineering and accelerating therapeutic discovery. Recent advances in high-throughput perturbation screening technologies have enabled unprecedented opportunities to systematically investigate the effects of perturbations in diverse cellular contexts; however, the space of possible perturbations is combinatorially complex, making exhaustive experimental exploration infeasible.

To navigate the complex combinatorial landscape of perturbations, computational approaches have been developed to predict transcriptional outcomes of genetic perturbations that were never experimentally tested^1–3^. These methods leverage previous knowledge derived from biological networks^1^ or large-scale single-cell atlases^2^ and use complex deep-learning architectures to infer post-perturbation expression profiles for unseen genetic perturbations. Recent studies have investigated considerably simpler approaches, including linear models^4–6^ and nonparametric baselines^7–11^, showing that they attain comparable predictive performance^7–9^. This raises a fundamental question: to what extent are existing perturbation response prediction methods learning the perturbation biology of single cells?

Here, we study the ability of perturbation response prediction methods to infer perturbation-specific effects for genetic perturbations not seen during training. We benchmark existing state-of-the-art methods across ten perturbation datasets and compare them to baselines that exclusively capture average treatment effects. Using standard evaluation metrics, we find that these simple baselines surprisingly perform comparably to the state-of-the-art methods. We show that this result can be largely explained by systematic variation (systematic differences between perturbed and control cells caused by selection biases in the perturbation panel or underlying biological factors). We introduce a measure to quantify the degree of systematic variation in perturbation datasets, and we posit that existing metrics are susceptible to these systematic biases, which can often lead to misleading conclusions. To gain insight into the biology captured by perturbation response prediction methods, we introduce Systema, a new evaluation framework that (1) mitigates systematic biases by focusing on perturbation-specific effects, and (2) provides an interpretable readout of the methods’ ability to reconstruct the perturbation landscape. Systema reveals that while generalizing to unseen perturbations remains a substantial challenge, some methods can partially recover the effects of perturbations targeting functionally coherent gene groups. Systema can help differentiate predictions that merely replicate systematic effects from those that capture biologically informative perturbation responses. Systema is available on GitHub at https://github.com/mlbio-epfl/systema (ref. ^12^).

## Results

### Simple baselines perform comparably to existing perturbation response prediction approaches

**Fig. 1:**
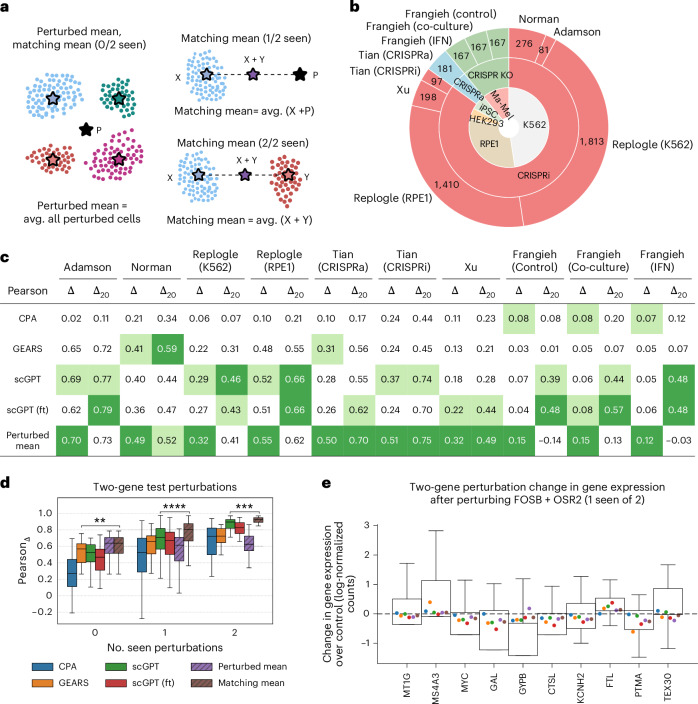
Simple baselines perform similar or better than state-of-the-art perturbation response prediction methods. **a**, Overview of the perturbed mean and matching mean baselines. The perturbed mean is the average of all perturbed cells in the train set, which we denote as *P*. The matching mean for perturbation *X* + *Y* is the average of the *X* and *Y* centroids (Methods). When *X* or *Y* are unseen at train time, their centroid is replaced by the perturbed mean. Image created in BioRender. **b**, Summary of datasets included in our benchmark. Outer ring shows total number of perturbations after processing (Methods). Middle ring shows perturbation technology. Inner ring shows cell line. **c**, Performance comparison of perturbed mean baseline and state-of-the-art methods on unseen one-gene perturbations. We evaluated performance on ten single-cell perturbation datasets from six different sources^13–18^ using the Pearson correlation coefficient Pearson_*Δ*_ computed on differential expression profiles (Methods), using all genes (*Δ*) and the top 20 differentially expressed genes of each perturbation (*Δ*20). We report the average test performance across three independent runs with different data splits (*n* represents the number of test perturbations across all three independent runs, Adamson: *n* = 63, Norman: *n* = 108, Replogle K562: *n* = 1,362, Replogle RPE1: *n* = 1,059, Tian CRISPRa: *n* = 75, Tian CRISPRi: *n* = 138, Xu: *n* = 150, Frangieh control: *n* = 126, Frangieh co-culture: *n* = 126, Frangieh interferon: *n* = 126). Dark and light green colors highlight the best and second-best performing methods, respectively. **d**, Pearson_*Δ*_ correlation on unseen two-gene perturbations of the Norman^14^ dataset, applied to average differential expression profiles over control (predicted versus ground truth). Results across three independent runs with different data splits (0-seen: *n* = 39, 1-seen: *n* = 147, 2-seen: *n* = 39). Boxes depict distribution quartiles, with the center line corresponding to the median, and whiskers span 1.5 × interquartile range. We compared the two methods with highest median scores using two-sided paired *t*-tests performed separately for each independent run. The resulting *P* values were aggregated using Fisher’s method. *P* values, 0-seen, 0.009; 1-seen, 2.6 × 10^−12^; 2-seen, 0.0009. NS, not significant, *P* > 0.05; *P ≤0.05, **P ≤ 0.01, ***P ≤ 0.001, ****P ≤ 0.0001. **e**, Mean change in expression predicted by different methods for the combinatorial *FOSB*+*OSR2* perturbation. Only the perturbation *OSR2* was observed at train time. Boxes indicate experimentally measured differential expression (*n* = 324 cells). Boxes depict distribution quartiles and whiskers span 1.5 × interquartile range. The dashed line denotes the average control expression. Dot colors represent different baselines (legend in **d**).

We benchmarked established perturbation response methods on the task of predicting transcriptional outcomes of unseen genetic perturbations. We considered three state-of-the-art perturbation response prediction methods: compositional perturbation autoencoder (CPA)^10^, GEARS^1^ and scGPT^2^ (Methods). While CPA was not specifically designed for generalizing across unseen genetic perturbations (Methods), it exemplifies a baseline that operates without prior knowledge. In addition, we designed two simple nonparametric baselines that capture average perturbation effects: (1) average expression across all perturbed cells, which we refer to as perturbed mean, and (2) average expression across matched post-perturbation profiles for combinatorial perturbations, referred to as matching mean (Methods and Fig. 1a). Specifically, the matching mean for perturbation *X* + *Y* is the average of the *X* and *Y* centroids, and if *X* or *Y* are unseen at train time, their centroid is replaced by the perturbed mean. For one-gene perturbations, the matching mean and the perturbed mean are equivalent. We evaluated the baselines on data from ten single-cell perturbation datasets collected from six different sources (refs. ^13–18^, Methods and Fig. 1b). Our benchmark spans three distinct technologies, five different cell lines and varying numbers of perturbations, including a genome-wide perturbation screen^15^ and a dataset with combinatorial two-gene perturbations^14^.

We studied to what extent methods can predict the transcriptional changes induced by unseen genetic perturbations, defined as the average difference in gene expression between perturbed cells subjected to a given perturbation and control cells (the average treatment effect). To evaluate performance, we employed different metrics previously considered in the literature^1,2^, including the Pearson correlation between ground truth and predicted expression changes, using all genes (Pearson_*Δ*_) and the top 20 differentially expressed genes (Pearson_*Δ*20_) (Methods). These metrics assess how well different methods can predict the transcriptional changes of a perturbation with respect to the population of control cells. We found that the two simple baselines performed comparatively or outperformed state-of-the-art methods across different datasets and evaluation metrics (Fig. 1c and Supplementary Figs. 1–3). For unseen one-gene perturbations, using the Pearson_*Δ*_ score, the perturbed mean baseline outperformed other methods across all datasets. Using the Pearson_*Δ*20_ metric, it achieved higher or comparable performance across all datasets except Frangieh^18^, where scGPT outperformed all other methods. We observed a similar trend when using other evaluation metrics such as root mean-squared error (RMSE) (Supplementary Fig. 3).

We next evaluated performance on the task of predicting transcriptional responses to two-gene perturbations in the Norman^14^ dataset, the only dataset with combinatorial perturbations in our benchmark. For unseen two-gene perturbations (two-gene perturbations where none of the individual perturbations was observed at train time), the matching mean baseline outperformed all other baselines by a considerable margin, with relative improvements of 11% for Pearson_*Δ*_ over the best alternative method (GEARS; Fig. 1d). As expected, the two-gene performance improved as the number of matching one-gene perturbations seen at train time increased, highlighting the advantage of methods capable of recalling or approximating the one-gene perturbation responses of the matching genes. The simple baselines also matched the performance of existing methods in terms of Pearson_*Δ*20_ and RMSE metrics (Supplementary Figs. 4 and 5). We noted that the predicted differential expression profiles (the average expression changes with respect to control cells) were similar to each other and correlated with those of the perturbed mean (Fig. 1e and Supplementary Figs. 6 and 7). These results suggest that perturbation response prediction methods predominantly capture systematic differences between control and perturbed cells and may fail to uncover perturbation-specific effects.

### Systematic differences between control and perturbed cells lead to high predictive scores

We hypothesized that the comparatively high predictive scores of these baselines reflect systematic differences between perturbed and control cells. The presence of systematic differences that explain variation in the gene expression of single cells is prevalent across datasets^19,20^ and can greatly affect downstream analyses. For example, systematic variation can lead to overestimated predictive performance of perturbation response models when they primarily capture the average perturbation effect, obscuring their ability to generalize to novel perturbations. In perturbation datasets, systematic differences between perturbed and control cells may be explained by potential selection biases, confounding variables or underlying biological factors. For example, perturbing a panel of functionally related genes will lead to transcriptomic profiles that are consistently different between perturbed and control cells. Additionally, unmeasured variables such as cell-cycle phase, chromatin landscape and target efficiency may strongly influence post-perturbation profiles^21^. Systematic variation may still arise when responses that are biological in origin but systematic in effect, such as stress response or cell-cycle arrest, occur broadly across many perturbations.

**Fig. 2:**
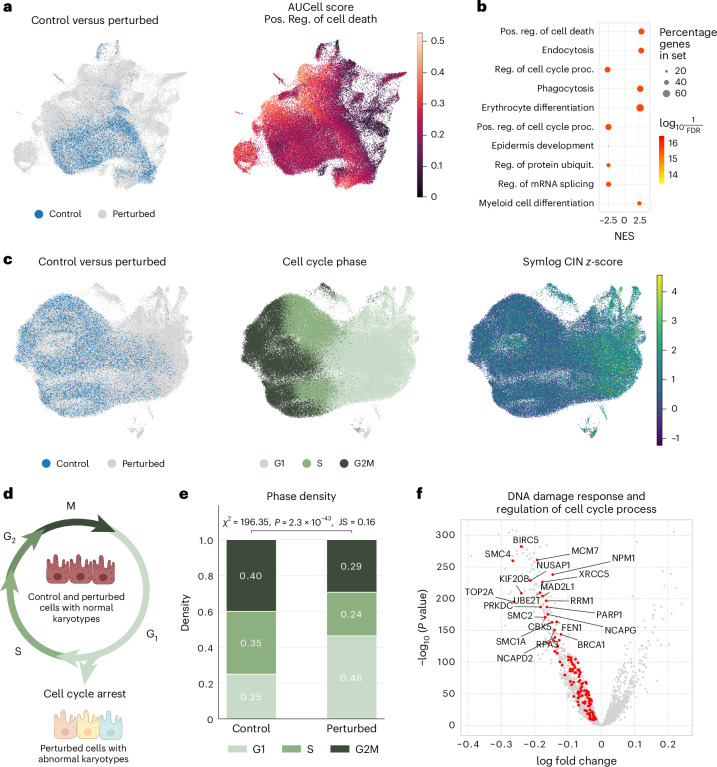
Systematic variation in the Norman^14^ and Replogle^15^ perturbation datasets. **a**, Uniform Manifold Approximation and Projection (UMAP) plot of normalized gene expression values in the Norman^14^ dataset colored by control versus perturbed cells and AUCell scores of positive regulation of response to external stimulus and response to positive regulation of cell death. Control cells correspond to cells with nontargeting guides. **b**, Normalized enrichment scores and false discovery rate for top enriched pathways in the Norman^14^ dataset. We applied Gene Set Enrichment Analyses using Biological Process 2023 gene sets from the Gene Ontology (GO)^28,29^ to compare the population of perturbed cells with the population of control cells. **c**, UMAP plot of normalized gene expression values in the Replogle^15^ (RPE1) dataset colored by control versus perturbed cells, cell-cycle phase and CIN *z*-score of each perturbation. The perturbation CIN score was calculated as the mean single-cell sum of squared CIN values, *z*-normalized relative to control perturbations^15^. Control cells correspond to cells with nontargeting guides. **d**, Cell-cycle diagram. Perturbations that induce chromosomal instability are more likely to trigger cell-cycle arrest. Image created in BioRender. **e**, Phase density of control and perturbed cells in the Replogle^15^ (RPE1) dataset. The density of cells in G1 is significantly higher for perturbed cells with a *P* value of 2.3 × 10^−43^. JS, Jensen–Shannon divergence. **f**, Volcano plot depicting the log-fold changes (perturbed versus control cells) in the Replogle^15^ (RPE1) dataset for gene sets involved in DNA damage response and regulation of cell-cycle process. We used a two-sided independent two-sample *t*-test without adjustments.

Given the experimental designs of the widely used Adamson^13^ and Norman^14^ datasets, which target genes from specific biological processes (endoplasmic reticulum homeostasis^13^ and cell cycle and growth^14^), we expected to observe systematic differences between perturbed and control cells in those datasets. To verify this, we performed gene set enrichment analysis (GSEA)^22^ to identify enriched pathways and used AUCell^23^ to score their activity in single cells. In the Adamson^13^ dataset, we observed systematic differences in activity scores between perturbed and control cells in terms of multiple pathways, including response to external stimuli, response to chemical stress and positive regulation of cell death (Supplementary Figs. 8 and 9). In the Norman^14^ dataset, we observed positive activation of cellular death and downregulation of stress response pathways in perturbed cells, including cellular response to heat and to unfolded protein (Fig. 2a, Supplementary Figs. 10–13 and Supplementary Table 1). Our analysis also highlighted the enrichment of pathways associated with cell cycle and erythrocyte differentiation, consistent with the fact that Norman et al.^14^ selected a perturbation panel targeting genes involved in these processes. While this reflects the expected biological consequences of the perturbations, it also introduces structured variation that may influence downstream analyses such as perturbation response prediction.

To investigate whether systematic variation exists in large-scale perturbation screens, we extended our analysis to the Replogle dataset^15^. In the Replogle^15^ RPE1 dataset, we observed a significant difference in the distribution of cells across cell-cycle phases between perturbed and control cells (Fig. 2c–e), with 46% of perturbed cells versus 25% of control cells in the G1 phase (Jensen–Shannon divergence of 0.16, chi-squared = 196.35, *P* = 2.3 × 10^−43^). We attribute this effect to the widespread chromosomal instability induced by perturbations; p53-positive RPE1 cells with abnormal karyotypes tend to react to instabilities through cell-cycle arrest^15^. In contrast, the differences in cell-cycle occupancy were considerably smaller on the genome-wide K562 perturbation dataset (Supplementary Figs. 14 and 15), likely due to the fact that K562 cells are p53-negative and, thus, less capable of responding to chromosomal abnormalities (for example, by triggering cell-cycle arrest). Application of GSEA between control and perturbed cells showed the negative enrichment of multiple pathways, including DNA repair and replication on the RPE1 dataset (Supplementary Figs. 16 and 17 and Supplementary Table 2) and ribosome biogenesis on the K562 dataset (Supplementary Fig. 18). Genes belonging to the enriched RPE1 pathways had consistently lower expression in perturbed cells than in control cells (Supplementary Fig. 19), reflecting the high prevalence of chromosomal instabilities among perturbed cells. Taken together, our observations highlight that commonly used single-cell perturbation datasets may contain systematic variation (whether stemming from biological effects, design choices or confounding factors) and that special care must be taken in modeling this data and evaluating performance.

### Standard reference-based metrics are susceptible to systematic variation

We next studied to what extent standard evaluation metrics are affected by systematic differences between perturbed and control cells. In presence of systematic variation, cells subjected to different perturbations may consistently exhibit similar gene expression shifts with respect to the population of control cells.

**Fig. 3:**
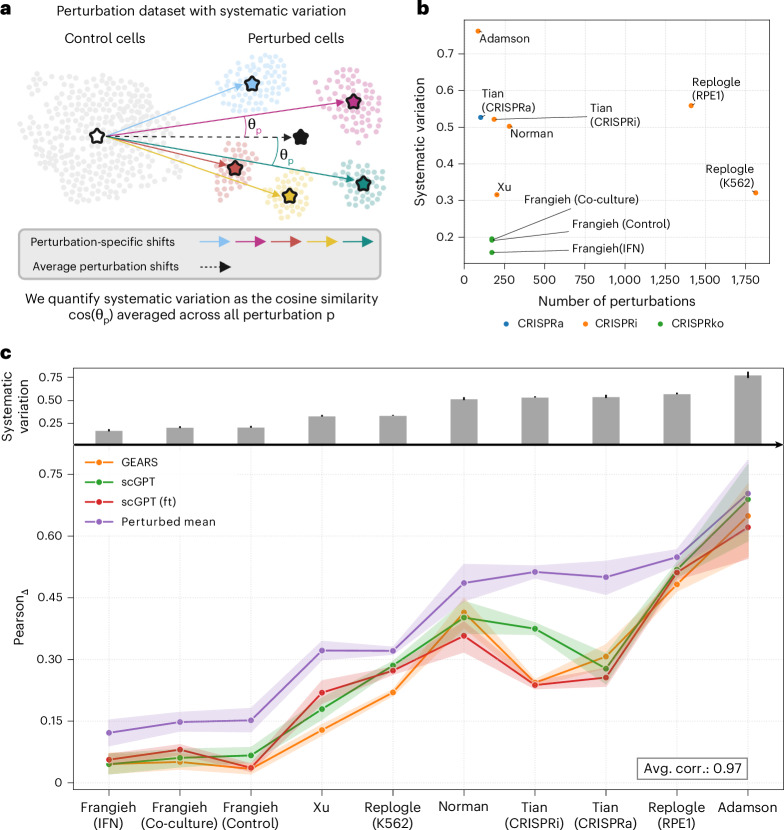
Quantifying systematic variation in single-cell perturbation datasets. **a**, Schematic representation of a single-cell perturbation dataset with systematic variation. We measure systematic variation as the cosine similarity between each perturbation-specific shift (vector pointing to the centroid of identically perturbed cells using the control centroid as reference) and the average perturbation effect (vector pointing to the centroid of all perturbed cells). Image created in BioRender. **b**, Degree of systematic variation in the datasets by total number of perturbations. For each dataset, we report the average cosine similarity between perturbation-specific shifts and the average perturbation effect. Datasets are colored by perturbation technology. **c**, Relationship between the performance of perturbation response prediction methods and systematic variation. The performance was measured using Pearson_*Δ*_ correlation on differential expression profiles on the one-gene test perturbations across ten datasets. Datasets are sorted by our measure of systematic variation. Error bars depict the 95% confidence interval of the scores across three independent runs with different data splits. The gray bars in the bar plot depict the degree of systematic variation in each dataset and error bars show the 95% confidence interval (Adamson: *n* = 81, Norman: *n* = 276, Replogle K562: *n* = 1,813, Replogle RPE1: *n* = 1,410, Tian CRISPRa: *n* = 97, Tian CRISPRi: *n* = 181, Xu: *n* = 198, Frangieh control: *n* = 167, Frangieh co-culture: *n* = 167, Frangieh interferon: *n* = 167; *n* is the total number of perturbations per dataset).

To quantify systematic variation, we computed the distribution of cosine similarities between perturbation-specific shifts and the average perturbation effect (Fig. 3a). We define perturbation-specific shifts as vectors that point to the centroid of cells that underwent the same type of perturbation using the centroid of control cells as reference, while the average perturbation effect is the vector pointing to the centroid of all perturbed cells. High cosine similarity indicates that the transcriptional responses to different perturbations are aligned in a similar direction, suggesting shared, possibly nonspecific, shifts in gene expression. We found that the degree of systematic variation differed considerably among the ten datasets considered in our benchmark (Fig. 3b). The widely used Adamson^13^ and Norman^14^ datasets exhibited high levels of systematic variation, consistent with their focused experimental designs targeting specific pathways. This suggests that predictive models trained on these datasets may be prone to selection bias, as they could exploit shared expression shifts that are consistent across all selected perturbations. Similarly, the Replogle^15^ RPE1 dataset showed more systematic variation than its K562 counterpart, consistent with our observation that perturbations in RPE1 cells induce more aligned transcriptional responses. In contrast, the three datasets from Frangieh et al.^18^ showed notably lower degrees of systematic variation, indicating that the directions of perturbation-specific shifts are more heterogeneous (as reflected by lower cosine similarities between individual perturbation-specific shifts and the average perturbation effect), which may reduce confounding in downstream analyses. We did not observe substantial differences in systematic variation when using different control types as reference (Supplementary Fig. 20).

We found that the amount of systematic variation in the perturbation datasets strongly correlated with the performance scores Pearson_*Δ*_ and Pearson_*Δ*20_ of existing perturbation response prediction methods (Fig. 3c and Supplementary Fig. 21). For example, the Pearson correlation between the average amount of systematic variation per dataset (mean cosine similarity) and Pearson_*Δ*_ was 0.91 for fine-tuned scGPT and 0.95 for GEARS. This suggests that these commonly used metrics can be influenced by systematic variation and may lead to inflated performance, as shown in Fig. 1c,d. Similarly, we observed monotonically decreasing Pearson_*Δ*_ and Pearson_*Δ*20_ correlation scores as we subsampled test perturbations based on how much systematic variation they captured (Supplementary Fig. 22). We did not observe the same trend using reference-insensitive metrics like RMSE or RMSE_20_ (the RMSE calculated on the top 20 differentially expressed genes of each perturbation; Supplementary Figs. 23 and 24). Reference-insensitive metrics like RMSE may offer more robust evaluation in the presence of systematic variation, as they do not rely on the control population as a reference point. Though harder to interpret, mean-squared error metrics have also been recommended by a recent study^24^ for evaluating gene expression prediction models. To test the impact of cell cycle on predictive scores, we downsampled perturbed cells from the Replogle^15^ RPE1 dataset to approximately match the phase distributions between perturbed and control cells (Supplementary Fig. 25). We found that this led to significantly lower Pearson_*Δ*_ and Pearson_*Δ*20_ scores (Supplementary Fig. 26). Overall, our results suggest that high predictive performance in terms of reference-based metrics (for example, Pearson_*Δ*_ and Pearson_*Δ*20_; Methods) may reflect dataset-specific confounding factors rather than accurate modeling of perturbation biology.

### Evaluating the prediction of perturbation-specific effects

**Fig. 4:**
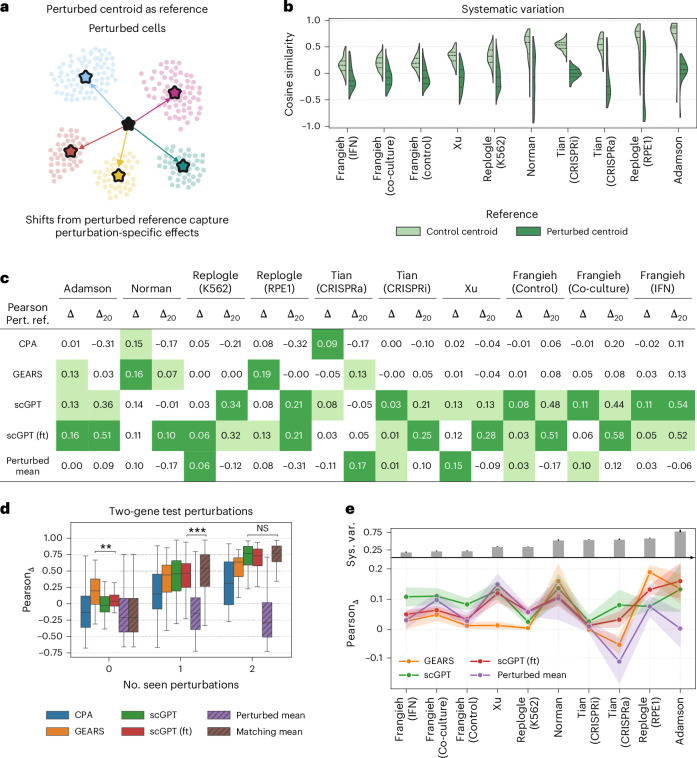
Systema emphasizes perturbation-specific effects and increases robustness to systematic variation. **a**, In the Systema framework, perturbation-specific effects are captured by changing the reference from the centroid of control cells to that of perturbed cells, thereby focusing on perturbation effects rather than systematic variation. Image created in BioRender. **b**, Distribution of cosine similarities between perturbation-specific shifts and the average perturbation-specific shift on ten single-cell perturbation datasets. Perturbation-specific shifts are computed using the control (light green) and perturbed (dark green) references. Dashed lines indicate distribution quartiles. **c**,**d**, Benchmarking perturbation response prediction using differential expression profiles with respect to the perturbed reference (Methods). Performance comparison of perturbed mean baseline and state-of-the-art methods on unseen one-gene perturbations (**c**). We report the average test performance across three independent runs with different data splits (*n* indicates number of test perturbations across all three independent runs, Adamson: *n* = 63, Norman: *n* = 108, Replogle K562: *n* = 1,362, Replogle RPE1: *n* = 1,059, Tian CRISPRa: *n* = 75, Tian CRISPRi: *n* = 138, Xu: *n* = 150, Frangieh control: *n* = 126, Frangieh co-culture: *n* = 126, Frangieh interferon: *n* = 126). Pearson correlation on unseen two-gene perturbations of the Norman^14^ dataset, applied to average differential expression profiles with respect to the perturbed reference (predicted versus ground truth) (**d**). Boxes depict distribution quartiles, with the center line corresponding to the median, and whiskers span 1.5 × interquartile range. Results across three independent runs with different train-test splits (0-seen: *n* = 39, 1-seen: *n* = 147, 2-seen: *n* = 39). We compared the two methods with highest median scores using two-sided paired *t*-tests performed separately for each independent run. The resulting *P* values were aggregated using Fisher’s method. 0-seen, *P* *=* 0.002; 1-seen, *P* *=* 0.0003; 2-seen, *P* *=* 0.6. Significance is denoted as NS, not significant (*P* > 0.05), *P ≤ 0.05, **P ≤ 0.01, ***P ≤ 0.001, ****P ≤ 0.0001. **e**, Relationship between the performance of perturbation response prediction methods and systematic variation. The performance was measured using Pearson_*Δ*_ correlation on differential expression profiles on the one-gene test perturbations across ten datasets, using the perturbed centroid as reference. Datasets are sorted by our measure of systematic variation. Error bars depict the 95% confidence interval of the scores across three independent runs with different data splits. The gray bars in the bar plot depict the degree of systematic variation in each dataset and error bars show the 95% confidence interval (Adamson: *n* = 81, Norman: *n* = 276, Replogle K562: *n* = 1,813, Replogle RPE1: *n* = 1,410, Tian CRISPRa: *n* = 97, Tian CRISPRi: *n* = 181, Xu: *n* = 198, Frangieh control: *n* = 167, Frangieh co-culture: *n* = 167, Frangieh interferon: *n* = 167; *n* is the total number of perturbations per dataset).

To increase robustness to systematic variation, we developed Systema, a new evaluation framework for perturbation response prediction. Instead of using control cells as a point of reference, Systema allows using custom references that better isolate perturbation-specific effects. As a key example, we propose shifting the reference to the average of perturbation-specific centroids, which we refer to as perturbed centroid (Fig. 4a and Methods). The perturbed centroid reference emphasizes perturbation-specific effects, which may facilitate evaluating the methods’ capabilities to distinguish the treatment effects of different perturbations from each other. We found that using the centroid of control cells as a reference, perturbation-specific shifts and the average shift (the shift corresponding to the average perturbation effect) pointed to similar directions (Fig. 4b and Methods), with an average cosine similarity $\cos (\theta )=0.41\pm 0.18$ across all datasets. For example, the average cosine similarity was $\cos (\theta )=0.76\pm 0.30$ for Adamson^13^, $\cos (\theta )=0.50\pm 0.26$ for Norman^14^ and $\cos (\theta )=0.32\pm 0.16$ for Replogle^15^ K562. In contrast, using the perturbed centroid as reference resulted in a distribution of cosine similarities centered nearly at zero, with an average cosine similarity of −0.06 across all datasets. For example, using the perturbed centroid as reference, the average cosine similarity was $\cos (\theta )=0.05\pm 0.23$ for Adamson^13^ and $\cos (\theta )=-0.12\pm 0.45$ for Norman^14^. Changing the reference had a weak impact on the norms and biological interpretation of the perturbation-specific shifts (Supplementary Figs. 27–32 and Supplementary Tables 3–6). We observed a similar pattern in terms of the distribution of pairwise cosine similarities (Supplementary Fig. 33) and results were robust to the panel of genetic perturbations (Supplementary Fig. 34). Using the perturbed centroid as reference is expected to reduce the overall impact of systematic variation on downstream analyses.

We then redefined standard evaluation metrics using the perturbed centroid as reference (Methods). Systema’s evaluation framework is more resilient to systematic variation because it allows us to focus on perturbation-specific effects. Applying Systema resulted in substantially lower evaluation scores (Fig. 4c–d and Supplementary Fig. 35). Contrary to previous results (Fig. 1c), all baselines attained low correlation scores at the task of generalizing to unseen one-gene perturbations (Fig. 4c). Using the redefined metrics, scGPT achieved marginally higher scores than the other baselines across the board. In terms of the combinatorial perturbations in Norman^14^, GEARS and scGPT were the only methods to achieve non-negative Pearson_*Δ*_ scores in the 0-seen perturbation setting (Fig. 4d and Supplementary Fig. 35), highlighting potential benefits of their inductive biases. For 1-seen and 2-seen combinatorial perturbations, GEARS, scGPT and the matching mean baseline achieved the best performance. Using the perturbed centroid as reference led to Pearson_*Δ*_ scores that had low correlation with the degree of systematic variation across all datasets (Fig. 4e) (the correlation with the Pearson_*Δ*_ metric averaged across methods was 0.21). In general, evaluation scores with this reference were substantially lower than those obtained with standard metrics that use the centroid of control cells as reference, indicating that predicting perturbation-specific effects for unseen genetic perturbations is a remarkably challenging task.

### Dissecting the predictable perturbations of perturbation response prediction methods

Existing perturbation response methods struggle to infer the perturbation-specific effects of unseen genetic perturbations. Can these methods still produce biologically informative predictions? To what extent can they uncover coarse-grained effects?

**Fig. 5:**
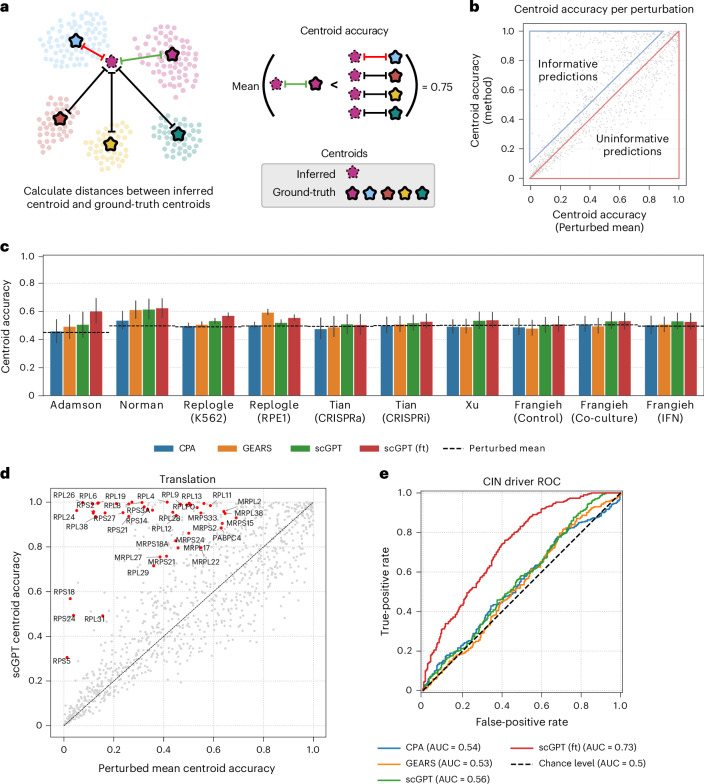
Systema queries predicted post-perturbation profiles to infer downstream, coarse-grained perturbation effects. **a**, Systema incorporates a novel metric, centroid accuracy, to evaluate whether the predicted post-perturbation profiles are closer to their ground-truth centroids than to the centroids of other perturbations. Image created in BioRender. **b**, Contrasting the centroid accuracies of the perturbed mean baseline (*x* axis) and a specific baseline (*y* axis). Each dot represents a perturbation. Perturbations in the diagonal are uninformative (they can be equally well predicted by the perturbation model and the perturbed mean), which captures systematic variation exclusively. Image created in BioRender. **c**, Comparing the centroid accuracy on unseen one-gene perturbations. We report the average test performance across three independent runs with different data splits (*n* indicates number of unique test perturbations across all three independent runs, Adamson: *n* = 49, Norman: *n* = 85, Replogle K562: *n* = 1,043, Replogle RPE1: *n* = 806, Tian CRISPRa: *n* 61, Tian CRISPRi: *n* = 107, Xu: *n* = 116, Frangieh control: *n* = 99, Frangieh co-culture: *n* = 99, Frangieh interferon: *n* = 99). Error bars depict the 95% confidence interval of the scores across three independent runs with different data splits. **d**, Contrasting the centroid accuracies of the perturbed mean baseline (*x* axis) and fine-tuned scGPT (*y* axis) on the Replogle^15^ (K562) dataset. Perturbations related to translation (for example, perturbations that target genes coding for ribosomal proteins) can be better inferred by scGPT. **e**, ROC curve for CIN prediction on the Replogle^15^ (K562) dataset. We split perturbations into low CIN (*z*-score < 0) and high CIN (*z*-score > 2) and computed centroids for the two classes. For each perturbation, we then calculated distances from the inferred centroid to the class-specific centroids and ranked perturbations based on the difference between the two distances (Methods). Only the fine-tuned version of scGPT could partially recapitulate CIN effects for held-out perturbations.

To investigate this, we introduced an intuitive evaluation metric in the Systema framework that we refer to as centroid accuracy, which measures whether predicted post-perturbation profiles are closer to their correct ground-truth centroid than to the centroids of other perturbations (Fig. 5a and Methods). A centroid accuracy of 1 indicates that the inferred profiles recover the expected transcriptional effects of a perturbation. Moreover, comparing the centroid accuracies of each perturbed gene to those of the perturbed mean can shed light on whether models capture perturbation biology beyond systematic effects (Fig. 5b). We applied this metric to evaluate predictions on unseen one-gene perturbations across ten datasets and found that the average perturbation scores barely exceeded those of the perturbed mean (Fig. 5c). Nonetheless, the fine-tuned version of scGPT achieved the highest accuracies overall, outperforming other methods in Adamson^13^, Norman^14^, and Replogle^15^ (K562) datasets. In the genome-wide Replogle^15^ K562 perturbation screen, contrasting the centroid accuracies of scGPT versus those of the perturbed mean revealed that scGPT was especially accurate in inferring the effects of perturbing genes involved in core cellular processes like gene expression, translation, and DNA replication (Fig. 5d and Supplementary Fig. 36). This included highly conserved and co-regulated gene families such as ribosomal protein-coding genes, likely due to the strong and stereotyped transcriptional programs induced by disrupting the translation machinery. The scGPT pretraining strategy on large-scale single-cell atlases may have endowed the model with biologically meaningful priors that facilitated generalization to unseen perturbations targeting functionally coherent gene groups.

To further evaluate the biological utility of perturbation response predictions, we extended the centroid accuracy to test whether the predicted centroids could distinguish coarse-grained perturbation effects (Methods). Specifically, we used the inferred centroids to classify unseen perturbations as inducing either low or high chromosomal instability (CIN) in the genome-wide K562 perturbation screen, using annotations from Replogle et al.^15^. We classified perturbations based on the distances between their inferred centroids and two class-specific centroids, representing perturbations inducing low and high chromosomal instabilities (Methods). Among all methods, only the fine-tuned version of scGPT achieved a receiver operating characteristic (ROC) area under the curve (AUC) substantially above chance (AUC 0.73; Fig. 5e), indicating that the model could partially recapitulate CIN-associated effects for held-out perturbations. Similarly, scGPT attained relatively high ROC AUC scores in distinguishing between perturbations targeting cytoplasmic versus mitochondrial protein-coding ribosomal genes, as well as perturbations of genes involved in messenger RNA processing (Supplementary Fig. 37). Together, these results demonstrate that Systema can aid in distinguishing between predictions that merely recapitulate systematic effects from those recovering biologically informative, coarse-grained perturbation responses.

## Discussion

In this work, we evaluated the capabilities of existing perturbation response prediction methods and found that their high predictive scores are largely driven by systematic biases. Moreover, we observed that systematic variation can profoundly affect downstream tasks and that existing reference-based metrics are susceptible to systematic effects, which may impair the accurate assessment of perturbation response prediction methods. To isolate perturbation-specific effects, we introduced Systema, a flexible evaluation framework that enables alternative points of reference, including the centroid of perturbed cells. Using this strategy, we observed substantially lower evaluation scores, demonstrating that generalizing to unseen genetic perturbations is a notoriously challenging task. While changing the reference enables highlighting perturbation-specific effects, this approach inherits the limitations of reference-sensitive metrics such as invariance to perturbation strength, sensitivity to the reference choice, and limited effectiveness in evaluating weak perturbations (Methods). Systema further includes an intuitive metric, the centroid accuracy, to study whether predicted perturbation profiles are biologically meaningful and amenable to downstream tasks. Using this strategy, we found that fine-tuned versions of large-scale pre-trained models such as scGPT can to some extent recover the effects of perturbations targeting functionally coherent groups of genes.

Looking forward, we believe that larger datasets with highly heterogenous perturbation gene panels will be essential to improve the robustness and generalizability of perturbation response models; however, as highlighted in this study, systematic variation may persist even in large-scale perturbation screens, such as Replogle^15^ RPE1, where perturbed cells showed marked shifts in cell-cycle occupancy relative to controls. Ultimately, we believe that perturbation response models should be evaluated based on their biological utility (how can inferred perturbation profiles help us answer downstream queries about relevant cellular phenotypes?). Framing evaluation in terms of downstream tasks may offer a more meaningful and practical perspective. In this light, emerging perturbation platforms like optical pooled screens^25,26^ and spatial functional genomics screens^27^, which combine perturbation data with cell morphology, spatial context and tissue-level features, present particularly rich opportunities. These modalities may offer access to a broad range of cellular and multicellular phenotypes, allowing predicted gene expression profiles to be studied not as the ultimate goal, but as an intermediate step toward understanding the functional impact of perturbations.

## Methods

### Nonparametric baselines

We design two nonparametric baselines that we refer to as perturbed mean and matching mean.

#### Perturbed mean

The perturbed mean generates a unique post-perturbation profile as the average gene expression of all perturbed cells in the train set. Let ${{\mathcal{P}}}_{{\rm{train}}}$ be the set of all perturbed cells in the train set and let ***x***_*i*_ be the gene expression of cell *i*. We compute the perturbed mean *μ*_pert_ as follows:

$${\mu }_{{\rm{pert}}}=\frac{1}{| {{\mathcal{P}}}_{{\rm{train}}}| }\sum _{i\in {{\mathcal{P}}}_{{\rm{train}}}}{{\boldsymbol{x}}}_{i}.$$

This profile is then used as a prediction for all genetic perturbations.

#### Matching mean

The matching mean is an extension of the perturbed mean for X+Y combinatorial perturbations. It computes post-perturbation profiles as the average of two gene expression vectors $\mu (\,\mathrm{X}\,)\in {{\mathbb{R}}}^{n}$ and $\mu (\,\mathrm{Y}\,)\in {{\mathbb{R}}}^{n}$, where *n* is the number of genes. Let ${{\mathcal{P}}}_{{\rm{train}}}$ be the set of all train perturbations and let ${\mathcal{P}}(\,\mathrm{X}\,)$ be the set of cells in which gene X (or combination of genes X) was perturbed. If the single-gene perturbation X was seen at train time (X $\in {{\mathcal{P}}}_{{\rm{train}}}$), *μ*(X) corresponds to the average profile of all X-perturbed cells in the train set. Otherwise, *μ*(X) is equivalent to the perturbed mean *μ*_pert_. Similarly, if perturbation Y was seen at train time, (Y $\in {{\mathcal{P}}}_{{\rm{train}}}$), *μ*(Y) corresponds to the average perturbation of all Y-perturbed cells in the train set. Otherwise, *μ*(Y) is equivalent to the perturbed mean *μ*_pert_. Mathematically, we compute the matching mean *μ*_matching_(X, Y) as follows:

$$\begin{array}{l}{\mu }_{{\rm{matching}}}(\,\mathrm{X},\mathrm{Y}\,)=\displaystyle\frac{1}{2}\left(\;\mu (\,\mathrm{X})+\mu (\mathrm{Y}\,)\right),\\\qquad\qquad \mu (\,\mathrm{X}\,)=\left\{\begin{array}{ll}\displaystyle\frac{1}{| {\mathcal{P}}(\,\mathrm{X}\,)| }{\sum }_{i\in {\mathcal{P}}(\mathrm{X})}{{\boldsymbol{x}}}_{i}\quad &\,\mathrm{if}\,\,X\,\in {{\mathcal{P}}}_{{\rm{train}}}\\ {\mu }_{{\rm{pert}}}\quad &\,\mathrm{if}\,\,X\,\notin {{\mathcal{P}}}_{{\rm{train}}}.\end{array}\right.\end{array}$$

For single-gene perturbations and combinatorial perturbations where none of the individual genes was seen at train time, the matching mean and the perturbed mean are equivalent.

### Perturbation response prediction methods

We consider three perturbation response prediction methods: CPA^10^, GEARS^1^ and scGPT^2^ (details in Supplementary Information A).

#### Note about CPA

CPA was designed for predicting transcriptional responses at the single-cell level for unseen dosages and cell types. This differs from the scenario considered in this paper (generalizing across unseen genetic perturbations). It is important to emphasize that CPA does not have mechanisms to predict transcriptional responses for unseen genetic perturbations, contrary to GEARS and scGPT. At test time, we used CPA to perform counterfactual inference by sampling random control cells as input and adding the effect of the desired perturbations in the latent space.

### Evaluation

#### Evaluation setting

We consider the evaluation setting of generalization to unseen genetic perturbations, including single and combinatorial perturbations. We follow the scenario and processing steps of GEARS^1^, which were also adopted by scGPT^2^. For all datasets, we split the data by perturbation, creating a test set consisting of 25% of the genetic perturbations that are never seen during training. In the combinatorial setting, test perturbations can be divided into three groups of growing complexity: 0/2, 1/2 and 2/2 unseen genetic perturbations. We repeat this process three times using different random seeds for data splitting and model training. Thus, our results include predictions across three independent runs.

#### Calculating differential expression profiles

We refer to the average expression changes with respect to a population of reference cells as differential expression profiles or simply as expression changes. Mathematically, let ***x***_*i*_ be the gene expression of cell *i*. Let ${\mathcal{P}}(\,\mathrm{X}\,)$ be the set of cells in which gene X (or combination of genes X) was perturbed and let ${\mathcal{C}}$ be the set of reference cells (for example, control cells). We define the differential expression profile *Δ*_X_ of perturbation X as:

$${\Delta }_{{\rm{X}}}=\left(\frac{1}{| {\mathcal{P}}(\,\mathrm{X}\,)| }\sum _{i\in {\mathcal{P}}(\,\mathrm{X}\,)}{{\boldsymbol{x}}}_{i}\right)-\left(\frac{1}{| {\mathcal{C}}| }\sum _{i\in {\mathcal{C}}}{{\boldsymbol{x}}}_{i}\right)$$

This expression essentially computes the average transcriptional changes induced by a perturbation with respect to a population of reference cells. To estimate the average expression changes inferred by a perturbation response prediction method, we replace the first term by the average of predicted post-perturbation profiles:

$${\hat{\Delta }}_{{\rm{X}}}=\left(\frac{1}{| \hat{{\mathcal{P}}}(\,\mathrm{X}\,)| }\sum _{i\in \hat{{\mathcal{P}}}(\,\mathrm{X}\,)}{\hat{{\boldsymbol{x}}}}_{i}\right)-\left(\frac{1}{| {\mathcal{C}}| }\sum _{i\in {\mathcal{C}}}{{\boldsymbol{x}}}_{i}\right)$$

where $\hat{{\mathcal{P}}}(\,\mathrm{X}\,)$ is the set of cells for perturbation X generated by a certain method and ${\hat{{\boldsymbol{x}}}}_{i}$ is the predicted transcriptome of cell *i*.

Geometrically, differential expression profiles correspond to vectors in the high-dimensional gene expression space pointing to the centroid of identically perturbed cells using a specific population of cells as reference (for example, centroid of control cells). We refer to these vectors as perturbation-specific shifts.

#### Reference-based metrics

We refer to metrics that evaluate how well the expression changes ${\hat{\Delta }}_{{\rm{X}}}$ reflect the true expression changes *Δ*_X_ as reference-based metrics. In particular, following GEARS^1^ and scGPT^2^, we used the Pearson correlation coefficient to calculate the correlation between the actual *Δ*_X_ and predicted ${\hat{\Delta }}_{{\rm{X}}}$ expression changes for all held-out perturbations, using (1) all genes (Pearson_*Δ*_) and (2) the top 20 differentially expressed genes of each perturbation (Pearson_*Δ*20_). For a given perturbation X, the Pearson_*Δ*20_ scores are calculated after selecting the top 20 differentially expressed genes induced by that perturbation from both the ground truth *Δ*_X_ and inferred ${\hat{\Delta }}_{{\rm{X}}}$ expression changes. Our results in Figs. 1 and 4 report the average of these scores across all held-out perturbations.

#### Reference-insensitive metrics

We refer to metrics that are not affected by the reference choice as reference-insensitive metrics. For example, the mean-squared error (MSE) is a reference-insensitive metric because the quantity MSE (${\Delta }_{{\rm{X}}},{\hat{\Delta }}_{{\rm{X}}}$) does not depend on the reference choice ${\mathcal{C}}$ (the reference cancels out). In other words, calculating the MSE on differential expression profiles *Δ*_X_ and ${\hat{\Delta }}_{{\rm{X}}}$ is equivalent to calculating MSE using the post-perturbation expression profiles. In our benchmark, we employ the RMSE and calculate it using (1) all genes (RMSE) and (2) the top 20 differentially expressed genes of each perturbation (RMSE_20_). The RMSE_20_ scores are calculated after selecting the top 20 differentially expressed genes from both the ground truth and inferred post-perturbation profiles of each perturbation.

#### Reference-based metrics resilient to systematic variation

In Systema, we consider the same set of evaluation metrics using the average O_pert_ of all perturbation-specific centroids O(X) as reference, which effectively emphasizes perturbation-specific effects. This centroid is computed as follows:

$${\mathrm{O}}_{{\rm{pert}}}=\frac{1}{| {{\mathcal{P}}}_{{\rm{train}}}| }\sum _{{\rm{X}}\in {{\mathcal{P}}}_{{\rm{train}}}}\,\mathrm{O}(\mathrm{X}\,),\qquad \,\mathrm{O}(\mathrm{X}\,)=\frac{1}{| {\mathcal{P}}(\,\mathrm{X}\,)| }\sum _{i\in {\mathcal{P}}(\,\mathrm{X}\,)}{{\boldsymbol{x}}}_{i}$$

where ${{\mathcal{P}}}_{{\rm{train}}}$ is the set of all train perturbations, O(X) is the centroid of perturbation X and ${\mathcal{P}}(\,\mathrm{X}\,)$ is the set of cells in which gene X (or combination of genes X) was perturbed. Our evaluation framework allows for different reference choices, including the origin, which leads to absolute post-perturbation profiles, and the average of perturbed cells.

#### Limitations of reference-sensitive metrics

Changing the reference to the perturbed centroid allows one to highlight perturbation-specific effects, but reference-sensitive metrics (for example, cosine similarity or Pearson correlation between perturbation-specific shifts) present some limitations. First, they might be inappropriate for evaluating weak perturbations because the norm of their shifts (with respect to the reference) is small, leading to noisy targets. That is, for weak perturbations, the presence or absence of a few single cells might have a large impact on the orientation of perturbation-specific shifts. Reference-insensitive metrics (for example, MSE) do not suffer from this limitation and have been recommended by recent studies^24^, but are comparatively harder to interpret. Second, reference-sensitive metrics are often invariant to perturbation strength. For example, the cosine similarity between two vectors does not depend on their norms. These metrics can thus be used to study whether methods can correctly predict the orientation of perturbation-specific shifts with respect to the reference, but not their magnitude. Finally, the reference choice can strongly influence evaluation scores and certain references may lead to undefined scores. For example, the Pearson correlation is undefined when perturbation-specific centroids overlap with the reference. In summary, reference-sensitive metrics are useful to study the relative orientations of perturbation-specific shifts and in this work we used them to investigate the extent to which perturbation response prediction methods capture systematic effects.

#### Centroid accuracy

To understand whether the inferred perturbation-specific centroids can accurately reconstruct the landscape of perturbations, in Systema we define a metric that we refer to as centroid accuracy. Intuitively, the centroid accuracy measures whether predicted post-perturbation profiles are closer to their correct ground-truth centroid than to the centroids of other perturbations.

Let O(X) be the centroid of perturbation X using the ground-truth expression and O_pred_(X) be the predicted centroid. Let ${\mathcal{P}}$ be the set of all perturbations. Mathematically, the centroid accuracy is defined as:

$$\begin{array}{l}{\rm{Centroid}}\,{\rm{accuracy}}(\,\mathrm{X}\,)\\=\displaystyle\frac{1}{| {\mathcal{P}}| -1}\sum _{{\rm{Y}}\in {\mathcal{P}}}{\mathbb{1}}\left[d\left({\mathrm{O}}_{{\rm{pred}}}(\,\mathrm{X}),\mathrm{O}(\mathrm{X}\,)\right) < d\left({\mathrm{O}}_{{\rm{pred}}}(\,\mathrm{X}),\mathrm{O}(\mathrm{Y}\,)\right)\right],\end{array}$$

where ${\mathbb{1}}$ is the indicator function and *d* is a predefined distance function (we used the Euclidean distance). We calculate the centroid accuracies of test perturbations. We can similarly measure the centroid accuracy with respect to a smaller set of coarse, class-specific centroids based on available perturbation annotations (for example, perturbations inducing strong vs weak chromosomal instabilities). To do so, we simply let ${\mathcal{P}}$ be the collection of class-specific centroids, O(X) the centroid corresponding to the class assigned to X, and O(Y) the centroid of class Y.

### Gene set enrichment analysis

To identify enriched pathways (between perturbed and control cells), we performed GSEA on the processed data using GSEApy^30^ and Biological Process 2023 gene sets from GO. We compared the populations of perturbed and control cells using signal-to-noise ratio as a ranking metric, defined as the difference of group means divided by the sum of standard deviations per group. We then ran GSEA^22^ with 500 random phenotype permutations (control versus perturbed) to calculate normalized enrichment scores and false discovery rates for each gene set.

### AUCell

We used AUCell^23^ to quantify the activity of pathways in single cells. AUCell calculates the enrichment of an input gene set as the AUC across the ranking of genes in a particular cell, leading to high scores if the gene set was enriched at the top of the ranking. This allowed us to measure whether certain gene sets were consistently enriched among perturbed or control cells.

### Dataset details

#### Data processing

We used the codebase from GEARS^1^ to process the data. We normalized each cell by total counts over all genes so that each cell has a total count equal to the median of total counts, followed by a log transformation. We discarded perturbations corresponding to genes not in the gene panel. This step is necessary for running scGPT (scGPT’s architecture was designed to handle perturbations of genes with available transcriptomic readouts). Following GEARS, we then selected the top 5,000 highly variable genes (HVGs) in each dataset^1^, and included the set of perturbed non-HVGs to the gene panel. For the Replogle^15^ datasets, we followed the data processing strategy of scGPT^2^: we retained the subset of data matching the 1,973 perturbations identified in the original study^15^ as inducing strong transcriptional changes, and then selected 100 cells per perturbation and 2,500 control cells. In terms of Replogle^15^ K562, we used the genome-wide K562 perturbation screen. In terms of the Tian^16^ data, we discarded the perturbations *PPP4R3A* (*SMEK1*; CRISPRa dataset) and *ATP5PD* (*ATP5H*; CRISPRi dataset) that targeted only a single cell. To process the Frangieh^18^ data, we followed the processing steps of Lopez et al.^31^ (we first converted the data from log-normalized count per millions into raw counts and removed cells with fewer than 500 expressed genes and genes expressed in fewer than 500 cells). We then selected cells perturbed with guides that exclusively target one gene. We finally normalized the data following the standard GEARS pipeline(normalize total counts, log transformation and selection of the top 5,000 HVGs plus perturbed non-HVGs) and partitioned cells into three datasets based on their condition (control, co-culture and interferon-γ). Figure 1b reflects the number of perturbations in each dataset after processing.

#### Control types

All datasets used non-targeting guides as controls and the Frangieh datasets^18^ further incorporated intergenic guides as additional controls.

#### Quantification of chromosomal instabilities

We downloaded the *z*-scored CIN annotations from Replogle et al.^15^. We categorized perturbations into two groups based on the extent to which they induced chromosomal instabilities: low CIN (*z*-score ≤ 0) and high CIN (*z*-score > 2).

#### Annotating cell cycle

To annotate cell cycle, we utilized the Scanpy^32^ function scanpy.tl.score_genes_cell_cycle and the cell-cycle signatures from Tirosh et al.^33^.

### Reporting summary

Further information on research design is available in the Nature Portfolio Reporting Summary linked to this article.

## Online content

Any methods, additional references, Nature Portfolio reporting summaries, source data, extended data, supplementary information, acknowledgements, peer review information; details of author contributions and competing interests; and statements of data and code availability are available at 10.1038/s41587-025-02777-8.

## Supplementary information

Supplementary Information Supplementary Figs. 1–37, Tables 1–6 and Notes A and B.

Reporting Summary

## Data Availability

We downloaded and processed data using the GEARS^1^ codebase. The Gene Expression Omnibus accession codes are Adamson^13^ (GSE90546), Norman^14^ (GSE146194) and Xu^17^ (GSE218566). The data from Replogle^15^ are available at figshare at 10.25452/figshare.plus.20022944 and additional annotations are available at 10.25452/figshare.plus.21632564. The Frangieh^18^ data are available at https://singlecell.broadinstitute.org/single_cell/study/SCP1064/multi-modal-pooled-perturb-cite-seq-screens-in-patient-models-define-novel-mechanisms-of-cancer-immune-evasion. The Tian^16^ data are available via scPerturb^6,34^ at 10.5281/zenodo.13350497 (ref. ^35^).

## Code availability

The source code is available on GitHub at https://github.com/mlbio-epfl/systema (ref. ^12^). The project website with links to data and code can be accessed at https://brbiclab.epfl.ch/projects/systema. We used the following Python packages: adjustText==1.0.4 anndata==0.9.2 blitzgsea==1.3.40 cell-gears==0.1.2 cpa-tools==0.8.8 gseapy==1.1.2 h5py==3.10.0 ipykernel==6.29.0 ipython==8.21.0 matplotlib==3.8.2 networkx==3.2.1 numpy==1.23.5 pandas==2.2.2 scanpy==1.9.8 scikit-learn==1.4.0 scipy==1.12.0 seaborn==0.13.2 statsmodels==0.14.1 torch==2.0.0 torch_geometric==2.5.1 tqdm==4.66.1 umap-learn==0.5.5 wandb==0.17.0 flash-attn==1.0.4 scgpt==0.2.1.
