# Supplementary material for: Systema: a framework for evaluating genetic perturbation response prediction beyond systematic variation
Source: Nat Biotechnol. 2025 Aug 25;44(6):1050–9. doi: 10.1038/s41587-025-02777-8 (PMC13271886; doi:10.1038/s41587-025-02777-8)
Supplement: Supplementary file 1 — Supplementary Figs. 1–37, Tables 1–6 and Notes A and B. [file 41587_2025_2777_MOESM1_ESM.pdf]

nature biotechnology

Supplementary information https://doi.org/10.1038/s41587-025-02777-8

Systema: a framework for evaluating genetic perturbation response prediction beyond systematic variation

In the format provided by the authors and unedited

Supplementary Information

A Perturbation response prediction methods

CPA The compositional Perturbational Autoencoder (CPA) framework Lotfollahi et al. (2023) is an autoencoder-based approach that can model single and combinatorial genetic perturbations as well as continuous perturbation covariates (*e.g.*, drug doses). For a given unperturbed cell i, CPA projects its transcriptome $\boldsymbol{x}_i \in \mathbb{R}^n$ into a low-dimensional vector $\boldsymbol{z}_i \in \mathbb{R}^k$, where n is the number of genes and k is an integer $k < n$. CPA decomposes the latent variation of the cell $\boldsymbol{z}_i = \bar{\boldsymbol{z}}_i + \delta_{p,d} + \epsilon_c$ as the sum of a basal state $\bar{\boldsymbol{z}}_i \in \mathbb{R}^k$ (i.e. unperturbed transcriptome), a shift $\delta_{p,d} \in \mathbb{R}^k$ induced by perturbation p with dose d, and variation $\epsilon_c \in \mathbb{R}^k$ related to observed covariates c (*e.g.*, cell type or technical information). To disentangle the latent variation driven by the perturbation and covariates from the basal state $\bar{\boldsymbol{z}}_i$, CPA utilizes an adversarial classifier trained to predict perturbation and covariate information from $\bar{\boldsymbol{z}}_i$. To encourage independence between the basal state $\bar{\boldsymbol{z}}_i$ and other variables, CPA incorporates an auxiliary loss term that penalizes the encoder when the adversary can recover exogenous information from the basal state $\bar{\boldsymbol{z}}_i$.

GEARS A limitation of CPA is that it was not designed generalize to unseen genetic perturbations. To address this problem, Roohani et al. (2023) presented Graph-Enhanced gene Activation and Repression Simulator (GEARS), a method that integrates prior knowledge from biological networks for genetic perturbation response prediction. GEARS first builds a knowledge graph of genes $\mathcal{G}_{\text{gene}}$ from co-expression networks and initializes the node features of each gene i with learnable node embeddings $\boldsymbol{g}_i \in \mathbb{R}^k$ of dimension k. Using data from the Gene Ontology (GO) database, GEARS builds another network of genetic perturbations $\mathcal{G}_{\text{pert}}$ by connecting functionally similar genes. For a given genetic perturbation i, this graph connects gene i with the genes sharing the highest fraction of pathways. The features of each perturbation node j are initialized with learnable node embeddings $\boldsymbol{p}_j \in \mathbb{R}^{k'}$ of dimension k'. GEARS utilizes two graph neural networks (GNN), respectively operating on the network of genes and network perturbations, to extract neighbour-aware embeddings for each gene $\hat{\boldsymbol{g}}_i = \text{GNN}(\boldsymbol{g}_i, \mathcal{G}_{\text{gene}})$ and perturbation $\hat{\boldsymbol{p}}_j = \text{GNN}(\boldsymbol{p}_j, \mathcal{G}_{\text{pert}})$. For a given gene i and set of genetic perturbations $\mathcal{P}$, GEARS decodes the perturbation effect from the post-perturbation embeddings $\boldsymbol{h} = \text{MLP}\big(\hat{\boldsymbol{g}}_i + \text{MLP}(\sum_{j\in\mathcal{P}} \hat{\boldsymbol{p}}_j)\big)$. The model is trained using an autofocus loss that gives higher weight to differentially expressed genes.

scGPT Cui et al. (2024) proposed a foundation model called scGPT for single-cell data, with perturbation response prediction being one of multiple downstream possible tasks. The model is pre-trained on a large corpus of single-cell data via masked token prediction. scGPT assigns each gene and condition (*e.g.*, genetic perturbation) a unique token of length k (*i.e.*, vector of learnable weights). After projecting the expression of each gene to a vector of length k, scGPT sums the encoded gene, condition, and expression tokens to obtain a matrix of embeddings $\boldsymbol{H}_i \in \mathbb{R}^{m\times k}$ for each cell i, where m is the number of genes. These embeddings $\boldsymbol{H}_i$ are updated iteratively using a transformer model and the expression of the masked genes is recovered from the transformer's output. For perturbation response, scGPT is fine-tuned to predict the expression of perturbed cells from random control cells, encoding genetic perturbation information via the condition tokens. For perturbation response prediction, scGPT is fine-tuned via regression (rather masked token prediction).

B Evaluation in other works

Evaluation

scGen, CPA, and chemCPA evaluation. scGen (Lotfollahi et al., 2019), CPA (Lotfollahi et al., 2023), and chemCPA (Hetzel et al., 2022) can generate counterfactual predictions for every single-cell. Yet, these methods are solely evaluated in terms of reconstruction performance and latent space disentanglement (Lotfollahi et al., 2019; 2023; Hetzel et al., 2022). In other words, evaluation metrics (R^2 and MSE) are computed between ground-truth post-perturbation expression single-cell profiles and their reconstructions as predicted by the autoencoder models. In conjunction, disentanglement metrics assess the extent to which perturbation-related information is removed from the latent space. We note that high-capacity autoencoders with imper-

fect latent space alignment may achieve close-to-perfect reconstruction performance and that these metrics should not be used as a proxy for counterfactual inference performance.

GEARS and scGPT evaluation. Both GEARS (Roohani et al., 2023) and scGPT (Cui et al., 2024) evaluate performance by comparing the average predicted and ground-truth perturbation changes via Pearson correlation. This strategy is preferred over comparing post-perturbation profiles because control (unperturbed) and perturbed cells often exhibit highly correlated profiles. Moreover, most perturbagens only affect a small set of genes (Yao et al., 2023), which may lead to noisy differential expression profiles when the entire transcriptome is considered. For this reason, GEARS and scGPT also evaluate the Pearson correlation between the predicted and ground-truth perturbation changes using the top 20 differentially expressed genes of each perturbation. GEARS also considered the mean squared error between predicted and true post-perturbation profiles, using the top 20 differentially expressed genes of each perturbation, and the Jaccard similarity between predicted and true differentially expressed genes.

C Supplementary Figures

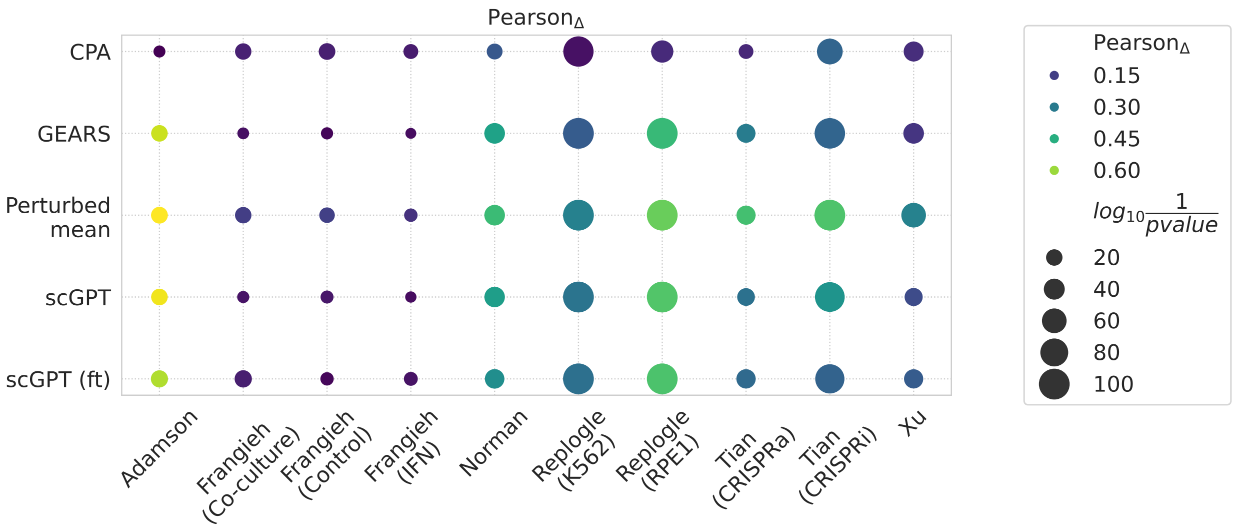

Supplementary Figure 1: Bubble plot showing the performance comparison of the perturbed mean baseline and state-of-the-art perturbation response prediction methods on unseen 1-gene perturbations. We evaluated performance on ten single-cell perturbation datasets from six different sources (Adamson et al., 2016; Norman et al., 2019; Replogle et al., 2022; Tian et al., 2019; Xu et al., 2024; Frangieh et al., 2021) using the Pearson$_\Delta$ metric. We report the average test performance across three independent runs with different data splits (n: number of test perturbations across all three independent runs, Adamson: $n = 63$, Norman: $n = 108$, Replogle K562: $n = 1362$, Replogle RPE1: $n = 1059$, Tian CRISPRa: $n = 75$, Tian CRISPRi: $n = 138$, Xu: $n = 150$, Frangieh control: $n = 126$, Frangieh co-culture: $n = 126$, Frangieh interferon: $n = 126$). Color intensities are proportional to predictive performance and dot sizes are proportional to $\log_{10}\left(\frac{1}{\text{p-value}}\right)$. We employed a two-sided one-sample t-test, performed separately for each indepedent run, to test whether the expected value of the test perturbation scores is significantly different from 0. The resulting p-values were aggregated using Fisher's method.

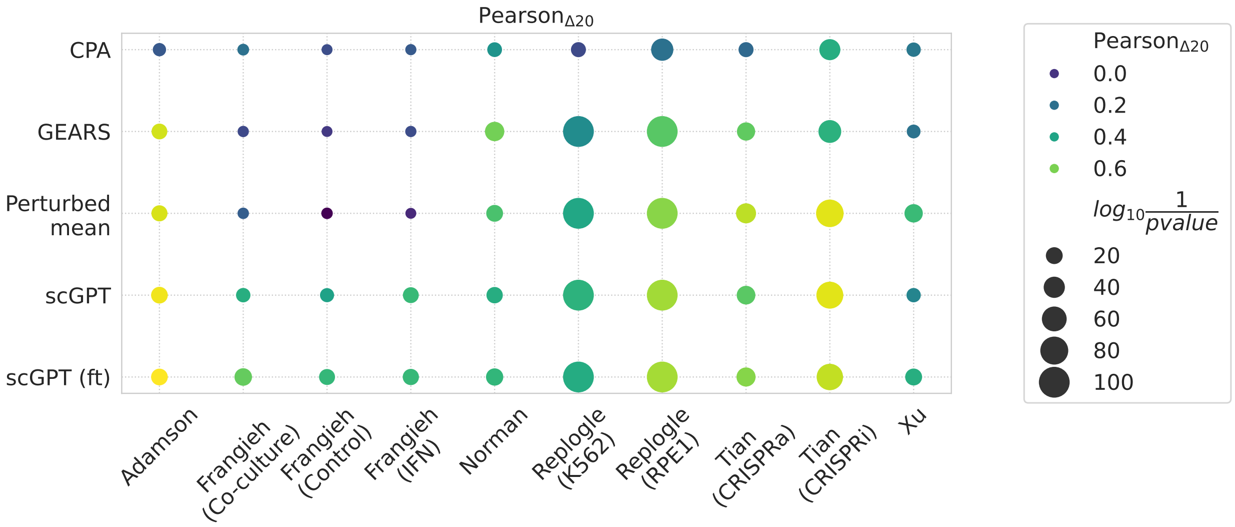

Supplementary Figure 2: Bubble plot showing the performance comparison of the perturbed mean baseline and state-of-the-art perturbation response prediction methods on unseen 1-gene perturbations. We evaluated performance on ten single-cell perturbation datasets from six different sources (Adamson et al., 2016; Norman et al., 2019; Replogle et al., 2022; Tian et al., 2019; Xu et al., 2024; Frangieh et al., 2021) using the Pearson$_\Delta$ metric after selecting the top 20 differentially expressed genes of each test perturbation. We report the average test performance across three independent runs with different data splits (n: number of test perturbations across all three independent runs, Adamson: $n = 63$, Norman: $n = 108$, Replogle K562: $n = 1362$, Replogle RPE1: $n = 1059$, Tian CRISPRa: $n = 75$, Tian CRISPRi: $n = 138$, Xu: $n = 150$, Frangieh control: $n = 126$, Frangieh co-culture: $n = 126$, Frangieh interferon: $n = 126$). Color intensities are proportional to predictive performance and dot sizes are proportional to $\log_{10}\left(\frac{1}{\text{p-value}}\right)$. We employed a two-sided one-sample t-test, performed separately for each indepedent run, to test whether the expected value of the test perturbation scores is significantly different from 0. The resulting p-values were aggregated using Fisher's method.

RMSE	Adamson		Norman		Replogle (K562)		Replogle (RPE1)		Tian (CRISPRa)		Tian (CRISPRi)		Xu		Frangieh (Control)		Frangieh (Co-culture)		Frangieh (IFN)	
	All	20	All	20	All	20	All	20	All	20	All	20	All	20	All	20	All	20	All	20
CPA	0.10	0.59	0.05	0.51	0.07	0.33	0.12	0.51	0.04	0.19	0.03	0.10	0.01	0.09	0.52	0.57	0.52	0.53	0.53	0.63
GEARS	0.07	0.38	0.05	0.45	0.07	0.31	0.11	0.39	0.05	0.15	0.04	0.10	0.01	0.09	0.05	0.16	0.04	0.16	0.05	0.17
scGPT	0.07	0.34	0.05	0.48	0.07	0.29	0.11	0.37	0.03	0.17	0.02	0.09	0.01	0.09	0.04	0.11	0.04	0.14	0.04	0.14
scGPT (ft)	0.08	0.30	0.06	0.47	0.07	0.29	0.11	0.36	0.04	0.16	0.03	0.09	0.01	0.08	0.05	0.11	0.05	0.14	0.05	0.14
Perturbed mean	0.06	0.38	0.05	0.47	0.06	0.31	0.10	0.41	0.03	0.14	0.02	0.09	0.01	0.08	0.04	0.13	0.04	0.16	0.04	0.16

Supplementary Figure 3: Performance comparison of perturbed mean baseline and state-of-the-art methods on unseen 1-gene perturbations. We evaluated performance on ten single-cell perturbation datasets from six different sources (Adamson et al., 2016; Norman et al., 2019; Replogle et al., 2022; Tian et al., 2019; Xu et al., 2024; Frangieh et al., 2021) using the root mean squared error (RMSE) on all genes (all) and after selecting the top 20 differentially expressed genes of each test perturbation. We report the average test performance across three independent runs with different data splits (n: number of test perturbations across all three independent runs, Adamson: $n = 63$, Norman: $n = 108$, Replogle K562: $n = 1362$, Replogle RPE1: $n = 1059$, Tian CRISPRa: $n = 75$, Tian CRISPRi: $n = 138$, Xu: $n = 150$, Frangieh control: $n = 126$, Frangieh co-culture: $n = 126$, Frangieh interferon: $n = 126$).

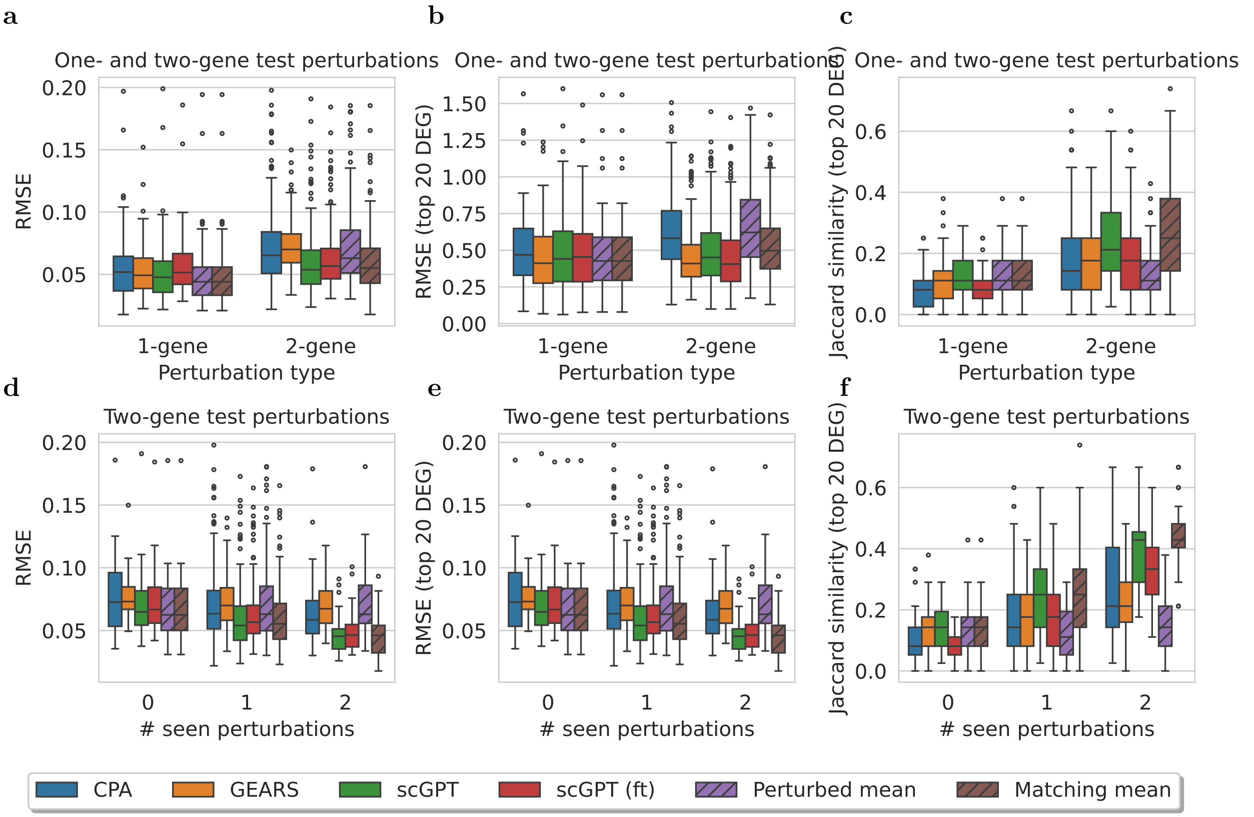

Supplementary Figure 4: Benchmarking perturbation response prediction on Norman et al. (2019) with additional metrics. We used the RMSE, $RMSE_{20}$ (calculated on the top 20 differentially expressed genes of each perturbation), and Jaccard similarity between the top 20 predicted differentially expressed genes and true differentially expressed genes. We report the average results across three independent runs with different data splits (n: number of test perturbations across all three independent runs, Norman: $n = 108$).(**a, b**) Root mean squared error (RMSE) for 1-gene and 2-gene test perturbations. (**d, e**) RMSE for 2-gene test perturbations by number of matching 1-gene perturbations observed at train time. (**a, b, d, e**) RMSE using (**a, d**) all genes and (**b, e**) the top 20 differentially expressed genes. (**c, f**) Jaccard similarity between the top 20 predicted differentially expressed genes and true differentially expressed genes. Overall, simple baselines (perturbed mean and matching mean) attained comparable or superior performance to existing perturbation response prediction methods across different metrics. Boxes depict distribution quartiles, with the center line corresponding to the median, and whiskers span 1.5 times the interquartile range.

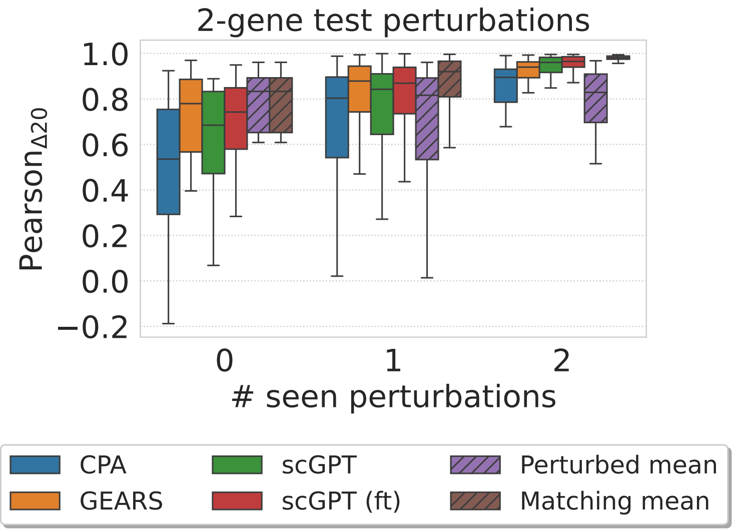

Supplementary Figure 5: Performance on 2-gene perturbations of the Norman et al. (2019) dataset, using the Pearson $_{\Delta 20}$ computed on differential expression profiles (n: number of test perturbations across all three independent runs, Norman: $n = 108$). Boxes depict distribution quartiles, with the center line corresponding to the median, and whiskers span 1.5 times the interquartile range.

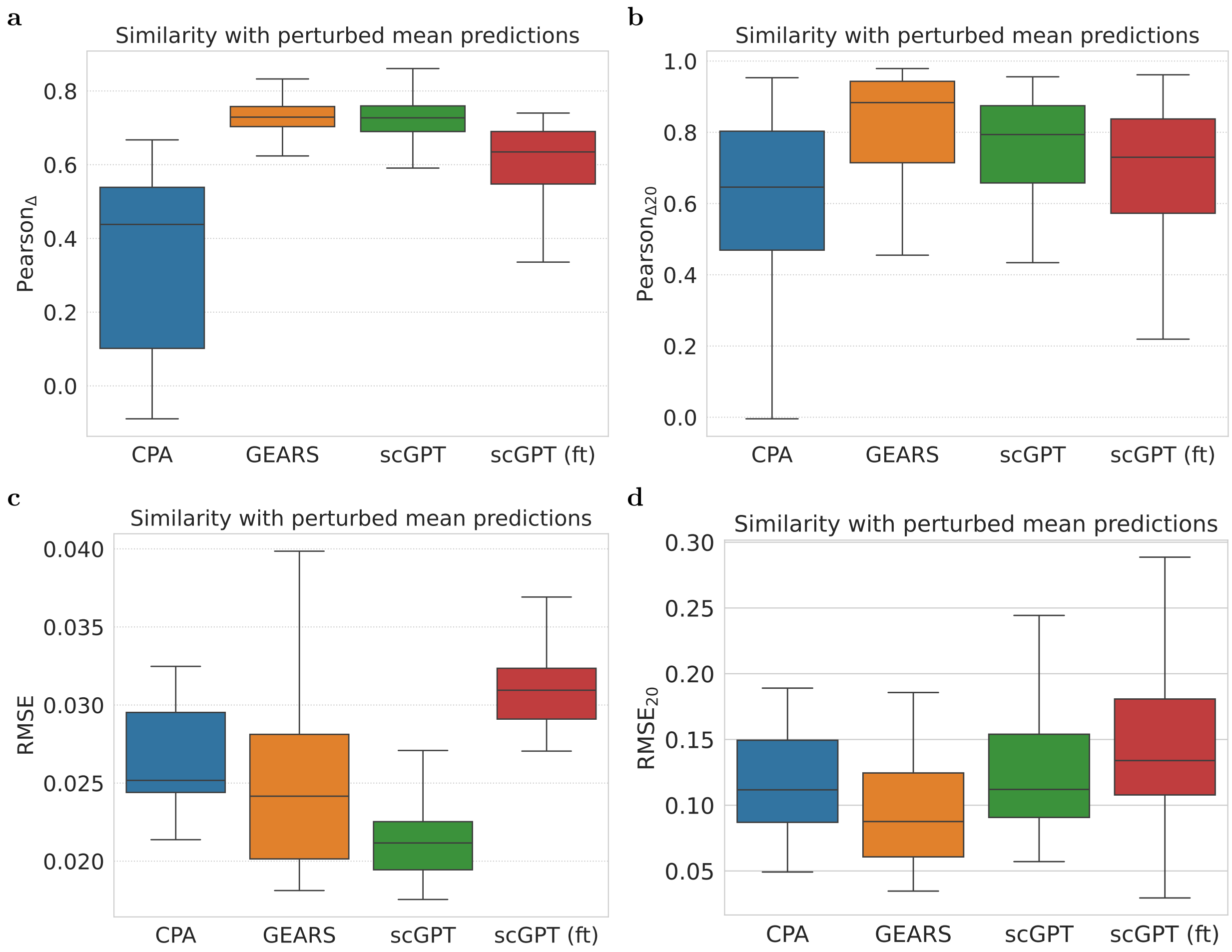

Supplementary Figure 6: Similarity of predictions with perturbed mean baseline on 1-gene test perturbations from the Norman et al. (2019) dataset. We compared the predicted differential expression profiles of each baseline with those of the perturbed mean (n: number of test perturbations across all three independent runs, Norman: $n = 108$). (**a**) Pearson$_\Delta$. (**b**) Pearson$_{\Delta 20}$. (**c**) RMSE. (**d**) RMSE$_{20}$. Results across three independent runs with different splits. Boxes depict distribution quartiles, with the center line corresponding to the median, and whiskers span 1.5 times the interquartile range.

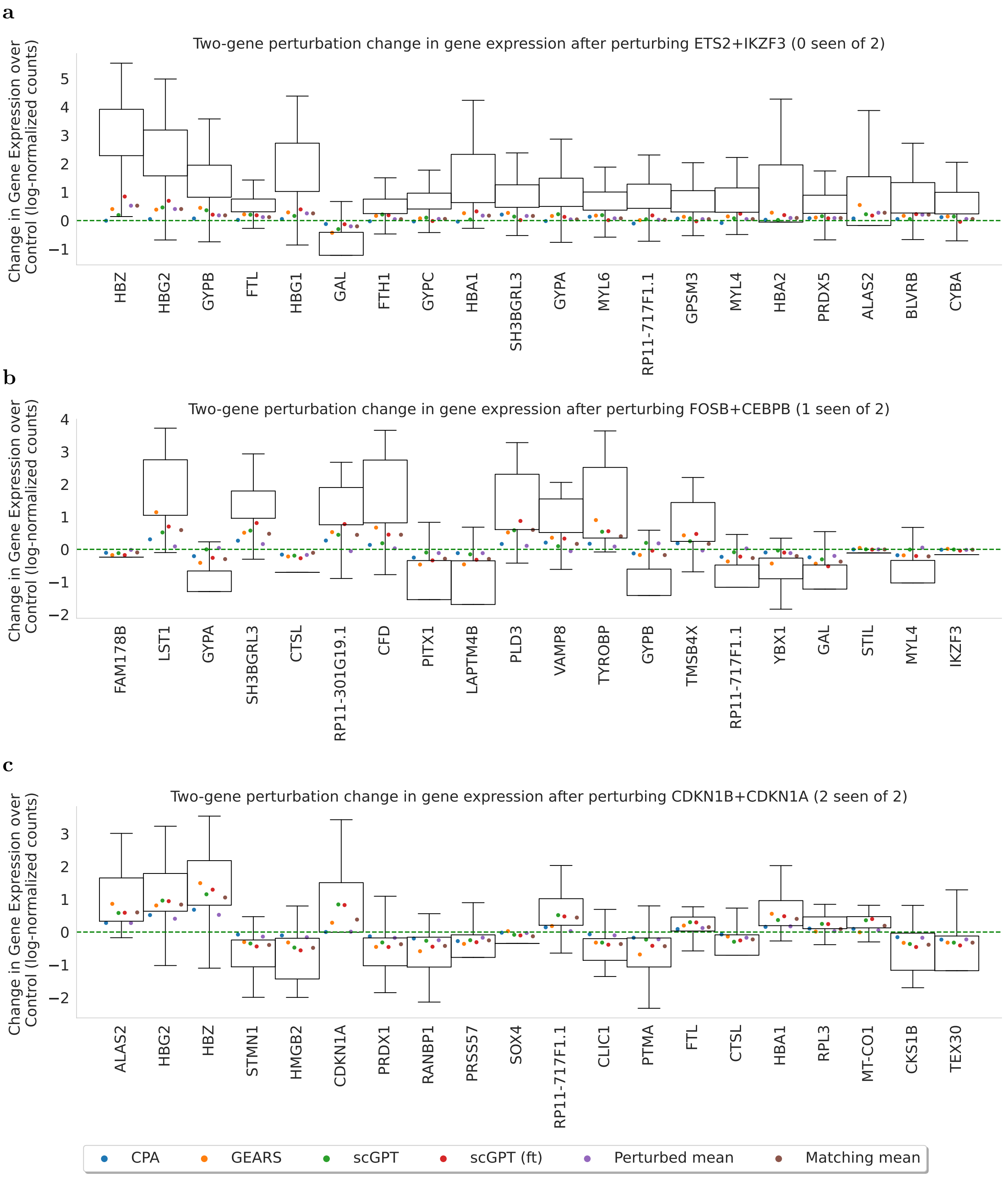

Supplementary Figure 7: Predicted change in expression for top 20 differentially expressed genes in three scenarios: (**a**) ETS2+IKZF3 (0 perturbations seen at train time), (**b**) FOSB + CEBPB (1 perturbation seen at train time), and (**c**) CDKN1B + CDKN1A (2 perturbations seen at train time).

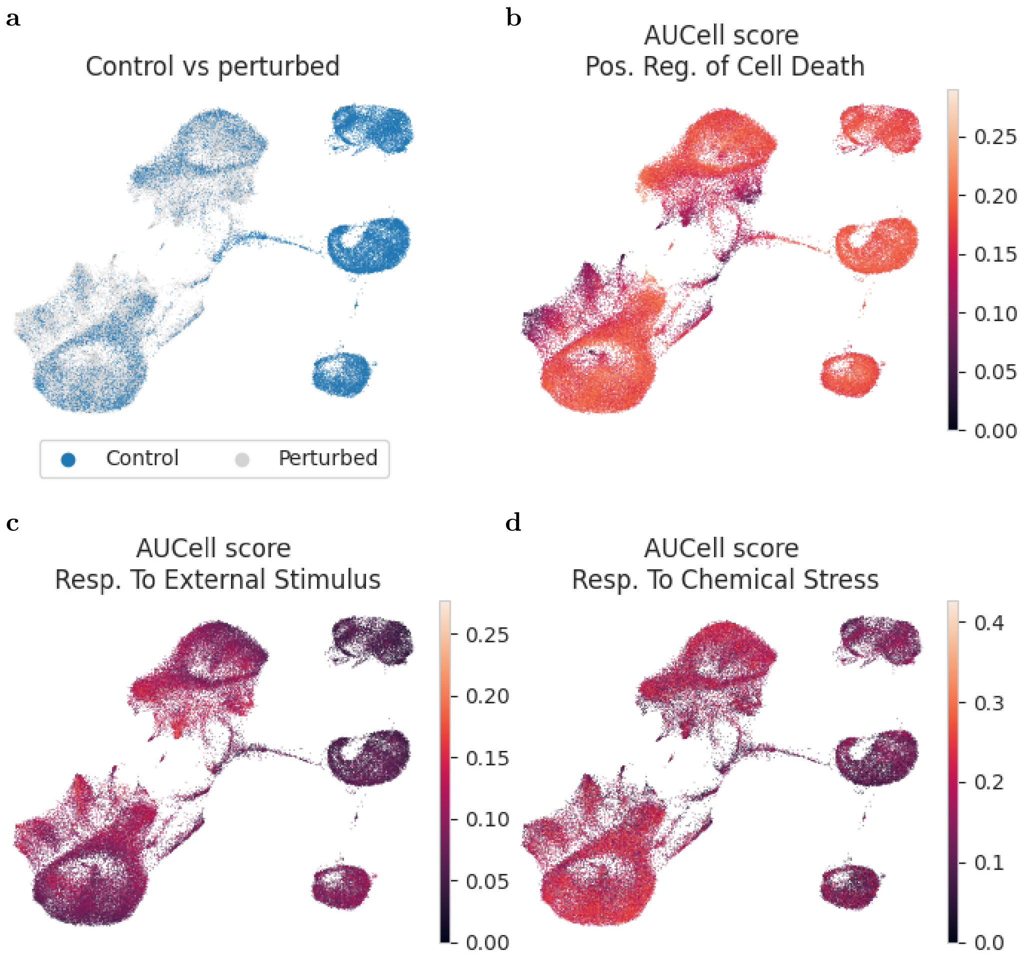

Supplementary Figure 8: Qualitative analysis of systematic differences in the Adamson et al. (2016) dataset highlighted by AUCell scores (Aibar et al., 2017). AUCell calculates gene set enrichments, providing an activity score for each cell. UMAP plot of normalized gene expression values colored by (**a**) control vs perturbed cells and AUCell scores of (**b**) positive regulation of cell death, (**c**) positive regulation of response to external stimulus, and (**d**) cellular response to chemical stress. Control cells correspond to cells with non-targeting guides.

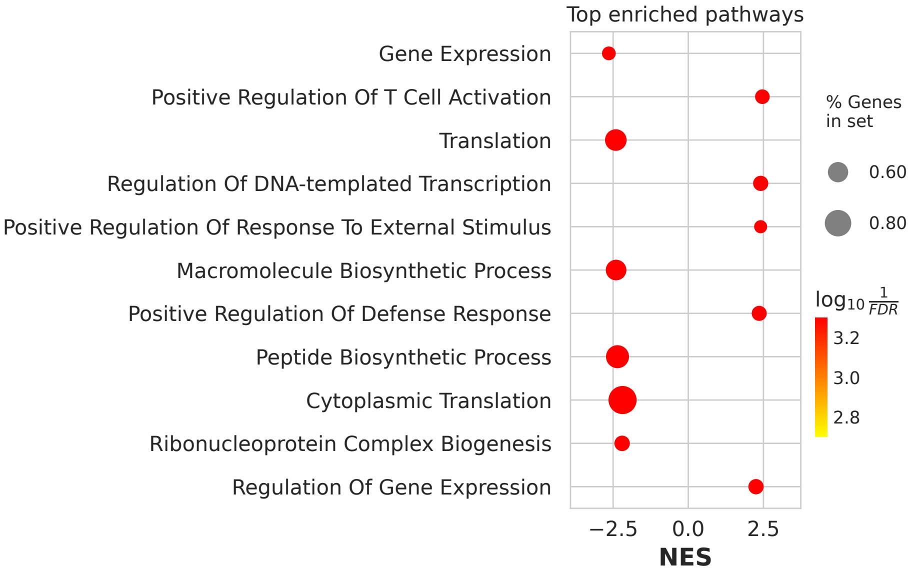

Supplementary Figure 9: Normalized enrichment scores and false discovery rate for top enriched pathways in the Adamson et al. (2016) dataset.

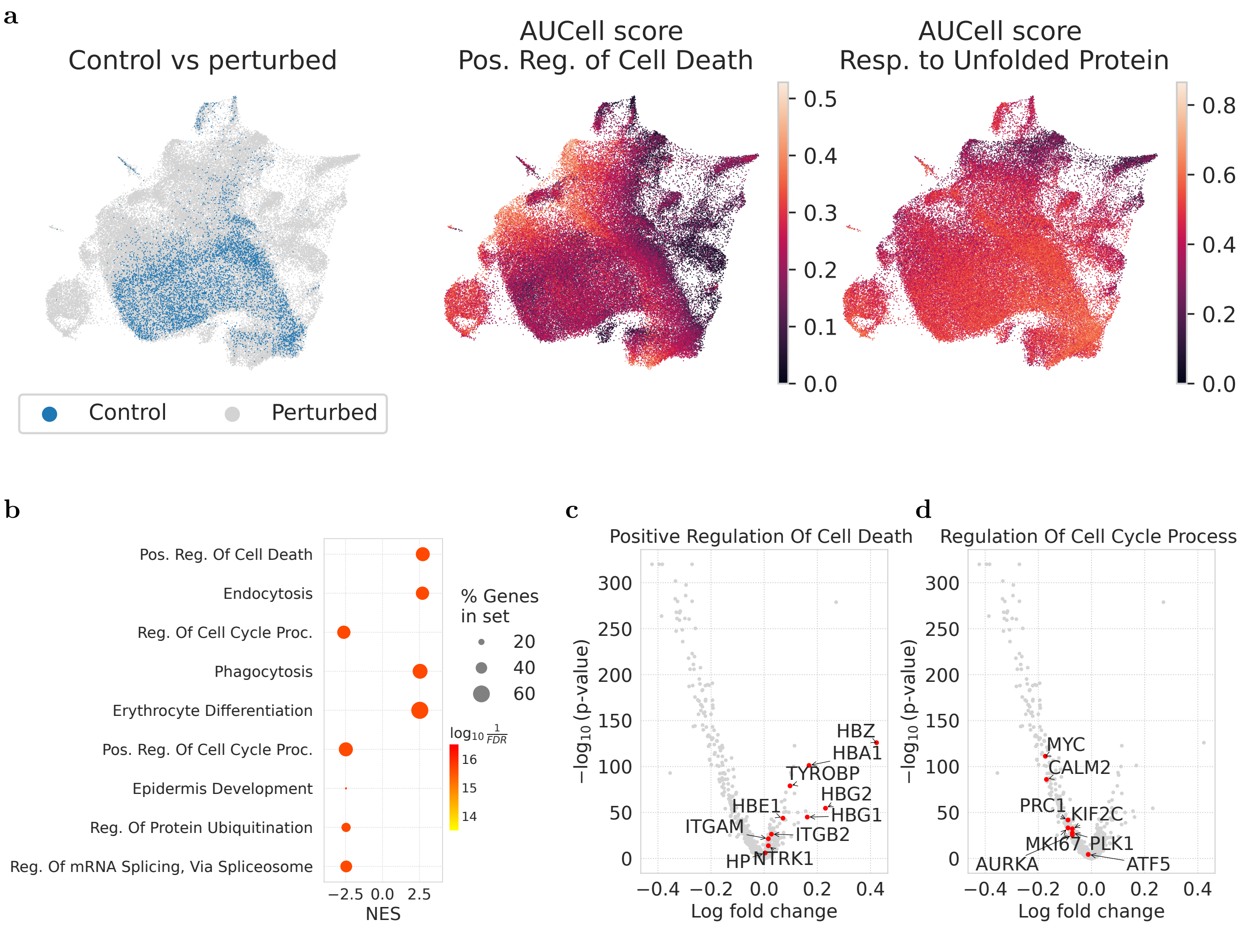

Supplementary Figure 10: **Systematic variation in Norman et al. (2019)**. (**a**) UMAP plot of normalized gene expression values colored by control vs perturbed cells, AUCell scores of positive regulation of cell death, and positive regulation of response to external stimulus and response to unfolded protein. Control cells correspond to cells with non-targeting guides. (**b**) Normalized enrichment scores and false discovery rate for top enriched pathways. We applied Gene Set Enrichment Analyses using *Biological Process 2023* gene sets from the Gene Ontology (GO) (Aleksander et al., 2023; Ashburner et al., 2000) to compare the population of perturbed cells with the population of control cells. (**c, d**) Volcano plot depicting the log-fold changes (perturbed vs control cells) for gene sets involved in (**c**) positive regulation of cell death and (**d**) regulation of cell cycle process. We used a two-sided independent two-sample t-test without adjustments. Control and perturbed cells exhibit systematic differences that can be easily captured by perturbation response prediction methods.

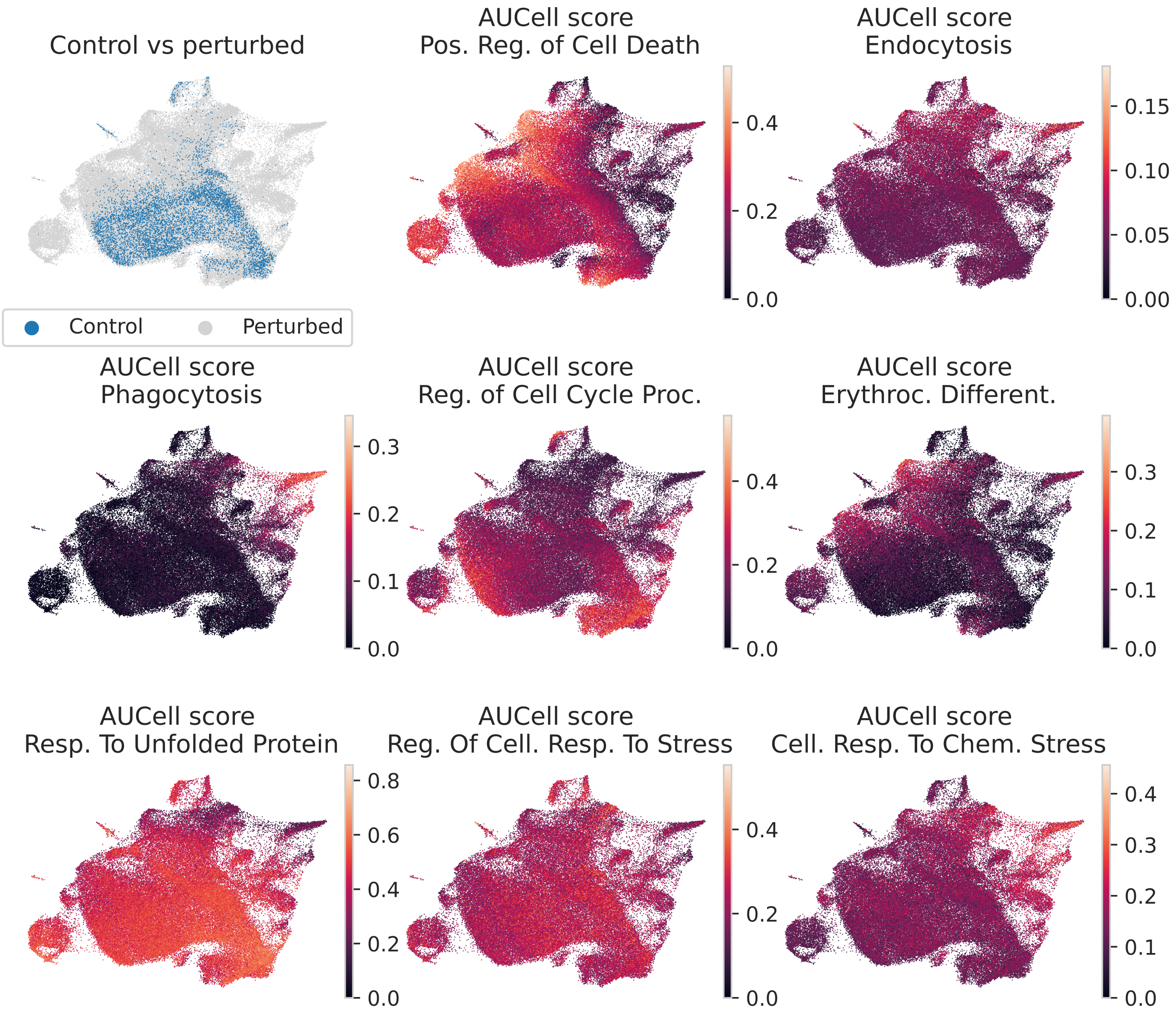

Supplementary Figure 11: Qualitative analysis of systematic differences in the Norman et al. (2019) dataset highlighted by AUCell scores (Aibar et al., 2017). AUCell calculates gene set enrichments, providing an activity score for each cell. UMAP plot of normalized gene expression values colored by control vs perturbed cells and AUCell scores of positive regulation of cell death, endocytosis, phagocytosis, regulation of cell cycle process, erythrocyte differentiation, response to unfolded protein, regulation of cellular response to stress, and cellular response to chemical stress. Control cells correspond to cells with non-targeting guides.

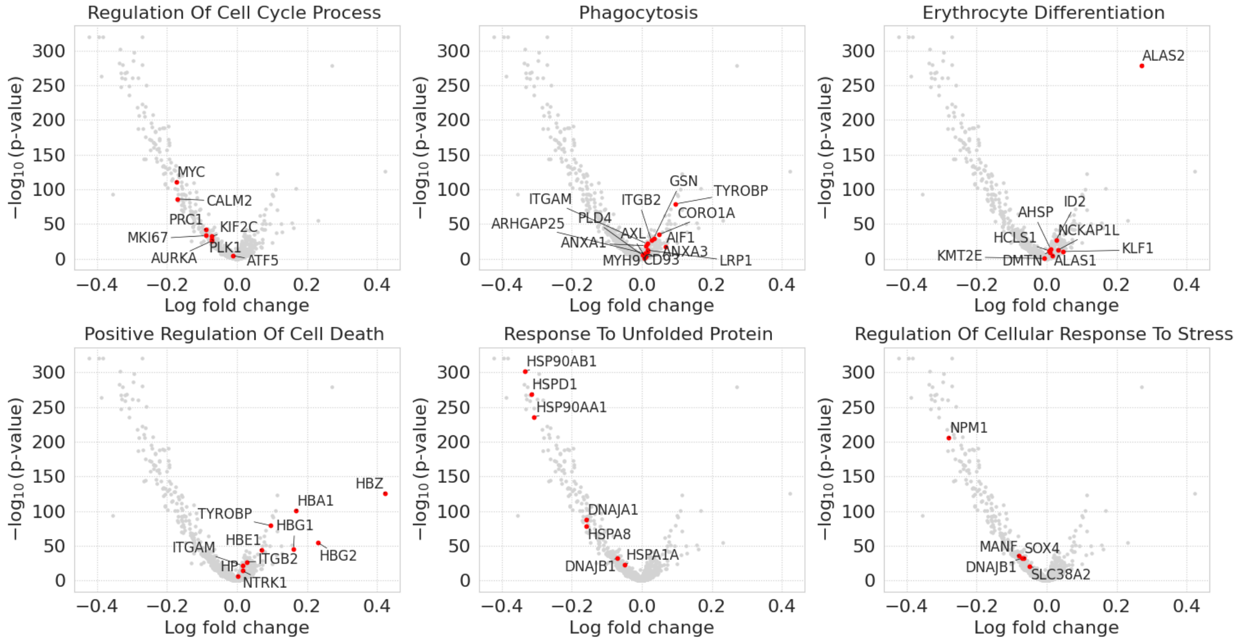

Supplementary Figure 12: Volcano plot depicting the log-fold changes (perturbed vs control cells) in Norman et al. (2019) for gene sets involved in regulation of cell cycle process, phagocytosis, erythrocyte differentiation, positive regulation of cell death, response to unfolded protein, and regulation of cellular response to stress. Norman et al. (2019) perturbed a panel of genes whose activation enhances or retards growth of K562 cells, including cell cycle regulators and genes that drive erythroid differentiation (Norman et al., 2019). We used a two-sided independent two-sample t-test without adjustments.

Supplementary Table 1: Lead genes that are differentially expressed consistently between perturbed and control cells in Norman et al. (2019) (two-sided t-test p-value < 0.05) for the top 10 enriched pathways.

**Pathway**	**Lead Genes**
Positive Regulation Of Cell Death	*NTRK1, ITGAM, TYROBP, HBG1, HBA1, HBE1, HBZ, HP, HBG2, ITGB2*
Endocytosis	*LRP12, RAB5B, LRP1, FOLR1, ITGAM, AXL, CD93, PLD4, PECAM1, DENND1C, IL10RA, ANXA3, ANXA1, APOE, DPYSL2, CORO1A, ITGB2*
Regulation Of Cell Cycle Process	*CALM2, ATF5, PLK1, MKI67, MYC, AURKA, PRC1, KIF2C*
Phagocytosis	*LRP1, ITGAM, TYROBP, CD93, AXL, PLD4, AIF1, ANXA3, ANXA1, GSN, CORO1A, ITGB2, ARHGAP25*
Erythrocyte Differentiation	*KLF1, HCLS1, DMTN, NCKAP1L, ALAS2, AHSP, ID2, ALAS1*
Positive Regulation Of Cell Cycle Process	*BIRC5, SMC4, NUSAP1, RANBP1, KIF14, AURKA, PLK4, RGCC, CDCA8*
Epidermis Development	*FABP5, YBX1*
Regulation Of Protein Ubiquitination	*HSPA1B, RPS3, DNAJA1, HSP90AA1, HSPA1A, SOX4, HSP90AB1, UBB*
Regulation Of mRNA Splicing, Via Spliceosome	*SRSF3, CELF2, SON, NCL*
Myeloid Cell Differentiation	*KLF1, RASGRP4, CEBPE, HCLS1, EVI2B, ALAS2, AHSP*

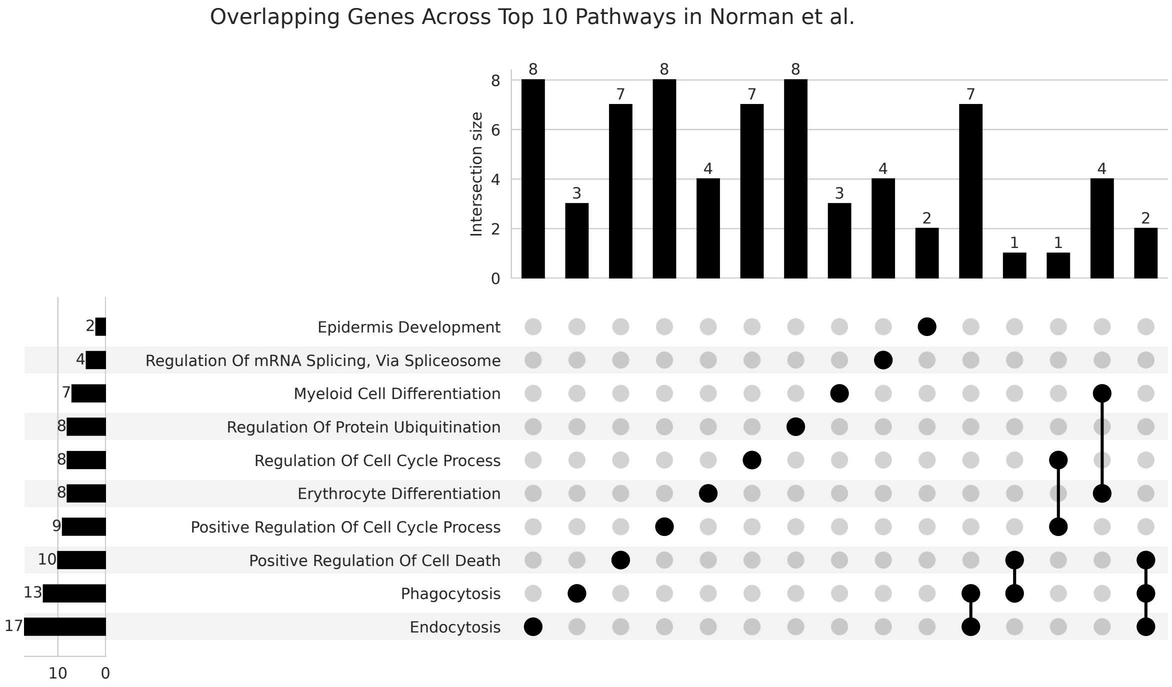

Supplementary Figure 13: Upset plot showing the number of differentially expressed (DE) genes (two-sided t-test p-value < 0.05) overlapping among the top 10 pathways in Norman et al. Left bars: number of DE genes per pathway.

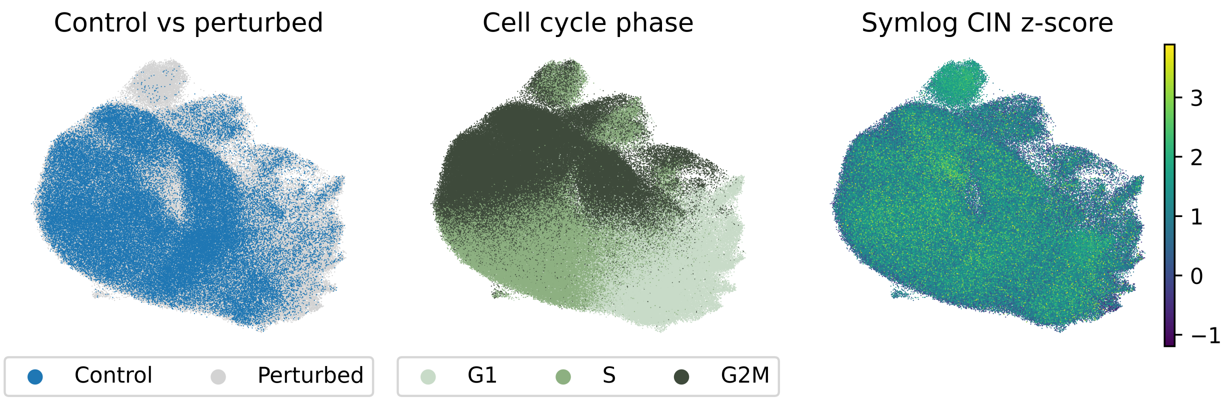

Supplementary Figure 14: UMAP plot of normalized gene expression values in the genome-wide Replogle K562 dataset colored by control vs perturbed cells (left), cell cycle phase (middle), and chromosomal instability (CIN) z-score of each perturbation (right) . The perturbation CIN score was calculated as the mean single-cell sum of squared CIN values, z-normalized relative to control perturbations (Replogle et al., 2022).

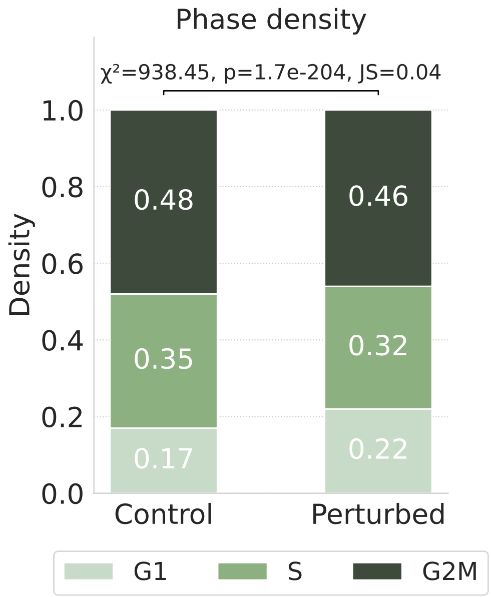

Supplementary Figure 15: Phase density of control and perturbed cells. The density of cells in G1 is significantly higher for perturbed cells with p-value=1.7e-204 (χ^2: Chi2 test statistic, JS: Jensen-Shannon divergence). The distributions are slightly different, albeit the divergence is substantially lower than for the RPE1 subset (JS K562: 0.04, JS RPE1: 0.16).

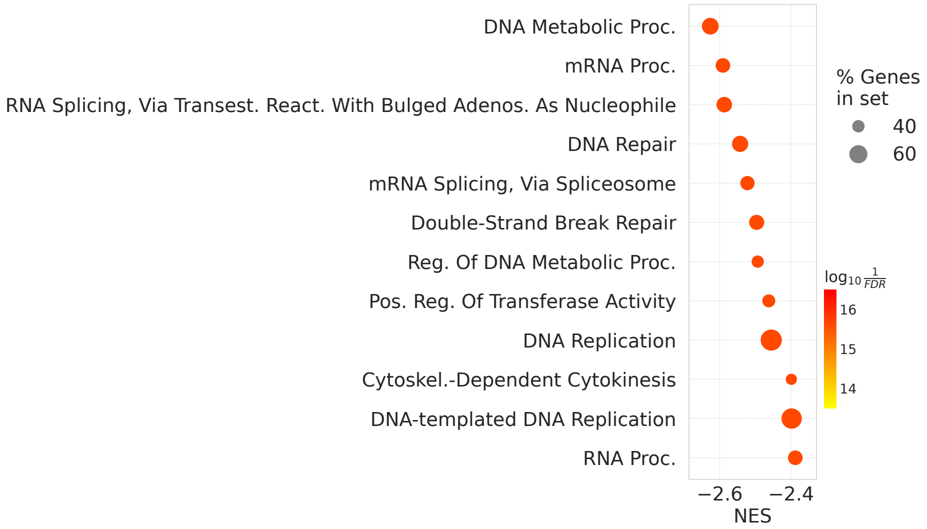

Supplementary Figure 16: Normalized enrichment scores and false discovery rate for top enriched pathways in the Replogle et al. (2022) (RPE1) dataset.

Supplementary Table 2: Lead genes that are differentially expressed consistently between perturbed and control cells in Replogle et al. (2022) RPE1 (two-sided t-test p-value < 0.05) for the top 10 enriched pathways.

**Pathway**	**Lead Genes**
DNA Metabolic Process	*DUT, MRE11, FANCG, RAD51AP1, POLD3, RFC3, RAN, HPF1, TOP2A, RPA3, POLQ, RMI2, CENPS, NTHL1, MSH6, PDS5A, NUDT1, XRCC5, DONSON, PDS5B, PRKDC, RFC5, RMI1, CHEK1, SMC1A, ORC1, TDP1, RAD21, POLE3, UBE2T, NPM1, CDC45, MCM4, SMARCA5, FANCL, SMARCB1, NSMCE4A, TOPBP1, TYMS, FEN1, KPNA2, RBBP8, RRM1, HLTF, BLM, PARP1, NEIL3, PRIM2, FAM111A, ACTL6A, UBE2N, DNA2, DCTPP1, UCHL5, BARD1, HMGB2, BRCA1, RFC2, POLA1, POLD2, MCM6, WDHD1, NUDT15, EXO1, LIG1, XRCC6, XRCC2, HMGB1, HMGB3, MCM2, MCRS1, NOC3L, MCM3, PTMS, MCM5, FAM111B, RFC4, MCM8, MCM10, RAD18, TIMELESS, TOP2B, ORC6, MSH2, RPA2, PARP2, POLE2, RECQL, RUVBL1, UNG, POLA2, TOP1, GEN1, CDK1, POLD1, MCM7, PRIM1, GTF2H3, FANCA, RAD51, PTGES3, CDK2, EP400, PCLAF, POLE*
mRNA Processing	*GEMIN5, GEMIN6, MAGOHB, SNRNP35, TRA2B, SRRM1, HNRNPA3, SNRNP200, SRSF6, RNPS1, SNRPE, LSM5, PPIH, DDX39A, SRSF2, CDC5L, SRSF1, ALYREF, CPSF3, HNRNPA1, DDX5, SRSF3, LSM2, DHX15, LSM6, SRSF10, LSM3, DDX46, SRSF4, SNRPB, RBMX, SNRPG, HSPA8, HNRNPH3, HNRNPM, RBM10, NCBP1, SNRNP40, HNRNPA0, SFPQ, PRPF8, HNRNPC, HTATSF1, HNRNPF, METTL3, HNRNPA2B1, U2AF2, HNRNPK, SNRPD1, SYNCRIP, MTREX, PNN, EIF4A3, SNRPF, TCERG1, PRPF19, LSM7, PRPF40A, SNRNP70, PRPF4, HNRNPU, SRSF7, RBM14, CPSF6, SNRPD3, SNRPA1, HNRNPH1, HNRNPR, SNRPA*
RNA Splicing, Via Transest.	*GEMIN5, GEMIN6, MAGOHB, SNRNP35, TRA2B, SRRM1, HNRNPA3, SNRNP200, SRSF6, RNPS1, SNRPE, LSM5, PPIH, DDX39A, SRSF2, CDC5L, SRSF1, HNRNPA1, DDX5, SRSF3, LSM2, DHX15, LSM6, SRSF10, LSM3, DDX46, SRSF4, SNRPB, RBMX, SNRPG, HSPA8, HNRNPH3, HNRNPM, RBM10, NCBP1, SNRNP40, PRPF8, HNRNPC, HTATSF1, HNRNPF, METTL3, HNRNPA2B1, U2AF2, HNRNPK, SNRPD1, SYNCRIP, MTREX, PNN, EIF4A3, SNRPF, PRPF19, LSM7, PRPF40A, SNRNP70, PRPF4, HNRNPU, SRSF7, RBM14, SNRPD3, SNRPA1, HNRNPH1, HNRNPR, SNRPA*
DNA Repair	*MRE11, FANCG, PCNA, RAD51AP1, POLD3, RFC3, HPF1, RPA3, POLQ, RMI2, CENPS, NTHL1, APEX2, FANCI, ZGRF1, MSH6, PDS5A, NSD2, TRIP13, RAD51D, NUDT1, XRCC5, PDS5B, PRKDC, RFC5, PARP4, CHEK1, SMC1A, TDP1, ASF1A, PRIMPOL, CDKN2D, RAD21, UBE2T, NPM1, HUWE1, SMARCA5, RNASEH2C, FANCL, NSMCE4A, TOPBP1, FEN1, RBBP8, RRM1, HLTF, BLM, PARP1, NEIL3, FAM111A, UBE2N, DNA2, BRCA2, XRCC1, BARD1, BRCA1, RFC2, POLA1, WDHD1, RHNO1, FANCD2, RAD51C, EXO1, FMR1, LIG1, XRCC6, RNASEH2B, CENPX, XRCC2, RFC4, CDCA5, ATR, RFWD3, RAD18, TIMELESS, DEPDC1B, MSH2, RPA2, PARP2, POLE2, RECQL, MLH1, UNG, RNF168, FSCN1, CDK1, POLD1, GTF2H3, PMS1, NUCKS1, FANCA, RAD51, EP400, POLE*
mRNA Splic., Via Spliceo.	*GEMIN5, CLNS1A, DHX9, MAGOHB, GEMIN6, TRA2B, SNRNP35, SRRM1, HNRNPA3, SNRNP200, SRSF6, RNPS1, SNRPE, LSM5, PPIH, DDX39A, RBM17, SRSF2, CDC5L, SRSF1, HNRNPA1, DDX5, SRSF3, LSM2, DHX15, LSM6, SRSF10, LSM3, DDX46, SRSF4, SNRPB, RBMX, SNRPG, HSPA8, HNRNPH3, HNRNPM, RBM10, NCBP1, SNRNP40, SFPQ, PRPF8, HNRNPC, HTATSF1, HNRNPF, METTL3, HNRNPA2B1, U2AF2, HNRNPK, SNRPD1, SYNCRIP, MTREX, PNN, EIF4A3, SNRPF, PRPF19, LSM7, PRPF40A, SNRNP70, PRPF4, HNRNPU, SRSF7, GEMIN4, RBM14, SNRPD3, SNRPA1, HNRNPH1, HNRNPR, SNRPA*
Double-Strand Break Repair	*BRCA2, MMS22L, MRE11, TDP1, CYREN, DEPDC1B, HMGB2, RAD21, RPA2, BRCA1, PARP2, POLA1, RAD51AP1, RECQL, HPF1, RPA3, TONSL, POLQ, RMI2, NSMCE4A, FEN1, SFPQ, XRCC6, RBBP8, ZGRF1, XRCC2, HMGB1, BLM, NUCKS1, NSD2, SLF2, PRPF19, TRIP13, PARP1, RAD51, SMC6, XRCC5, CHD4, SLF1, MCM8, PRKDC, UBE2N, RMI1, DNA2, CDCA5, RFWD3, PAXX*
Reg. Of DNA Metab. Proc.	*HNRNPD, UCHL5, TAF6, BAZ1A, PPP2CA, BARD1, DEPDC1B, BRCA1, POLE3, ALYREF, USP37, FBXO5, RUVBL1, SMARCA5, ENY2, NSMCE4A, CCNA2, TAF5, DSCC1, KPNA2, OBI1, NUCKS1, SLF2, CCDC88A, MCRS1, USP1, SMC6, XRCC5, SLF1, ESCO2, PIF1, SMC3, ACTL6A, UBE2N, GMNN, CHRAC1*
Pos. Reg. Of Transf. Act.	*HNRNPD, MRE11, PCNA, CCT4, MET, GINS4, TCP1, AXL, RFC2, EGFR, GINS2, RFC3, UBE2I, HSP90AA1, NEK2, DSCC1, DKC1, GINS1, HNRNPA2B1, CHTF18, PLK1, CCT2, PTGES3, XRCC5, AURKB, PRIM2, HSP90AB1, RFC4, RFC5, UBE2N*
DNA Replication	*CHEK1, DUT, BAZ1A, RPA2, RFC2, MCMBP, POLE3, POLA1, POLD2, POLE2, POLD3, RFC3, RPA3, MCM6, WDHD1, MCM4, POLA2, TOP1, FEN1, RFC5, CDK1, POLD1, MCM7, MCM2, BLM, RFC1, PTMS, CDK2, DONSON, FAM111A, MCM5, FAM111B, RFC4, DNA2, PCLAF, POLE, MCM8*
Cytosk.-Dep. Cytokinesis	*CKAP2, KIF20A, SEPTIN10, ANLN, MYH10, KIF23, AURKB, CIT, NUSAP1, ECT2, BIRC5, RACGAP1, STMN1, PLK1, CDCA8, CEP55, CENPA, KIF4A*

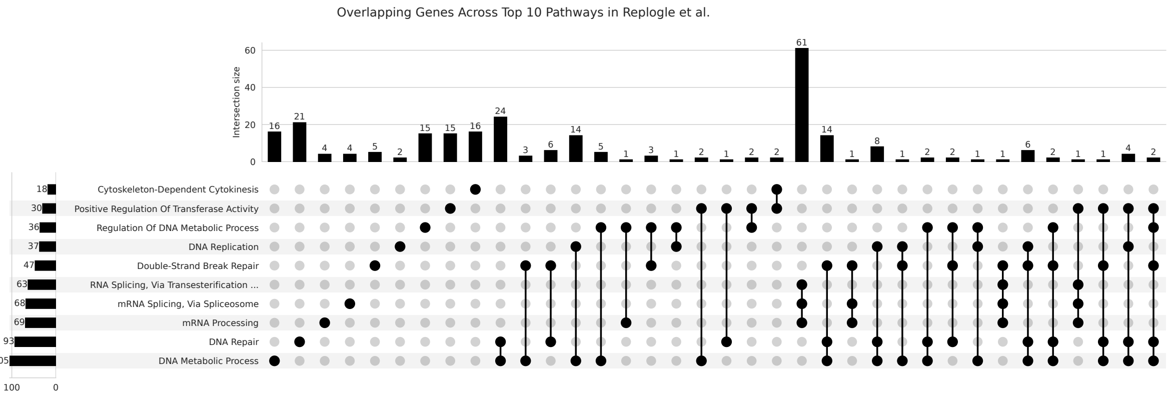

Supplementary Figure 17: Upset plot showing the number of differentially expressed (DE) genes (two-sided t-test p-value < 0.05) overlapping among the top 10 pathways in Replogle et al (RPE1). Left bars: number of DE genes per pathway.

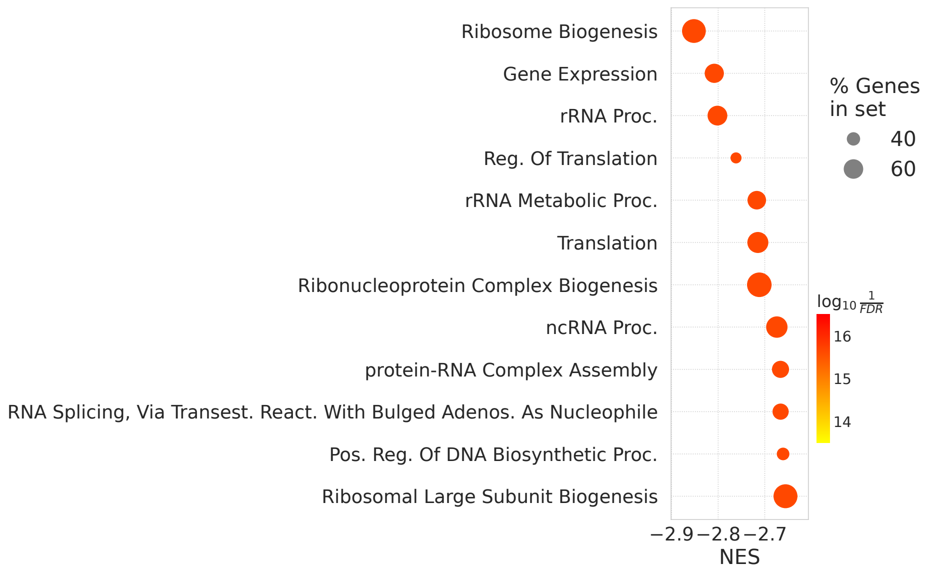

Supplementary Figure 18: Normalized enrichment scores and false discovery rate for top enriched pathways in the Replogle et al. (2022) (K562) dataset.

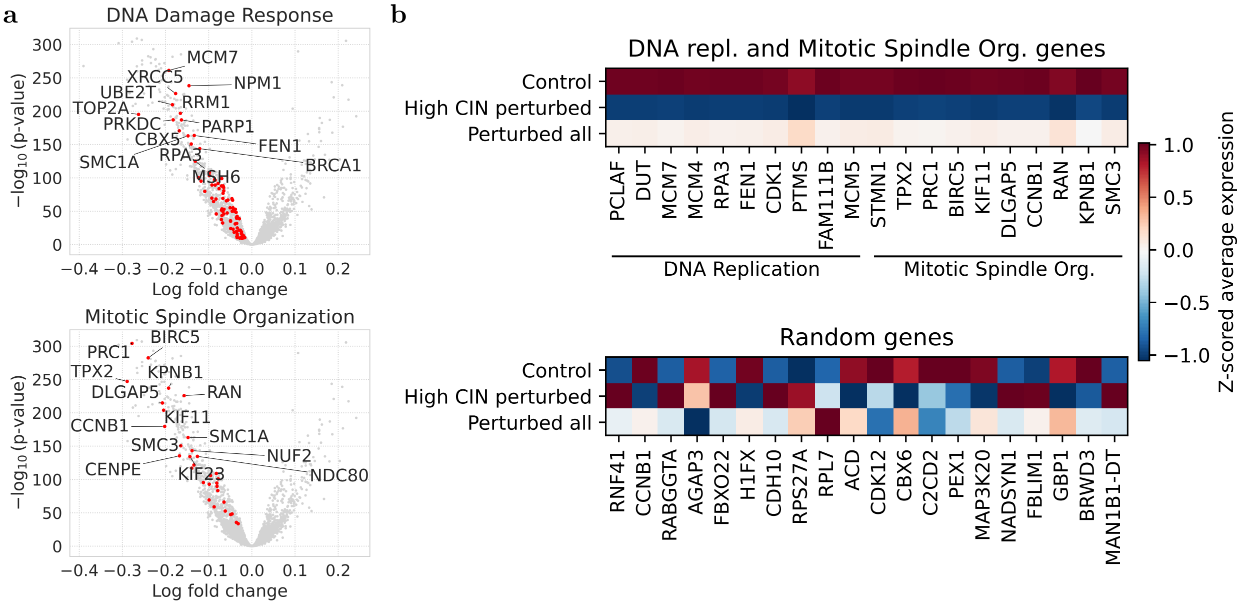

Supplementary Figure 19: **Volcano and heatmap plots for two selected enriched pathways in Replogle et al. (2022) RPE1**. (**a**) Volcano plot depicting the log-fold changes (perturbed vs control cells) for gene sets involved in DNA replication and mitotic spindle organization. (**b**) Top heatmap: Expression of the top differentially expressed DNA replication and mitotic spindle organization genes in three conditions: control, perturbed cells with high chromosomal instability (perturbation CIN z-score > 2), and all perturbed cells. Bottom heatmap: Expression of a random subset of genes. The expression of genes in the cell cycle and DNA replication pathways is consistently lower in perturbed cells.

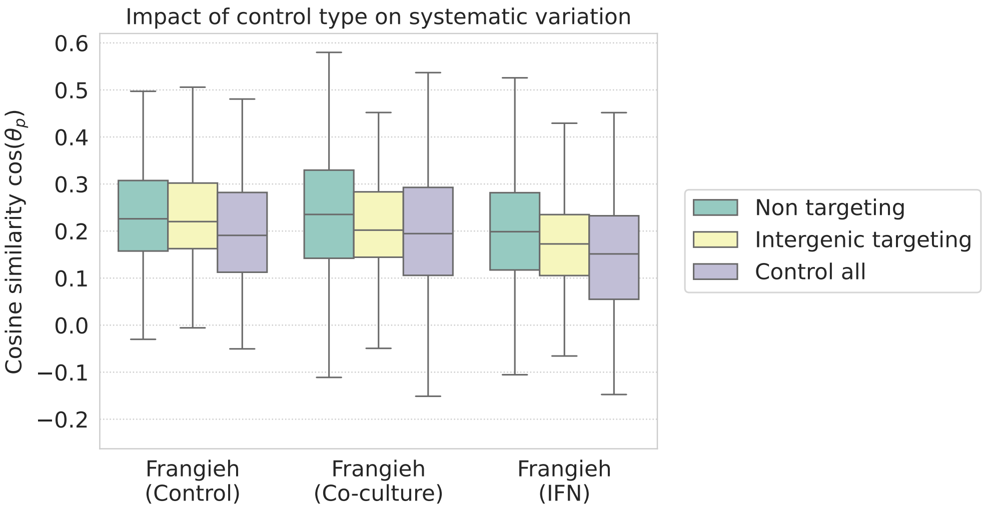

Supplementary Figure 20: Distribution of cosine similarities between perturbation-specific shifts and average perturbation effect by control type in Frangieh et al. (2021). Frangieh et al. (2021) used both non-targeting and intergenic sgRNAs as negative controls. We compute the cosine similarity of perturbation-specific shifts with the average perturbation effect using 3 references: 1) centroid of cells with non-targeting sgRNAs, 2) centroid of cells with intergenic sgRNAs, and 3) centroid of all control cells (control all, including cells with non-targeting and intergenic sgRNAs). We use the same set of n=167 perturbations for all conditions and control types. Overall, we did not observe substantial differences between control types. Boxes depict distribution quartiles, with the center line corresponding to the median, and whiskers span 1.5 times the interquartile range.

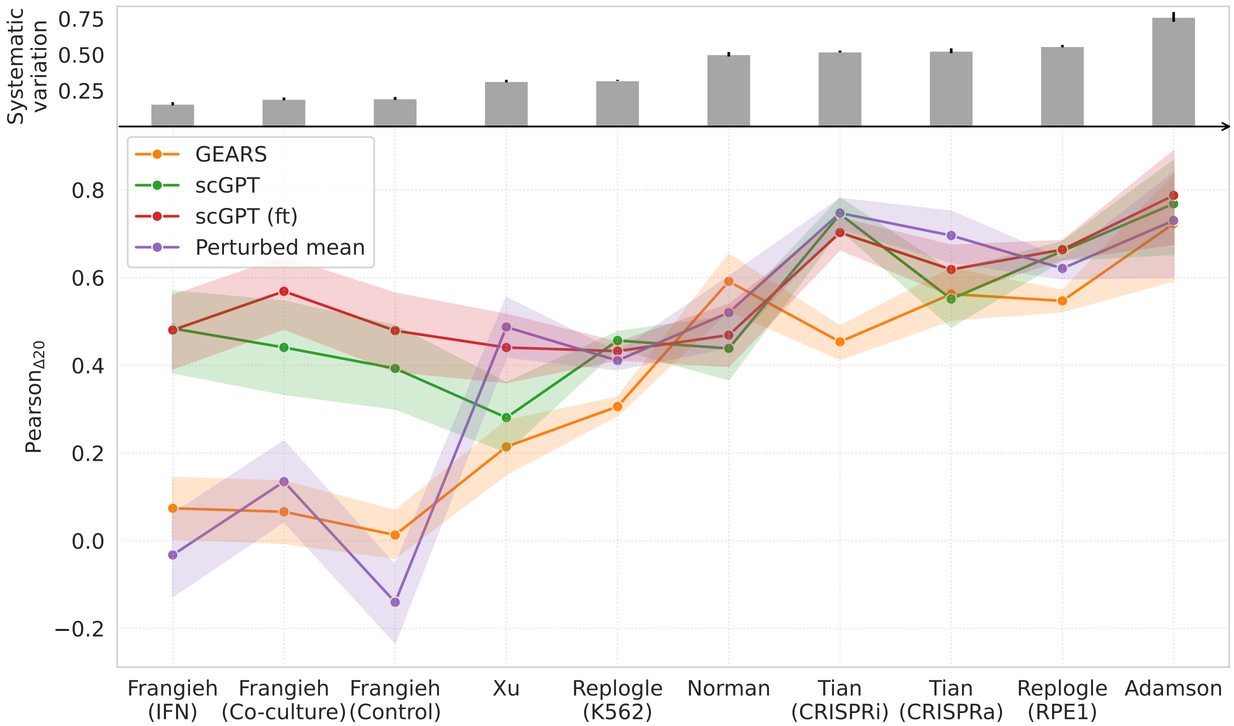

Supplementary Figure 21: Relationship between the performance of perturbation response prediction methods and systematic variation. The performance was measured using Pearson$_{\Delta 20}$ correlation (*i.e.*, using the top 20 differentially expressed genes of each perturbation) on differential expression profiles of the 1-gene test perturbations. Datasets are sorted by our measure of systematic variation. Error bars depict the 95% confidence interval of the scores across 3 independent runs with different data splits. The gray bars in the bar plot depict the degree of systematic variation in each dataset and error bars show the 95% confidence interval (Adamson: $n = 81$, Norman: $n = 276$, Replogle K562: $n = 1813$, Replogle RPE1: $n = 1410$, Tian CRISPRa: $n = 97$, Tian CRISPRi: $n = 181$, Xu: $n = 198$, Frangieh control: $n = 167$, Frangieh co-culture: $n = 167$, Frangieh interferon: $n = 167$; n is the total number of perturbations per dataset).

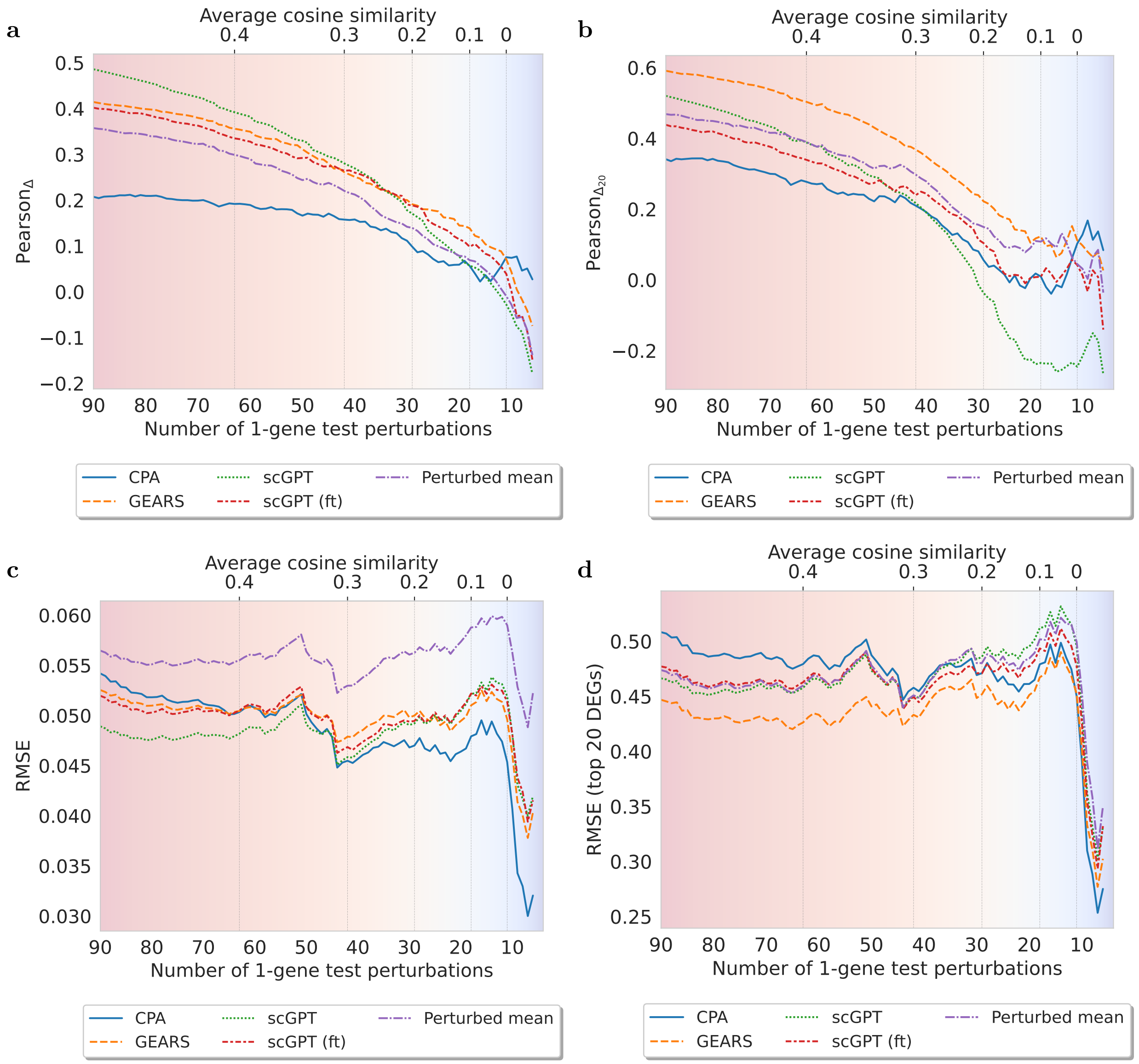

Supplementary Figure 22: Subsampling analysis on the Norman et al. (2019) dataset. We studied the extent to which the choice of 1-gene test perturbations influences predictive scores for each method. In particular, we ranked the 1-gene test perturbations based on their cosine similarity with the average shift (perturbed centroid - control centroid). We then iteratively subsampled the perturbation that was most aligned with the average shift and computed different metrics on the remaining test perturbations: (**a**, **b**) Pearson correlation on differential expression profiles using (**a**) all genes and (**b**) top 20 differentially expressed genes per perturbation. (**c**, **d**) RMSE score using (**c**) all genes and (**d**) top 20 differentially expressed genes per perturbation. y-axis: Performance for each subsample. Bottom x-axis: Sample size (ranked perturbations). Top x-axis: average cosine similarity quantifying perturbation bias. These results show that all methods behave similarly to the perturbed mean baseline regardless of sample size, with GEARS achieving marginally better performance in terms of Pearson correlation (top 20 DEGs) and RMSE (top 20 DEGs).

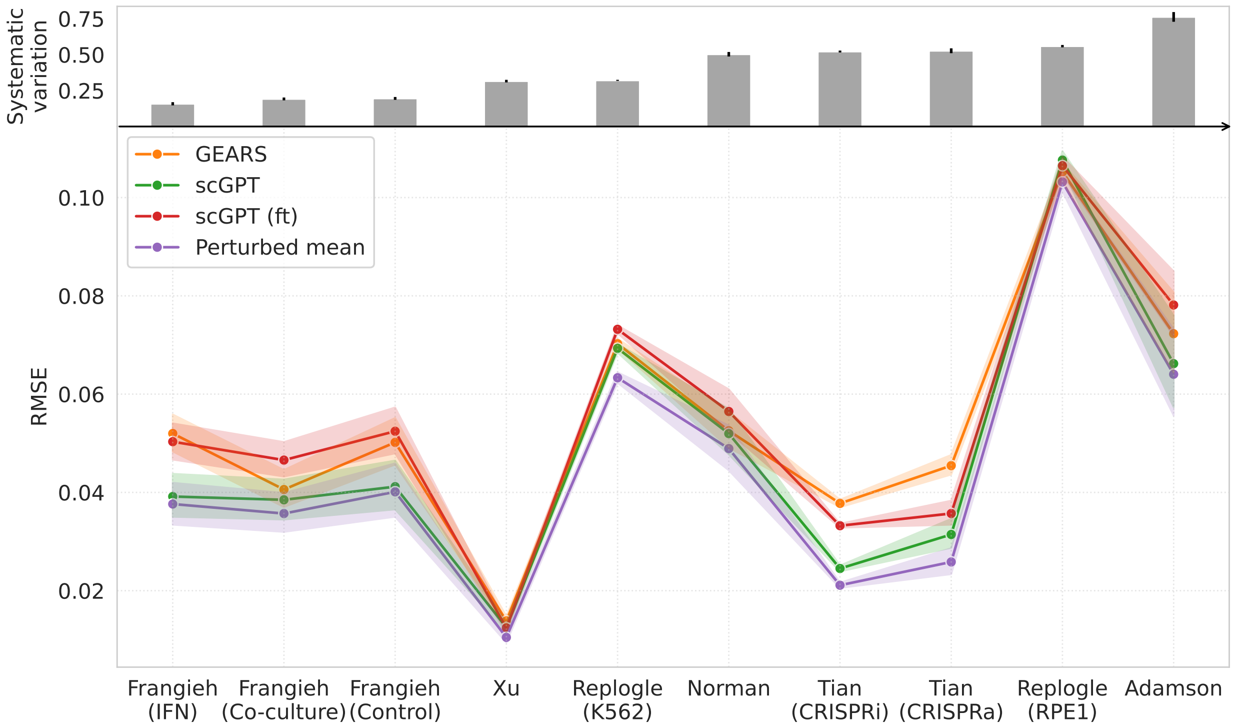

Supplementary Figure 23: Relationship between the performance of perturbation response prediction methods and systematic variation. The performance was measured using RMSE on the 1-gene test perturbations. Datasets are sorted by our measure of systematic variation. Error bars depict the 95% confidence interval of the scores across 3 independent runs with different data splits. The gray bars in the bar plot depict the degree of systematic variation in each dataset and error bars show the 95% confidence interval (Adamson: $n = 81$, Norman: $n = 276$, Replogle K562: $n = 1813$, Replogle RPE1: $n = 1410$, Tian CRISPRa: $n = 97$, Tian CRISPRi: $n = 181$, Xu: $n = 198$, Frangieh control: $n = 167$, Frangieh co-culture: $n = 167$, Frangieh interferon: $n = 167$; n is the total number of perturbations per dataset).

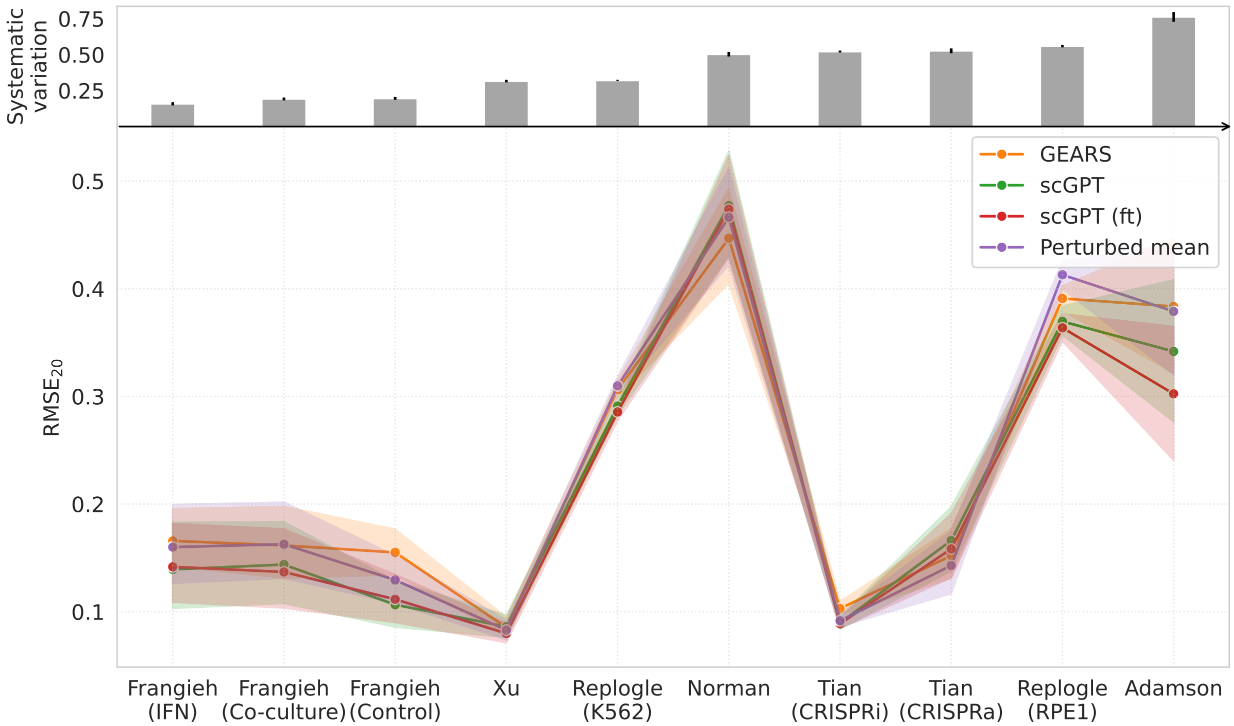

Supplementary Figure 24: Relationship between the performance of perturbation response prediction methods and systematic variation. The performance was measured using RMSE$_{20}$ (*i.e.*, using the top 20 differentially expressed genes of each perturbation) on the 1-gene test perturbations. Datasets are sorted by our measure of systematic variation. Error bars depict the 95% confidence interval of the scores across 3 independent runs with different data splits. The gray bars in the bar plot depict the degree of systematic variation in each dataset and error bars show the 95% confidence interval (Adamson: $n = 81$, Norman: $n = 276$, Replogle K562: $n = 1813$, Replogle RPE1: $n = 1410$, Tian CRISPRa: $n = 97$, Tian CRISPRi: $n = 181$, Xu: $n = 198$, Frangieh control: $n = 167$, Frangieh co-culture: $n = 167$, Frangieh interferon: $n = 167$; n is the total number of perturbations per dataset).

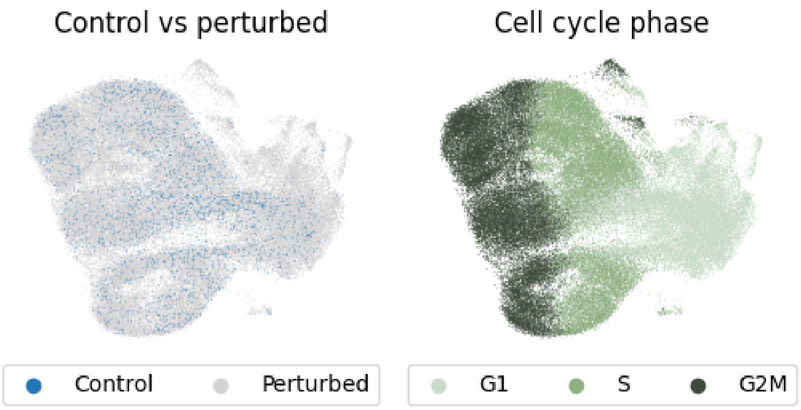

Supplementary Figure 25: UMAP plot of normalized gene expression values in our downsampled Replogle RPE1 dataset colored by control vs perturbed cells (left) and cell cycle phase (right). We downsampled perturbed cells to approximately match the phase distribution of control cells (control, G1: 25%, S: 35%, G2M: 40%; perturbed: G1: 28%, S: 34%, G2M: 38%).

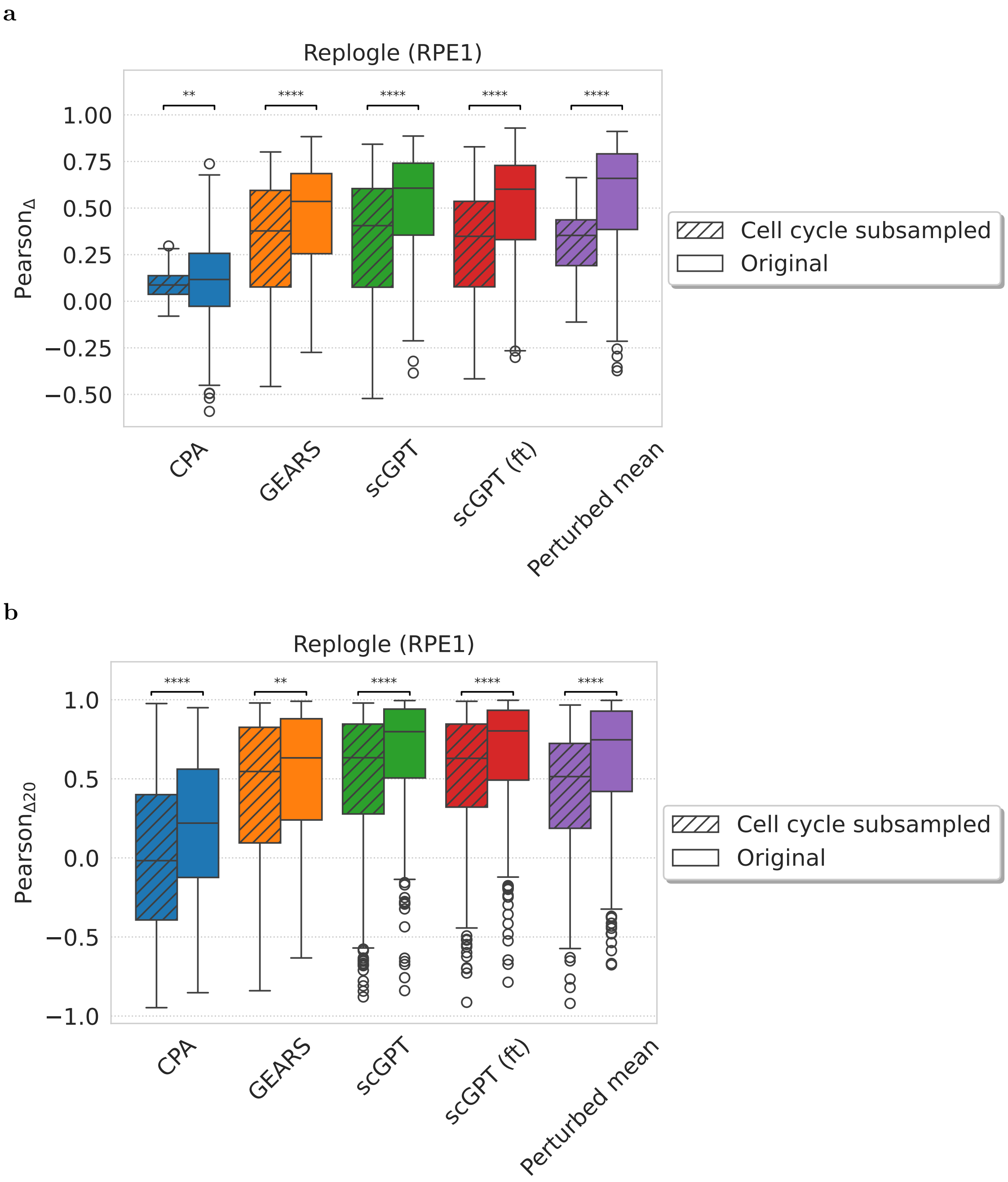

Supplementary Figure 26: Cell cycle phase distribution discrepancies between perturbed and control cells strongly impact Pearson$_\Delta$ and Pearson$_{\Delta 20}$ scores. We compared the predictive performance of models trained and evaluated on 1) Replogle RPE1 and 2) another version of Replogle RPE1 downsampled to approximately match the cell cycle phase distributions between perturbed and control cells (control, G1: 25%, S: 35%, G2M: 40%; perturbed: G1: 28%, S: 34%, G2M: 38%). We evaluated the models on the same set of test perturbations. (a) Distribution of Pearson$_\Delta$ scores. (b) Distribution of Pearson$_{\Delta 20}$ scores. Number n test perturbations across a single run: $n = 353$ (these perturbations match in the two dataset versions). We performed two-sided paired t-tests to compare the distributions of predictive scores for the two dataset versions. Significance is denoted as: ns ($p > 0.05$), * ($p \leq 0.05$), ** ($p \leq 0.01$), *** ($p \leq 0.001$), **** ($p \leq 0.0001$). Boxes depict distribution quartiles, with the center line corresponding to the median, and whiskers span 1.5 times the interquartile range.

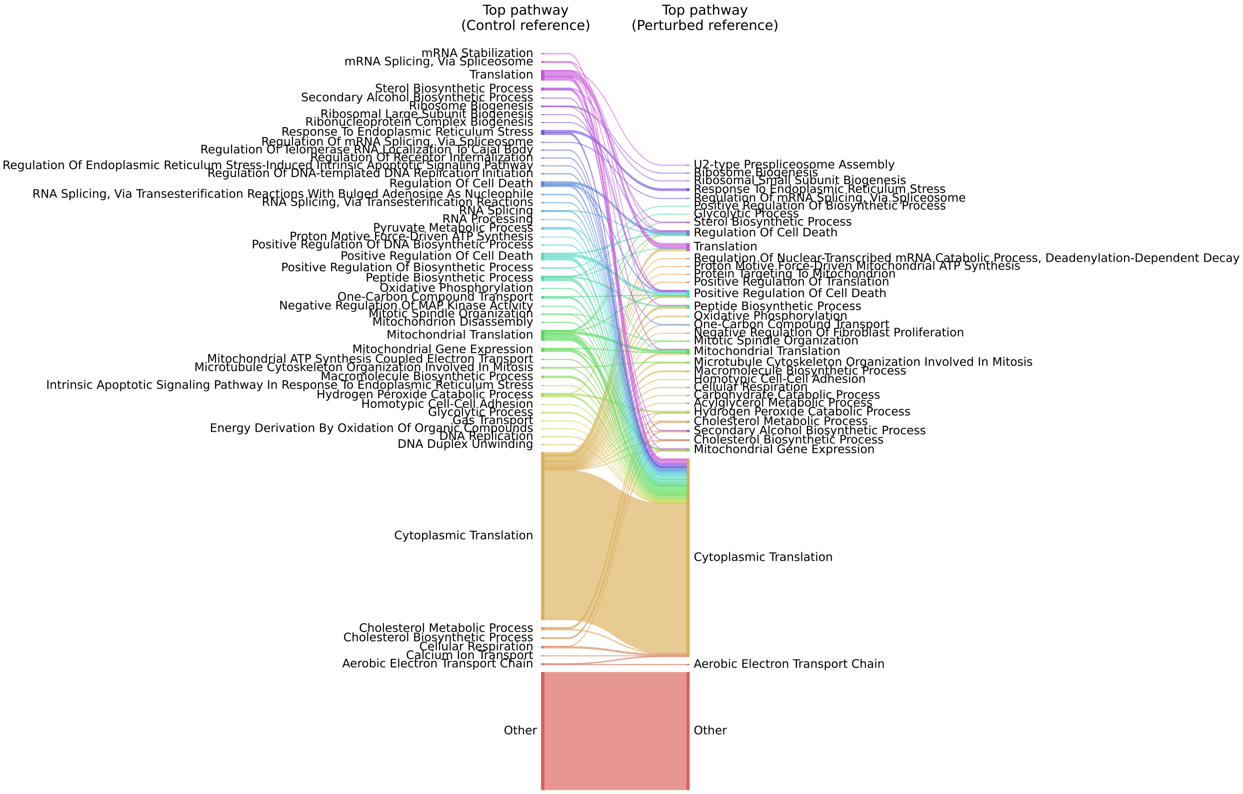

Supplementary Figure 27: Alluvial plot showing the top gene set for all 1,813 perturbations of the genome-wide Replogle et al. (2022) K562 perturbation screen when using the control (left) and perturbed centroid as reference (left). To identify the top pathway, we performed Preranked Gene Set Enrichment Analysis (Subramanian et al., 2005) on the average expression changes of each perturbation with respect to the control (left) and perturbed (right) references. We used *Biological Process 2023* gene sets from the Gene Ontology (GO) (Aleksander et al., 2023; Ashburner et al., 2000). For ease of visualization, we grouped unique pairs (top pathway control reference, top pathway perturbed reference) into the *Other* category.

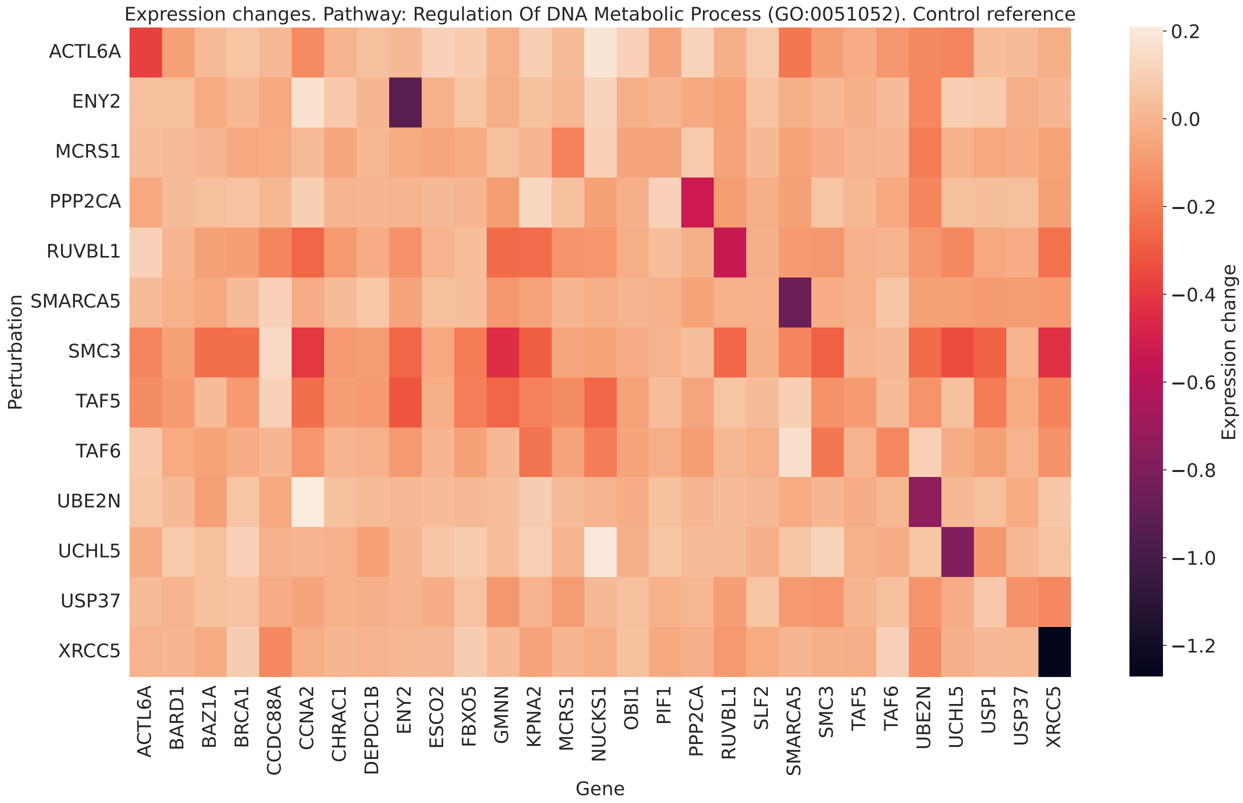

Supplementary Figure 28: Heatmap showing the average expression changes of DNA metabolic process genes with respect to the *control* reference after perturbing genes involved in the regulation of DNA metabolic processes.

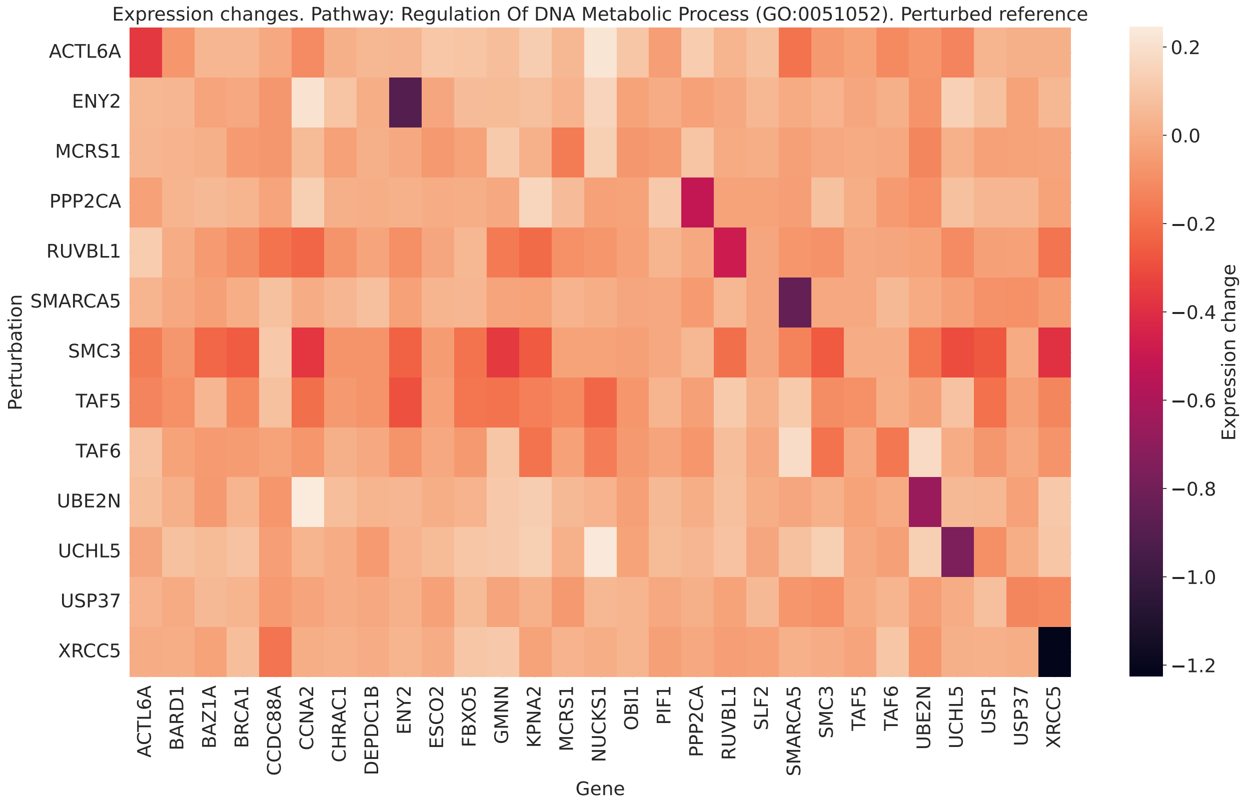

Supplementary Figure 29: Heatmap showing the average expression changes of DNA metabolic process genes with respect to the *perturbed* reference after perturbing genes involved in the regulation of DNA metabolic processes.

Supplementary Table 3: Table showing the average expression changes of DNA metabolic process genes with respect to the *control* reference after perturbing genes involved in the regulation of DNA metabolic processes. Columns: Perturbations of genes involved in the regulation of DNA metabolic processes. Rows: Genes involved in the regulation of DNA metabolic processes.

	ACTL6A	*ENY2*	*MCRS1*	*PPP2CA*	*RUVBL1*	*SMARCA5*	*SMC3*	*TAF5*	*TAF6*	*UBE2N*	*UCHL5*	*USP37*	*XRCC5*
ACTL6A	-0.4	0.0	0.0	-0.0	0.1	0.0	-0.2	-0.1	0.1	0.1	-0.0	0.0	-0.0
BARD1	-0.1	0.0	0.0	0.0	-0.0	-0.0	-0.1	-0.1	-0.0	0.0	0.1	-0.0	0.0
BAZ1A	0.0	-0.0	0.0	0.0	-0.1	-0.1	-0.2	0.0	-0.1	-0.1	0.0	0.0	-0.0
BRCA1	0.1	0.0	-0.0	0.1	-0.1	0.0	-0.2	-0.1	-0.0	0.1	0.1	0.1	0.1
CCDC88A	0.0	-0.0	-0.0	0.0	-0.2	0.1	0.1	0.1	0.0	-0.0	-0.0	-0.0	-0.2
CCNA2	-0.1	0.2	0.0	0.1	-0.3	-0.0	-0.4	-0.2	-0.1	0.2	-0.0	-0.1	-0.0
CHRAC1	-0.0	0.1	-0.1	-0.0	-0.1	0.0	-0.1	-0.1	-0.0	0.0	-0.0	-0.0	0.0
DEPDC1B	0.0	0.0	0.0	-0.0	-0.0	0.1	-0.1	-0.1	-0.0	0.0	-0.1	-0.0	-0.0
ENY2	0.0	-0.9	-0.0	-0.0	-0.1	-0.1	-0.3	-0.3	-0.1	0.0	0.0	-0.0	0.0
ESCO2	0.1	-0.0	-0.1	0.0	-0.0	0.0	-0.0	-0.0	0.0	0.0	0.1	-0.0	0.0
FBXO5	0.1	0.1	-0.0	0.0	0.0	0.0	-0.2	-0.2	-0.1	0.0	0.1	0.0	0.1
GMNN	-0.0	-0.0	0.0	-0.1	-0.2	-0.1	-0.4	-0.3	0.0	0.0	0.0	-0.1	0.0
KPNA2	0.1	0.0	-0.0	0.1	-0.2	-0.1	-0.3	-0.2	-0.2	0.1	0.1	-0.0	-0.1
MCRS1	0.0	0.0	-0.2	0.0	-0.1	0.0	-0.1	-0.1	-0.1	0.0	-0.0	-0.1	0.0
NUCKS1	0.2	0.1	0.1	-0.1	-0.1	-0.0	-0.1	-0.3	-0.2	-0.0	0.2	0.0	-0.0
OBI1	0.1	-0.0	-0.1	-0.0	-0.0	-0.0	-0.0	-0.1	-0.1	-0.0	-0.0	0.0	0.0
PIF1	-0.1	0.0	-0.1	0.1	0.0	-0.0	-0.0	0.0	-0.0	0.0	0.1	-0.0	-0.0
PPP2CA	0.1	-0.0	0.1	-0.5	-0.0	-0.1	0.0	-0.1	-0.1	0.0	0.0	0.0	-0.0
RUVBL1	-0.0	-0.1	-0.1	-0.1	-0.5	-0.0	-0.3	0.1	0.0	0.0	0.0	-0.1	-0.1
SLF2	0.1	0.0	0.0	-0.0	-0.0	-0.0	-0.0	0.0	-0.0	0.0	-0.0	0.1	-0.0
SMARCA5	-0.2	-0.0	-0.1	-0.1	-0.1	-0.9	-0.2	0.1	0.2	-0.0	0.1	-0.1	0.0
SMC3	-0.1	0.0	-0.0	0.1	-0.1	-0.0	-0.3	-0.1	-0.2	0.0	0.1	-0.1	-0.0
TAF5	-0.0	-0.0	-0.0	0.0	-0.0	-0.0	0.0	-0.1	-0.0	-0.0	-0.0	-0.0	-0.0
TAF6	-0.1	0.0	0.0	-0.1	-0.0	0.1	0.0	0.0	-0.2	0.0	-0.0	0.0	0.1
UBE2N	-0.2	-0.2	-0.2	-0.2	-0.1	-0.1	-0.3	-0.1	0.1	-0.7	0.1	-0.1	-0.2
UCHL5	-0.2	0.1	-0.0	0.0	-0.2	-0.1	-0.3	0.0	-0.0	0.0	-0.8	-0.0	-0.0
USP1	0.0	0.1	-0.0	0.0	-0.0	-0.1	-0.3	-0.2	-0.1	0.0	-0.1	0.1	0.0
USP37	0.0	-0.0	-0.0	0.0	-0.0	-0.1	-0.0	-0.0	-0.0	-0.0	0.0	-0.1	0.0
XRCC5	-0.0	0.0	-0.1	-0.1	-0.2	-0.1	-0.4	-0.2	-0.1	0.1	0.0	-0.2	-1.3

Supplementary Table 4: Table showing the average expression changes of DNA metabolic process genes with respect to the *perturbed* reference after perturbing genes involved in the regulation of DNA metabolic processes. Columns: Perturbations of genes involved in the regulation of DNA metabolic processes. Rows: Genes involved in the regulation of DNA metabolic processes.

	ACTL6A	*ENY2*	*MCRS1*	*PPP2CA*	*RUVBL1*	*SMARCA5*	*SMC3*	*TAF5*	*TAF6*	*UBE2N*	*UCHL5*	*USP37*	*XRCC5*
ACTL6A	-0.4	0.0	0.0	-0.0	0.1	0.0	-0.2	-0.1	0.1	0.1	-0.0	0.0	0.0
BARD1	-0.1	0.0	0.0	0.0	0.0	-0.0	-0.1	-0.1	-0.0	0.0	0.1	-0.0	0.0
BAZ1A	0.0	-0.0	0.0	0.1	-0.1	-0.0	-0.2	0.0	-0.1	-0.1	0.1	0.1	-0.0
BRCA1	0.0	-0.0	-0.1	0.0	-0.1	0.0	-0.3	-0.1	-0.1	0.0	0.1	0.0	0.1
CCDC88A	-0.0	-0.1	-0.1	-0.0	-0.2	0.1	0.1	0.1	-0.0	-0.1	-0.0	-0.1	-0.2
CCNA2	-0.1	0.2	0.1	0.1	-0.2	0.0	-0.4	-0.2	-0.1	0.2	0.0	-0.0	0.0
CHRAC1	0.0	0.1	-0.0	0.0	-0.1	0.0	-0.1	-0.1	0.0	0.1	0.0	0.0	0.0
DEPDC1B	0.0	0.0	0.0	0.0	-0.0	0.1	-0.1	-0.1	-0.0	0.0	-0.1	-0.0	0.0
ENY2	0.0	-0.9	-0.0	0.0	-0.1	-0.0	-0.2	-0.3	-0.1	0.0	0.0	0.0	0.0
ESCO2	0.1	-0.0	-0.1	0.0	-0.0	0.0	-0.1	-0.0	-0.0	0.0	0.1	-0.0	0.0
FBXO5	0.1	0.1	-0.0	0.0	0.0	0.0	-0.2	-0.2	-0.1	0.0	0.1	0.1	0.1
GMNN	0.1	0.1	0.1	-0.0	-0.2	-0.0	-0.4	-0.2	0.1	0.1	0.1	-0.0	0.1
KPNA2	0.1	0.1	0.0	0.2	-0.2	-0.0	-0.3	-0.1	-0.2	0.1	0.1	0.0	-0.0
MCRS1	0.0	0.0	-0.2	0.1	-0.1	0.0	-0.0	-0.1	-0.0	0.1	0.0	-0.1	0.0
NUCKS1	0.2	0.2	0.1	-0.0	-0.1	0.0	-0.0	-0.2	-0.2	0.0	0.2	0.0	0.0
OBI1	0.1	-0.0	-0.1	-0.0	-0.0	-0.0	-0.0	-0.1	-0.1	-0.0	-0.0	0.0	0.0
PIF1	-0.0	0.0	-0.1	0.1	0.0	-0.0	-0.0	0.0	-0.0	0.0	0.1	-0.0	-0.0
PPP2CA	0.1	-0.0	0.1	-0.5	-0.0	-0.1	0.0	-0.0	-0.1	0.0	0.0	0.0	-0.0
RUVBL1	0.0	-0.0	0.0	-0.0	-0.5	0.0	-0.2	0.1	0.1	0.1	0.1	-0.0	-0.0
SLF2	0.1	0.0	0.0	-0.0	-0.0	-0.0	-0.0	0.0	-0.0	0.0	-0.0	0.1	-0.0
SMARCA5	-0.2	0.0	-0.0	-0.0	-0.1	-0.8	-0.1	0.1	0.2	-0.0	0.1	-0.1	0.0
SMC3	-0.1	0.0	-0.0	0.1	-0.1	-0.0	-0.3	-0.1	-0.2	0.0	0.1	-0.1	0.0
TAF5	-0.0	-0.0	-0.0	0.0	-0.0	-0.0	0.0	-0.1	-0.0	-0.0	-0.0	0.0	-0.0
TAF6	-0.1	0.0	-0.0	-0.1	-0.0	0.1	0.0	0.0	-0.2	-0.0	-0.0	0.0	0.1
UBE2N	-0.1	-0.1	-0.1	-0.1	-0.0	-0.0	-0.2	-0.0	0.2	-0.7	0.1	-0.0	-0.1
UCHL5	-0.1	0.1	0.0	0.1	-0.1	-0.0	-0.3	0.1	0.0	0.1	-0.8	0.0	0.0
USP1	0.0	0.1	-0.0	0.0	-0.0	-0.1	-0.3	-0.2	-0.1	0.0	-0.1	0.1	0.0
USP37	0.0	-0.0	-0.0	0.0	-0.0	-0.1	-0.0	-0.0	-0.0	-0.0	0.0	-0.1	0.0
XRCC5	0.0	0.0	-0.0	-0.0	-0.2	-0.1	-0.4	-0.1	-0.1	0.1	0.1	-0.1	-1.2

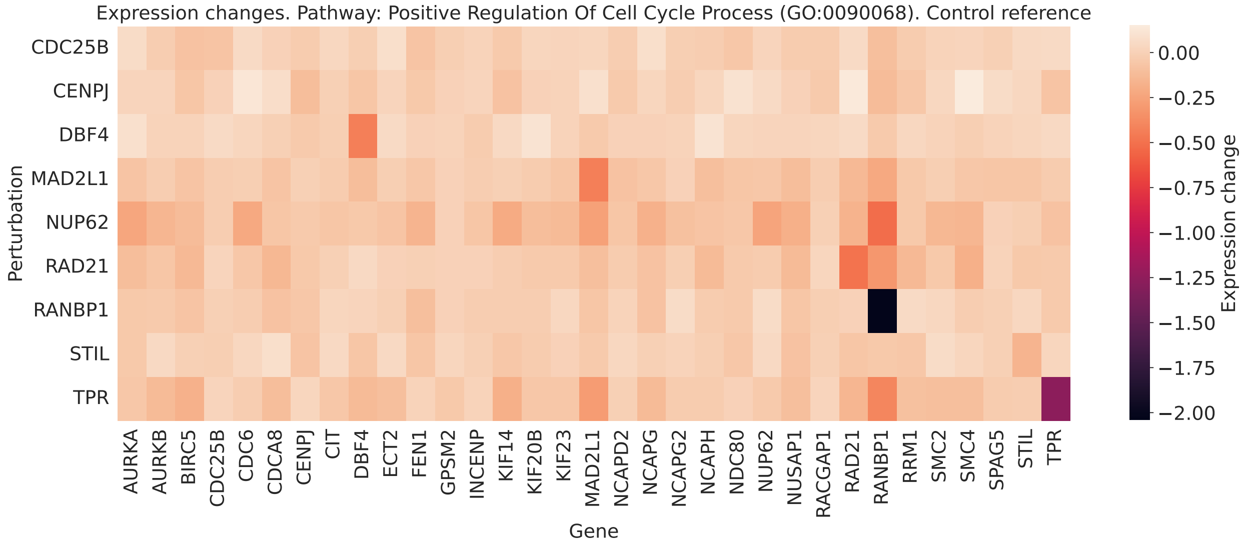

Supplementary Figure 30: Heatmap showing the average expression changes of cell cycle genes with respect to the *control* reference after perturbing genes involved in the regulation of cell cycle.

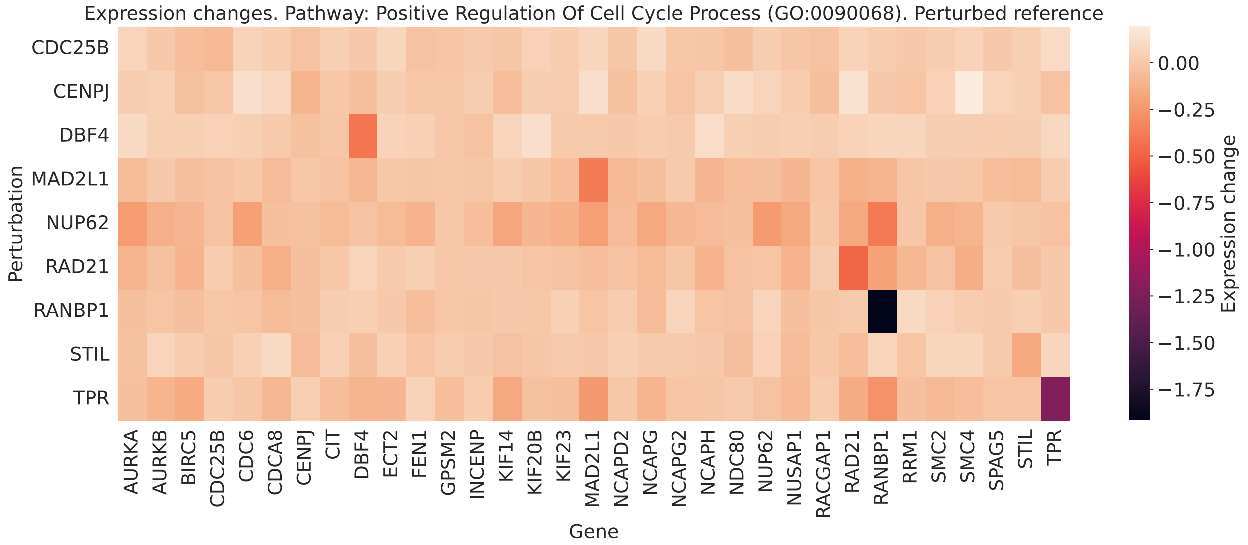

Supplementary Figure 31: Heatmap showing the average expression changes of cell cycle genes with respect to the *perturbed* reference after perturbing genes involved in the regulation of cell cycle.

Supplementary Table 5: Table showing the average expression changes of cell cycle genes with respect to the *control* reference after perturbing genes involved in the regulation of cell cycle. Columns: Perturbations of genes involved in the regulation of cell cycle. Rows: Genes involved in the regulation of cell cycle.

	CDC25B	*CENPJ*	*DBF4*	*MAD2L1*	*NUP62*	*RAD21*	*RANBP1*	*STIL*	*TPR*
AURKA	0.1	0.0	0.1	-0.1	-0.2	-0.1	-0.1	-0.1	-0.1
AURKB	-0.0	0.0	0.0	-0.0	-0.2	-0.1	-0.1	0.0	-0.1
BIRC5	-0.1	-0.1	0.0	-0.1	-0.1	-0.1	-0.1	-0.0	-0.2
CDC25B	-0.1	-0.0	0.0	-0.0	-0.0	0.0	-0.0	-0.0	0.0
CDC6	0.0	0.1	0.0	-0.0	-0.2	-0.1	-0.0	0.0	-0.0
CDCA8	-0.0	0.1	-0.0	-0.1	-0.1	-0.2	-0.1	0.1	-0.1
CENPJ	-0.0	-0.1	-0.1	-0.0	-0.0	-0.1	-0.1	-0.1	0.0
CIT	0.0	-0.0	-0.0	-0.0	-0.1	-0.0	0.0	0.0	-0.1
DBF4	-0.0	-0.1	-0.4	-0.1	-0.1	0.0	0.0	-0.1	-0.1
ECT2	0.1	0.0	0.0	-0.0	-0.1	-0.0	-0.0	0.0	-0.1
FEN1	-0.1	-0.1	-0.0	-0.1	-0.2	-0.0	-0.1	-0.1	0.0
GPSM2	-0.0	-0.0	0.0	-0.0	-0.0	-0.0	-0.0	0.0	-0.1
INCENP	-0.0	0.0	-0.0	-0.0	-0.1	-0.0	-0.0	-0.0	0.0
KIF14	-0.0	-0.1	0.0	-0.0	-0.2	-0.0	-0.0	-0.1	-0.2
KIF20B	0.0	-0.0	0.1	-0.0	-0.1	-0.0	-0.0	-0.0	-0.1
KIF23	0.0	0.0	0.0	-0.1	-0.1	-0.0	0.0	-0.0	-0.1
MAD2L1	0.0	0.1	-0.0	-0.4	-0.3	-0.1	-0.1	-0.0	-0.3
NCAPD2	-0.0	-0.0	-0.0	-0.1	-0.1	-0.0	0.0	0.0	-0.0
NCAPG	0.1	0.0	-0.0	-0.1	-0.2	-0.1	-0.1	-0.0	-0.1
NCAPG2	-0.0	-0.0	0.0	-0.0	-0.1	-0.0	0.1	0.0	-0.0
NCAPH	-0.0	0.0	0.1	-0.1	-0.1	-0.1	-0.0	-0.0	-0.0
NDC80	-0.1	0.1	0.0	-0.1	-0.1	-0.0	-0.0	-0.1	-0.0
NUP62	0.0	0.0	0.0	-0.1	-0.3	-0.0	0.1	0.0	-0.1
NUSAP1	-0.0	-0.0	0.0	-0.1	-0.2	-0.1	-0.1	-0.1	-0.1
RACGAP1	-0.0	-0.1	0.0	-0.0	-0.0	0.0	-0.0	-0.0	0.0
RAD21	0.0	0.1	0.0	-0.1	-0.2	-0.5	-0.0	-0.1	-0.2
RANBP1	-0.1	-0.1	-0.1	-0.2	-0.5	-0.3	-2.0	-0.1	-0.4
RRM1	-0.0	-0.1	0.0	-0.1	-0.1	-0.1	0.0	-0.1	-0.1
SMC2	0.0	0.0	0.0	-0.0	-0.2	-0.1	0.0	0.1	-0.1
SMC4	0.0	0.1	-0.0	-0.1	-0.2	-0.2	-0.0	0.0	-0.1
SPAG5	-0.0	0.1	-0.0	-0.1	-0.0	0.0	-0.0	-0.0	-0.0
STIL	0.0	0.0	0.0	-0.1	-0.0	-0.1	0.0	-0.2	-0.0
TPR	0.0	-0.1	0.0	-0.0	-0.1	-0.1	-0.1	0.0	-1.3

Supplementary Table 6: Table showing the average expression changes of cell cycle genes with respect to the *perturbed* reference after perturbing genes involved in the regulation of cell cycle. Columns: Perturbations of genes involved in the regulation of cell cycle. Rows: Genes involved in the regulation of cell cycle.

	CDC25B	*CENPJ*	*DBF4*	*MAD2L1*	*NUP62*	*RAD21*	*RANBP1*	*STIL*	*TPR*
AURKA	0.1	0.0	0.1	-0.1	-0.2	-0.1	-0.0	-0.0	-0.1
AURKB	-0.0	0.0	0.0	-0.0	-0.1	-0.0	-0.0	0.1	-0.1
BIRC5	-0.1	-0.0	0.0	-0.1	-0.1	-0.1	-0.1	0.0	-0.2
CDC25B	-0.1	-0.0	0.0	-0.0	-0.0	0.0	-0.0	-0.0	0.0
CDC6	0.1	0.1	0.0	-0.0	-0.2	-0.1	-0.0	0.0	-0.0
CDCA8	0.0	0.1	0.0	-0.1	-0.1	-0.1	-0.1	0.1	-0.1
CENPJ	-0.0	-0.1	-0.0	-0.0	-0.0	-0.1	-0.1	-0.1	0.0
CIT	0.0	-0.0	-0.0	-0.0	-0.1	-0.0	0.0	0.0	-0.1
DBF4	0.0	-0.0	-0.4	-0.1	-0.0	0.1	0.0	-0.1	-0.1
ECT2	0.1	0.0	0.1	-0.0	-0.1	0.0	-0.0	0.0	-0.1
FEN1	-0.0	-0.0	0.0	-0.0	-0.1	0.0	-0.1	-0.0	0.1
GPSM2	-0.0	-0.0	-0.0	-0.0	-0.0	-0.0	-0.0	0.0	-0.1
INCENP	0.0	0.0	-0.0	-0.0	-0.1	0.0	-0.0	-0.0	0.0
KIF14	-0.0	-0.1	0.1	0.0	-0.2	-0.0	-0.0	-0.0	-0.2
KIF20B	0.0	0.0	0.1	-0.0	-0.1	-0.0	-0.0	-0.0	-0.0
KIF23	0.0	0.0	0.0	-0.1	-0.1	-0.0	0.0	0.0	-0.1
MAD2L1	0.1	0.1	0.0	-0.4	-0.2	-0.1	-0.0	0.0	-0.2
NCAPD2	-0.0	-0.0	-0.0	-0.1	-0.1	-0.0	0.0	0.0	-0.0
NCAPG	0.1	0.0	0.0	-0.0	-0.2	-0.1	-0.1	0.0	-0.1
NCAPG2	-0.0	-0.0	0.0	0.0	-0.1	-0.0	0.1	0.0	-0.0
NCAPH	-0.0	0.0	0.1	-0.1	-0.1	-0.1	-0.0	-0.0	-0.0
NDC80	-0.1	0.1	0.0	-0.1	-0.1	-0.0	-0.0	-0.1	0.0
NUP62	0.0	0.1	0.0	-0.0	-0.2	-0.0	0.1	0.1	-0.0
NUSAP1	-0.0	0.0	0.0	-0.1	-0.2	-0.1	-0.1	-0.1	-0.1
RACGAP1	-0.0	-0.0	0.0	-0.0	-0.0	0.0	-0.0	-0.0	0.0
RAD21	0.1	0.1	0.1	-0.1	-0.2	-0.5	0.0	-0.1	-0.2
RANBP1	0.0	-0.0	0.1	-0.1	-0.4	-0.2	-1.9	0.1	-0.3
RRM1	0.0	-0.0	0.1	-0.0	-0.0	-0.1	0.1	-0.0	-0.1
SMC2	0.0	0.1	0.0	-0.0	-0.1	-0.0	0.0	0.1	-0.1
SMC4	0.1	0.2	0.0	-0.0	-0.1	-0.1	0.0	0.1	-0.1
SPAG5	-0.0	0.1	0.0	-0.1	0.0	0.0	0.0	0.0	-0.0
STIL	0.0	0.0	0.0	-0.1	-0.0	-0.1	0.0	-0.2	-0.0
TPR	0.1	-0.0	0.1	0.0	-0.0	-0.0	-0.0	0.1	-1.2

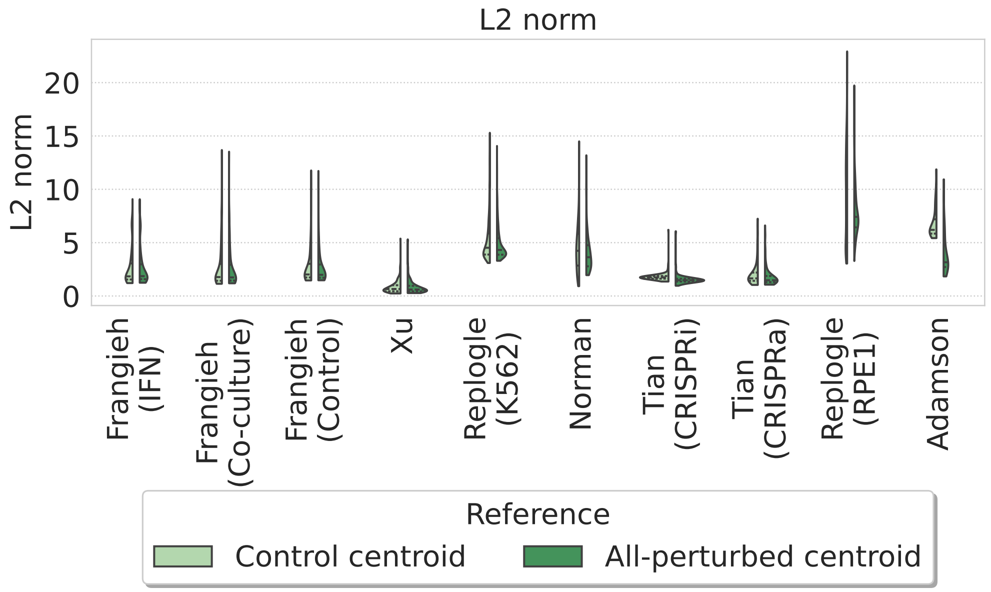

Supplementary Figure 32: Distribution of perturbation-specific shift L2 norms using the control centroid (light green) and perturbed centroid (dark green) references.

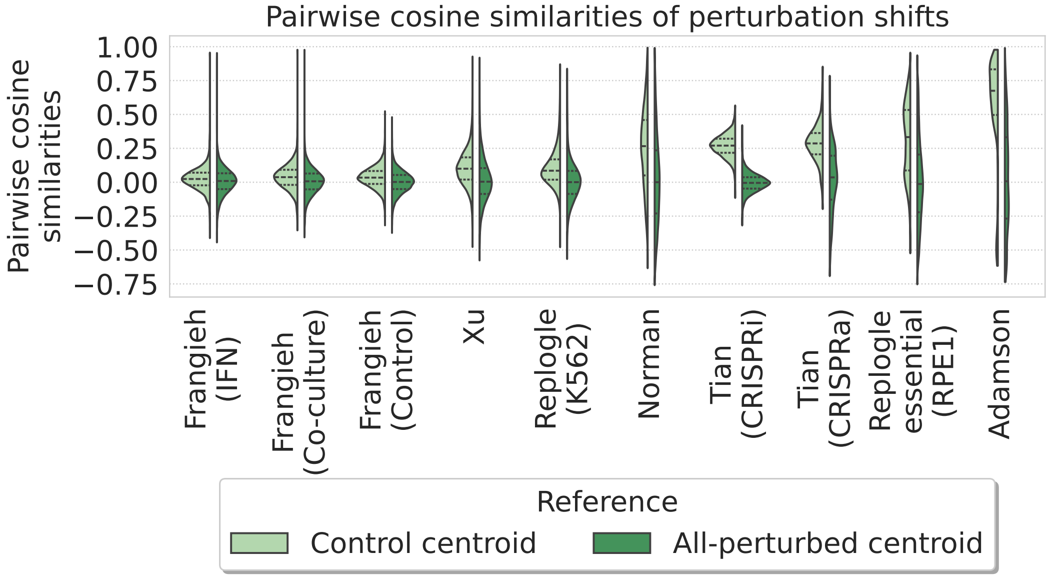

Supplementary Figure 33: Distribution of pairwise cosine similarities between perturbation-specific shifts on ten single-cell perturbation datasets using the control (light green) and perturbed centroid as a reference (dark green). Dashed lines indicate distribution quartiles.

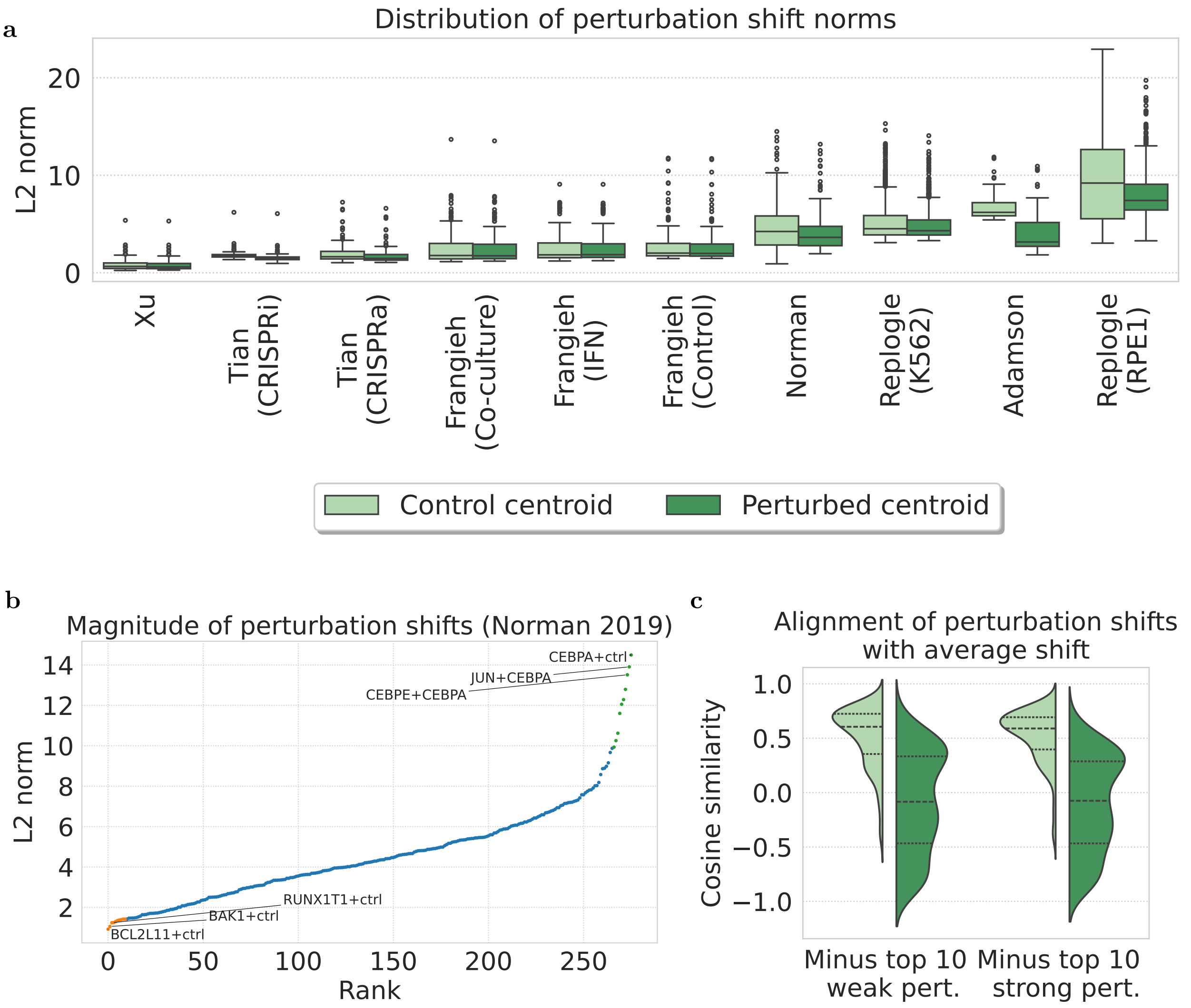

Supplementary Figure 34: Sensitivity analysis of perturbation strength on reference. We quantified perturbation strength by calculating the L2 norm of perturbation-specific shifts. (**a**) Distribution of perturbation-specific shift norms. (**b**) Ranking of perturbation strengths for the Norman et al. (2019) dataset. We studied the sensitivity of the reference to top 10 strongest (green) and top 10 weakest (orange) perturbations. (**c**) Distribution of cosine similarities between perturbation-specific shifts and the average perturbation-specific shift (*i.e.*, difference between all-perturbed centroid and control centroid). We used the perturbed centroid reference computed using all perturbations except the weakest 10 perturbations (left) and except strongest 10 perturbations (right).

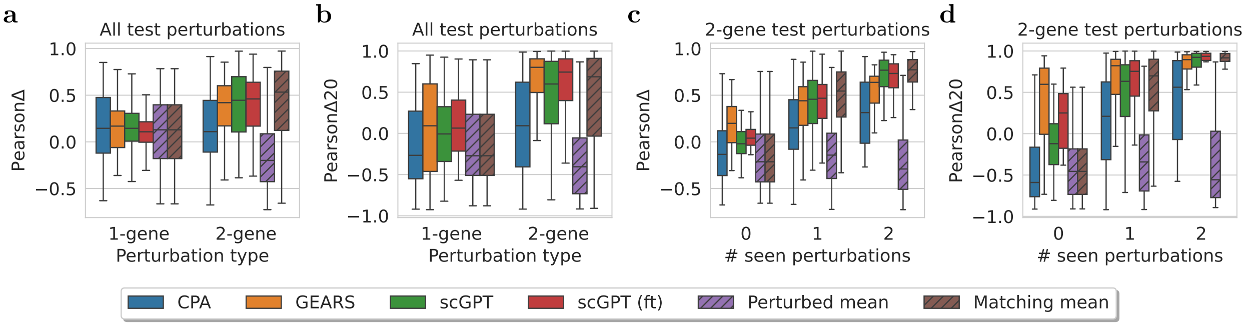

Supplementary Figure 35: Benchmarking perturbation response prediction using the perturbed centroid as a reference. Results are aggregated across 3 independent runs with different data splits. **(a, b)** Prediction performance for 1-gene and 2-gene test perturbations using **(a)** all genes and **(b)** the top 20 differentially expressed genes of each perturbation. **(c, d)** Prediction performance for 2-gene test perturbations by number of matching 1-gene perturbations observed at train time using **(c)** all genes and **(d)** the top 20 differentially expressed genes of each perturbation. (n: number of test perturbations across all three independent runs, Norman: $n = 108$). Boxes depict distribution quartiles, with the center line corresponding to the median, and whiskers span 1.5 times the interquartile range.

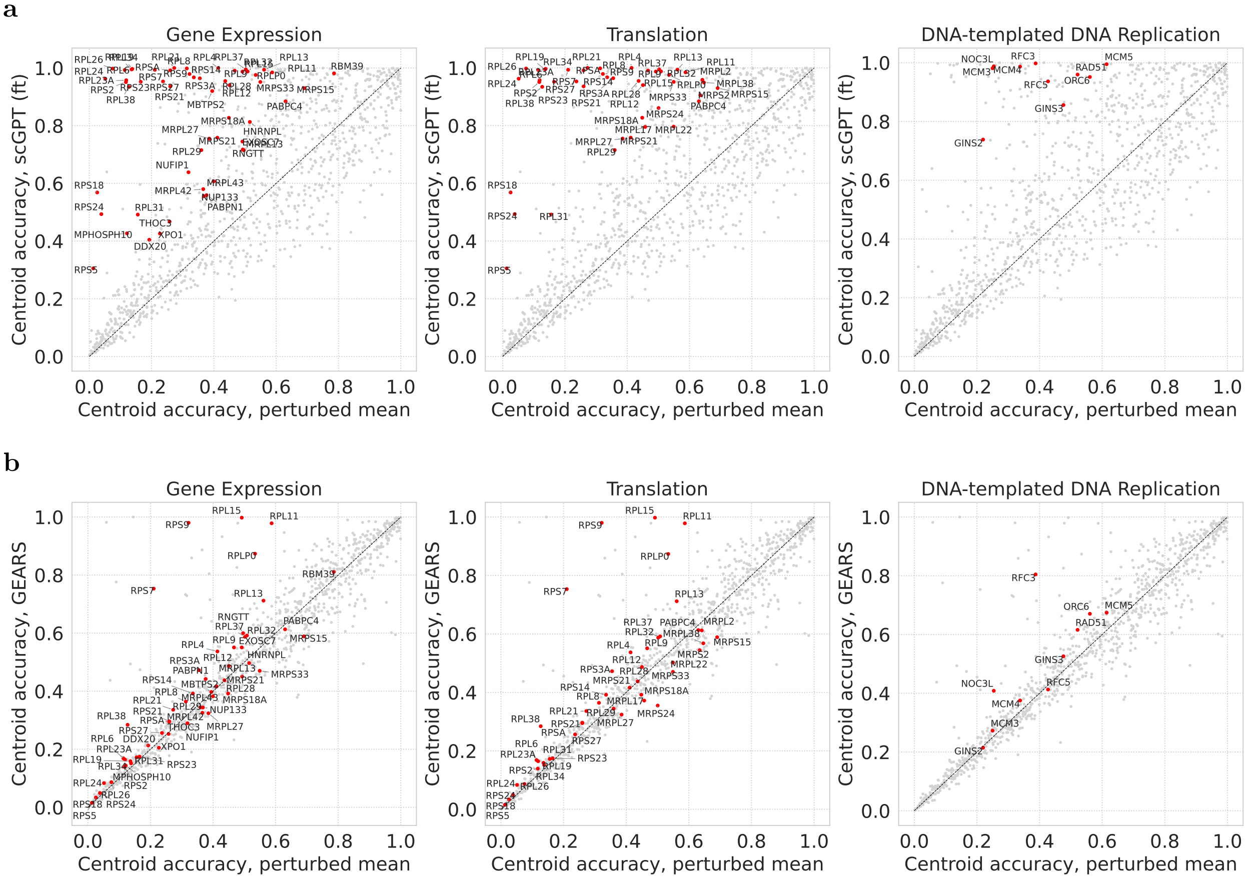

Supplementary Figure 36: Centroid accuracy of **(a)** scGPT versus perturbed mean and **(b)** GEARS versus perturbed mean. We highlighted genes involved in the gene expression, translation, and DNA replication.

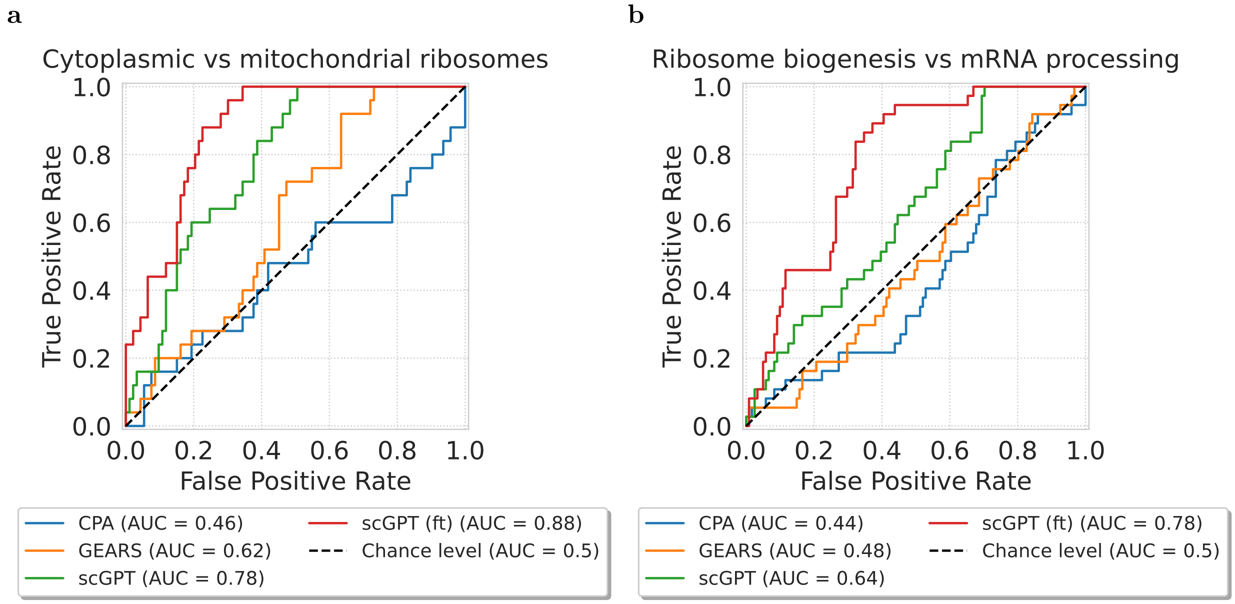

Supplementary Figure 37: Receiver Operating Characteristic curve on downstream coarse-grained classification tasks on Replogle et al. (2022) (K562): **(a)** Classification of perturbations affecting cytoplasmic vs mitochondrial ribosomes and **(b)** Classification of perturbations related to ribosome biogenesis vs mRNA processing. We calculated class-specific centroids by averaging the post-perturbation profiles of the perturbations belonging to each class. For each perturbation, we then calculated distances from the inferred centroid to the class-specific centroids and ranked perturbations based on the difference between the two distances.

We retrieved gene sets from Replogle et al. (2022) (supplementary file: `1-s2.0-S0092867422005979-mmc3.xlsx`) and combined the following perturbation groups:

Cytoplasmic vs mitochondrial ribosomes

- Cytoplasmic ribosome phenotypes:
  - *40S ribosomal subunit, cytoplasmic*
  - *60S ribosomal subunit, cytoplasmic*
- Mitochondrial ribosome phenotypes:
  - *39S ribosomal subunit, mitochondrial*
  - *28S ribosomal subunit, mitochondrial*

Ribosome biogenesis vs mRNA processing

- Ribosome biogenesis:
  - *40S ribosomal subunit, cytoplasmic*
  - *60S ribosomal subunit, cytoplasmic*
  - *39S ribosomal subunit, mitochondrial*
  - *Pol I and rRNA biosynthesis*
- mRNA processing:
  - *spliceosome*
  - *exosome and mRNA turnover*
  - *nonsense-mediated decay*
  - *mRNA capping*
  - *mRNA polyadenylation*
