# Supplementary material for: Systema: a framework for evaluating genetic perturbation response prediction beyond systematic variation
Source: Nat Biotechnol. 2025 Aug 25;44(6):1050–9. doi: 10.1038/s41587-025-02777-8 (PMC13271886; doi:10.1038/s41587-025-02777-8)
Supplement: Supplementary file 2 — Reporting Summary [file 41587_2025_2777_MOESM2_ESM.pdf]

nature portfolio

Corresponding author(s): Maria Brbic

Last updated by author(s): 7/01/2025

Reporting Summary

Nature Portfolio wishes to improve the reproducibility of the work that we publish. This form provides structure for consistency and transparency in reporting. For further information on Nature Portfolio policies, see our Editorial Policies and the Editorial Policy Checklist.

Statistics

For all statistical analyses, confirm that the following items are present in the figure legend, table legend, main text, or Methods section.

n/a	Confirmed	
☐	☒	The exact sample size (*n*) for each experimental group/condition, given as a discrete number and unit of measurement
☐	☒	A statement on whether measurements were taken from distinct samples or whether the same sample was measured repeatedly
☐	☒	The statistical test(s) used AND whether they are one- or two-sided<br>*Only common tests should be described solely by name; describe more complex techniques in the Methods section.*
☐	☒	A description of all covariates tested
☒	☐	A description of any assumptions or corrections, such as tests of normality and adjustment for multiple comparisons
☐	☒	A full description of the statistical parameters including central tendency (e.g. means) or other basic estimates (e.g. regression coefficient) AND variation (e.g. standard deviation) or associated estimates of uncertainty (e.g. confidence intervals)
☐	☒	For null hypothesis testing, the test statistic (e.g. *F*, *t*, *r*) with confidence intervals, effect sizes, degrees of freedom and *P* value noted<br>*Give P values as exact values whenever suitable.*
☒	☐	For Bayesian analysis, information on the choice of priors and Markov chain Monte Carlo settings
☒	☐	For hierarchical and complex designs, identification of the appropriate level for tests and full reporting of outcomes
☐	☒	Estimates of effect sizes (e.g. Cohen's *d*, Pearson's *r*), indicating how they were calculated

Our web collection on statistics for biologists contains articles on many of the points above.

Software and code

Policy information about availability of computer code

Data collection	No software was used for data collection
Data analysis	The source code is available on Github at https://github.com/mlbio-epfl/systema<br><br>We used the following Python packages:<br>adjustText==1.0.4<br>anndata==0.9.2<br>blitzgsea==1.3.40<br>cell-gears==0.1.2<br>cpa-tools==0.8.8<br>gseapy==1.1.2<br>h5py==3.10.0<br>ipykernel==6.29.0<br>ipython==8.21.0<br>matplotlib==3.8.2<br>networkx==3.2.1<br>numpy==1.23.5<br>pandas==2.2.2<br>scanpy==1.9.8<br>scikit-learn==1.4.0<br>scipy==1.12.0

```
seaborn==0.13.2
statsmodels==0.14.1
torch==2.0.0
torch_geometric==2.5.1
tqdm==4.66.1
umap-learn==0.5.5
wandb==0.17.0
flash-attn==1.0.4
scgpt==0.2.1
```

For manuscripts utilizing custom algorithms or software that are central to the research but not yet described in published literature, software must be made available to editors and reviewers. We strongly encourage code deposition in a community repository (e.g. GitHub). See the Nature Portfolio guidelines for submitting code & software for further information.

Data

Policy information about availability of data

All manuscripts must include a data availability statement. This statement should provide the following information, where applicable:

- Accession codes, unique identifiers, or web links for publicly available datasets
- A description of any restrictions on data availability
- For clinical datasets or third party data, please ensure that the statement adheres to our policy

We downloaded and processed data using the GEARS (Roohani et al., 2023) codebase. The Gene Expression Omnibus accession numbers used are: Adamson et al. (2016): GSE90546, Norman et al. (2019): GSE146194, Xu et al. (2024): GSE218566. The data from Replogle et al. (2022) are available at: https://doi.org/10.25452/figshare.plus.20022944 and additional annotations are available at: https://doi.org/10.25452/figshare.plus.21632564. The Frangieh et al. (2021) data are available at: https://singlecell.broadinstitute.org/single_cell/study/SCP1064/multi-modal-pooled-perturb-cite-seq-screens-in-patient-models-define-novel-mechanisms-of-cancer-immune-evasion. The Tian et al. (2019) data are available via scPerturb (Peidli et al., 2024) at: https://doi.org/10.5281/zenodo.13350497.

Research involving human participants, their data, or biological material

Policy information about studies with human participants or human data. See also policy information about sex, gender (identity/presentation), and sexual orientation and race, ethnicity and racism.

Reporting on sex and gender	We did not perform any sex-based analyses because this information is not provided in the publicly available data.
Reporting on race, ethnicity, or other socially relevant groupings	We did not perform any race-based analyses because this information is not provided in the publicly available data.
Population characteristics	See above
Recruitment	NA
Ethics oversight	NA

Note that full information on the approval of the study protocol must also be provided in the manuscript.

Field-specific reporting

Please select the one below that is the best fit for your research. If you are not sure, read the appropriate sections before making your selection.

☒ Life sciences ☐ Behavioural & social sciences ☐ Ecological, evolutionary & environmental sciences

For a reference copy of the document with all sections, see nature.com/documents/nr-reporting-summary-flat.pdf

Life sciences study design

All studies must disclose on these points even when the disclosure is negative.

Sample size	We used published datasets, so sample size was defined by the studies that generated the data and any relevant post-processing. We used 3 different train-test splits, running the models 3 different times.
Data exclusions	We used the codebase from Roohani et al. (2023) to process the data. We discarded perturbations corresponding to genes not in the gene panel. This step is necessary for running scGPT, i.e., scGPT's architecture was designed to handle perturbations of genes with available transcriptomic readouts. Following GEARS, we then selected the top 5,000 highly

variable genes (HVGs) in each dataset (Roohani et al., 2023), and included the set of perturbed non-HVGs to the gene panel. For the Replogle et al. (2022) datasets, we followed the data processing strategy of Cui et al. (2024): we retained the subset of data matching the 1,973 perturbations identified in the original study (Replogle et al., 2022) as inducing strong transcriptional changes, and then selected 100 cells per perturbation and 2,500 control cells. In terms of Replogle et al. (2022) K562, we used the genome-wide K562 perturbation screen. In terms of the Tian et al. (2019) data, we discarded the perturbations PPP4R3A (SMEK1 ; CRISPRa dataset) and ATP5PD (ATP5H ; CRISPRi dataset) that targeted only a single cell. To process the Frangieh et al. (2021) data, we followed the processing steps of Lopez et al. (2022), i.e. we first converted the data from log-normalized count per millions into raw counts and removed cells with less than 500 expressed genes and genes expressed in less than 500 cells. We then selected cells perturbed with guides that exclusively target 1 gene.

Replication: We provide the code of our benchmark for reproducibility

Randomization: Not required as we were not running any new gene perturbation experiments. Controlling covariates is not applicable to the study because we do not generate new data and we benchmark existing computational methods as they were originally designed.

Blinding: Not required as we were not running any new gene perturbation experiments

Reporting for specific materials, systems and methods

We require information from authors about some types of materials, experimental systems and methods used in many studies. Here, indicate whether each material, system or method listed is relevant to your study. If you are not sure if a list item applies to your research, read the appropriate section before selecting a response.

Materials & experimental systems

n/a	Involved in the study	
☒	☐	Antibodies
☒	☐	Eukaryotic cell lines
☒	☐	Palaeontology and archaeology
☒	☐	Animals and other organisms
☒	☐	Clinical data
☒	☐	Dual use research of concern
☒	☐	Plants

Methods

n/a	Involved in the study	
☒	☐	ChIP-seq
☒	☐	Flow cytometry
☒	☐	MRI-based neuroimaging

Plants

Seed stocks: NA

Novel plant genotypes: NA

Authentication: NA
